# Single-cell approach dissecting *agr* quorum sensing dynamics in *Staphylococcus aureus*

**DOI:** 10.1038/s41467-026-73552-9

**Published:** 2026-05-20

**Authors:** Julian Bär, Mariane Pivard, Samuel G. V. Charlton, Andrew G. Kempchinsky, Alejandro Gómez-Mejia, Giovanni Stefano Ugolini, Srikanth Mairpady Shambat, Eleonora Secchi, Annelies S. Zinkernagel

**Affiliations:** 1 Department of Infectious Diseases and Hospital Epidemiology, University Hospital Zürich, University of Zürich, Zürich, Switzerland; 2 Department of Civil, Environmental and Geomatic Engineering, ETH Zurich, Zurich, Switzerland

**Keywords:** Pathogens, Bacteria, Cellular microbiology

## Abstract

Quorum sensing (QS) enables bacteria to coordinate collective behaviors by secreting and sensing diffusible signals. Understanding QS at single-cell resolution is essential because population-level measurements often obscure regulatory heterogeneity. In *Staphylococcus aureus*, the accessory gene regulator (*agr*) system is a major QS-controlled virulence regulator activated by autoinducing peptides (AIPs). Four *agr-*types exist, each defined by distinct AIPs and capable of cross-inhibition, yet their activation dynamics and interaction hierarchies remain poorly understood. Using microfluidics, time-lapse microscopy, and deep-learning-based image analysis, we quantified *agr*-activation in congenic and native *agr*-type strains. *Agr*-types differed in sensitivity to their homologous AIPs: *agr*-III was largely unresponsive, whereas *agr*-IV was highly sensitive with elevated basal activation. *Agr*-activation distribution was frequently bimodal (simultaneous existence of *agr*-ON and *agr-*OFF cells), driven by subpopulations that never activated or switched to *agr*-OFF despite constant stimulation. Combining homologous and heterologous AIPs, AIP‑IV suppressed pre‑activated *agr*-I, while AIP-I had no inhibitory effect on *agr*‑IV, indicating asymmetric cross‑inhibition. In spatially segregated cocultures, diffusional crosstalk resulted in four reproducible *agr*-interaction regimes, stable-dominance, stable-, delayed-, or unstable-dual-activation, determined by *agr*-type pairing and influenced by genetic background. Our approach links single-cell signaling to population outcomes and uncovers *agr-*type-specific asymmetries with potential consequences for strain competition and virulence.

## Abstract (web summary)

Various types of the *agr* quorum-sensing system, defined by distinct autoinducing peptides, regulate virulence in *Staphylococcus aureus*. Here, Bär et al. use microfluidics and single-cell imaging to show that *agr* types differ in signal-peptide sensitivity and activation heterogeneity, and that cross-inhibition between types produces distinct interaction behaviours.

## Introduction

*Staphylococcus aureus* is a major human pathobiont, causing infections ranging from mild skin lesions to life-threatening endocarditis and prosthetic joint infections^1,2^. A key driver of *S. aureus* pathogenesis is its capacity to produce a wide array of virulence factors, such as exotoxins, that enable immune evasion and tissue damage. During colonization and chronic infections, toxin production is typically reduced but is activated during acute invasive disease^3–5^. The *accessory gene regulator* (*agr*) system is pivotal for virulence and toxin regulation^6,7^.

The two-component quorum-sensing (QS) *agr-*system senses population density via secreted autoinducing peptides (AIPs)^7,8^. It comprises two transcripts, RNAII and RNAIII, controlled by the promoters P2 and P3, respectively^6^. RNAII is the transcript of the *agr-*operon, composed of the four genes required for AIP production (a*grD*, precursor), secretion (*agrB*, exporter) and sensing (*agrC*, sensor histidine kinase), as well as response regulation (*agrA*, transcription factor). RNAIII acts as the downstream small RNA effector, regulating translation of virulence genes and regulators, and encodes for the delta-toxin (Hld). Extracellular AIP sensing by AgrC triggers AgrA phosphorylation, which then activates P2, driving a positive feedback loop upregulating RNAII expression and therefore increasing AIP secretion. At high extracellular AIP concentrations, phosphorylated AgrA additionally activates P3 and directly upregulates phenol-soluble modulins (PSMs) production^9^. Activation of the *agr-*system, indicated by P3-driven RNAIII expression, induces expression of toxins such as alpha-hemolysin (Hla) and leukocidins, and is associated with a switch from an avirulent to a virulent, dispersal-related gene expression profile^6,10^.

Four *S. aureus agr*-types are defined by unique AIP sequences (7–9 amino acids) and polymorphisms in the signal transduction genes (*agrDBC*). *Agr-*types show strict signaling specificity, with activation occurring only in response to homologous AIP^11^. Heterologous AIPs generally act as inhibitors, although cross-activation between types I and IV has been observed, likely due to a single amino acid difference in their AIPs^12–14^. *Agr*-type is tightly linked to genetic background, as most sequence types (ST) harbor only one *agr*-type, reflecting the need for coordinated mutations across multiple *agr*-genes during evolution^15–17^. This tight linkage has historically hindered meaningful comparisons between *agr*‑types, as strains carrying different *agr*-types usually differ in many other genomic features, making it difficult to distinguish *agr*‑dependent effects from genetic background‑specific ones. *Agr*-types have also been associated with distinct toxin profiles^11^, but it remains unclear which differences arise directly from *agr*-type versus broader genotype-specific factors.

Single-cell studies are essential to resolve QS heterogeneity, which bulk assays mask by averaging across populations. QS heterogeneity, or bimodality, refers to the coexistence of QS-activated and QS-inactivated cells within a single population^10,18,19^. Prior reports of this phenomenon were derived indirectly by quantifying single cells isolated from bulk cultures, where accumulation or uneven distribution of QS signals may create spatial effects that mimic heterogeneity. Because QS output is also shaped by physiological state and environmental factors, such as pH and nutrient availability^19,20^, it remains unclear whether the observed heterogeneity reflects properties of the QS system itself or arises from external environmental variation.

While single‑cell approaches to clarify heterogeneity within populations are available, experimental tools to examine QS interactions between bacterial populations remain scarce^21,22^. This gap limits our ability to determine how different *agr*‑types respond when confronted with competing strains or species producing heterologous AIPs.

To eliminate biases of genetic background, we used a set of four congenic-*agr* strains constructed by Geisinger et al.^13^, where the native *agr*-system was replaced with any of the four *agr*-types. To assess whether observed results in these congenic strains reflect a general property of *agr*-type, we additionally analyzed four commonly used laboratory strains, each carrying its native *agr-*system and representing a different genetic background. We combined microfluidics, time-lapse microscopy, and deep-learning-based image analysis to quantify *agr*-activation dynamics and QS heterogeneity across all four *agr*-types. Mother-machine microfluidics and synthetic AIPs provide a chemically stable and spatially homogeneous environment, allowing unprecedented accuracy to investigate links between QS and bacterial physiology. We then tested how heterologous AIPs suppress pre-activated *agr-*systems, attributable exclusively to AIP interactions, allowing nuanced interpretation of published bulk experiments. Finally, we explored community-level crosstalk in spatially structured co-cultures of different *agr*-types.

## Results

### Mother-machine microfluidic devices for single-cell *agr* quantification

**Fig. 1:**
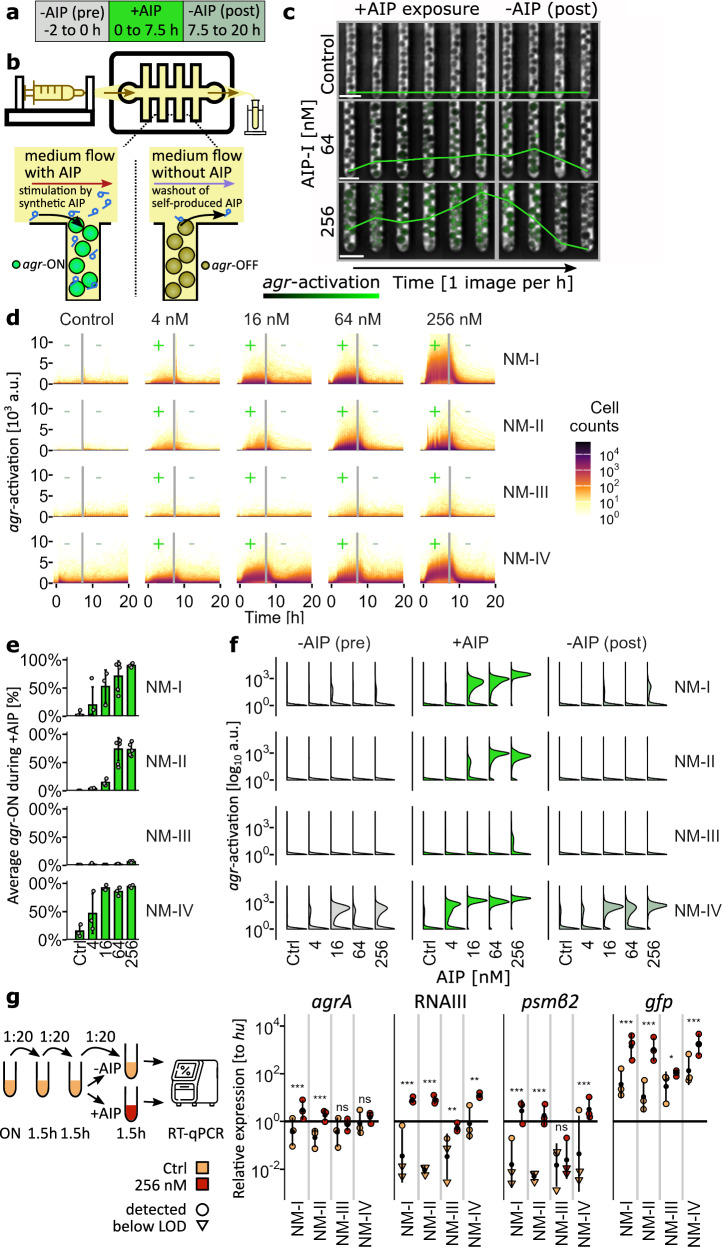
*agr*-type determines AIP sensitivity and *agr-*activation heterogeneity. **a** Experimental design: bacteria were transiently exposed for 7.5 h to synthetic, homologous AIP in mother-machine microfluidic chips. Box fill color reflects experimental phases used also in (**e**, **f**). Each experiment was repeated at least three times. **b** Schematic of the mother-machine chip (top) and embedded bacteria (bottom). Growth medium was supplied at 0.5 ml h^−^¹ using a syringe pump to wash out self-produced AIP and excess bacteria, ensuring stimulation only by synthetic AIP. *Agr-*activation was monitored using P3-*gfp* reporter plasmids. **c** Representative image sequences of *S. aureus* NM-I exposed to three AIP-I concentrations. Control replicates received 2.5% PBS in place of AIP. Phase-contrast images are overlaid with GFP fluorescence (both contrast adjusted for visualization). The vertical gray line marks AIP removal, and the green lines show average single-cell GFP intensity. Scale bar, 5 µm. **d** Single-cell *agr*-activation (GFP intensity in arbitrary units (a.u.)) during synthetic, homologous AIP exposure (concentration indicated above each column) and after AIP removal (gray lines, plus/minus symbols). AIP exposure started at time = 0, and the preceding period was used for (**e**) as -AIP (pre). Each row represents one *agr*-type. Dot color reflects log₁₀ cell counts. Cells from all replicates were pooled and, on average, 3,003,583 cells per condition were analyzed (Supplementary Table 1). **e** Average Percentage of *agr*-ON cells (threshold: 100 AU) during AIP exposure. Each dot represents one biological replicate (*N* = 3–7 independent mother-machine experiments inoculated from cultures obtained on separate days, details in Supplementary Table 1); bars show mean ± SD. **f** Histograms of log₁₀ *agr-*activation during defined intervals of the same data shown in (**d**): -AIP (pre), 2 h before exposure start (−2 to 0 h); +AIP, final 2 h of AIP exposure (5.5–7.5 h); -AIP (post), 3–5 h after AIP removal (10.5–12.5 h). **g** RT-qPCR on the third 1.5 h exponential-phase subculture, either stimulated with 256 nM synthetic AIP (red) or control medium (yellow). Relative expression normalized to the housekeeping gene *hu* is shown for four genes. Colored dots represent replicates (*N* = 3 biological replicates from independent cultures, performed on separate days); black bars indicate mean ± SD. Samples with undetectable target expression (limit-of detection (LOD): Ct ≥ 30) were assigned the LOD Ct for ΔCt/ΔΔCt computation and are shown as triangle symbols. Statistical comparisons between unstimulated and AIP-stimulated conditions were performed per gene using a linear mixed-effects model with log₁₀-transformed relative expression as outcome, a fixed effect for strain × AIP concentration, and a random intercept for biological replicate. Two-sided pairwise comparisons (256 nM vs. control) were obtained using estimated marginal means. Significance is indicated as ns (*p* ≥ 0.05), * (*p* < 0.05), ** (*p* < 0.01), and *** (*p* < 0.001). Exact two-sided *p*-values are reported in Supplementary Table3. qPCR machine icon by flaticon.com.

We used mother-machine microfluidic devices to dynamically switch between media with or without synthetic, homologous AIP (Fig. 1a). Mother-machine chips feature central flow channels and perpendicular side chambers that trap bacteria (Fig. 1b), enabling continuous growth without cell aggregation as excess bacteria are washed out. Constant medium flow further ensures that self-produced AIP cannot accumulate. Although mother-machines are commonly used to hold rod-shaped bacteria in a strict single-file arrangement, coccoid *S. aureus* cells are often arranged into a 2D distribution with cells side-by-side (Fig. 1c). Despite this, cells remained confined to one imaging plane, permitting single-cell image analysis.

We used congenic *S. aureus* Newman strains (ST8) in which the native *agr-*locus was replaced with one of the four *agr*-types^13^ (abbreviated NM-I, -II, -III, and -IV). *Agr*-activation was monitored using microscopy and P3-*gfp* reporter plasmids generating fluorescence upon *agr-*activation (Fig. 1c, example movies in Supplementary Data 1). Single-cell GFP intensities were extracted with a custom image analysis pipeline utilizing StarDist2D^23^ and DeLTA2.0^24^, retrained on novel *S. aureus* data to ensure accurate coccoid cell segmentation and tracking (details in “Methods”).

We validated reporter performance and AIP activity in bulk cultures using a plate-reader, confirming a functional *agr*-system in all strains as well as the modulation of *agr*-activation strength and timing upon the addition of synthetic, homologous AIP (Supplementary Fig. 1a–f).

### *agr*-type determines AIP sensitivity and *agr*-activation heterogeneity

We quantified *agr*-activation during 7.5 h transient exposure to 0–256 nM synthetic homologous AIP under continuous nutrient-rich flow in the mother-machine. In the absence of synthetic AIP (control, 2.5% phosphate-buffered-saline (PBS)), NM-I, NM-II, and NM-III showed no detectable *agr-*activation (GFP signal), indicating that self-produced AIP remained below their P3 activation thresholds (Fig. 1d). However, NM-IV exhibited elevated basal *agr*-activation. This pattern was also evident when we binarized single‑cell data into *agr*‑OFF and *agr*‑ON states (100 a.u. GFP threshold): 19% of unstimulated NM‑IV cells were *agr*-ON, compared to 2, 0.2, and 0.9% for NM‑I, NM‑II, and NM‑III (Fig. 1e). This binary classification confirms that NM‑IV’s elevated basal *agr*-activation reflects a substantial activated subpopulation rather than a marginal tail of the GFP distribution.

We detected pronounced differences in AIP sensitivity among the four *agr*-types when transiently exposing them to synthetic, homologous AIP in mother-machines. Upon AIP addition, NM-I and NM-II displayed dose-dependent activation, whereas NM-IV plateaued at full activation (>95% *agr-*ON) from 16 nM on (Fig. 1d–f). Full *agr*-activation of NM-I required 256 nM, and NM-II never reached more than 88% *agr*-ON even at the highest tested concentration (Fig. 1e, Supplementary Fig. 2, and Supplementary Movie 1). NM-III remained unresponsive up to 256 nM, where only a small fraction of cells started to respond (max. *agr*-ON 12 ± 3%, Fig.1e), consistent with its delayed autoinduction in bulk cultures where self-produced AIP can accumulate (Supplementary Fig. 1c).

Distributions of single-cell *agr-*activation in these mother-machine experiments were bimodal at specific observation intervals corresponding to the three experimental phases (Fig. 1f). Here, bimodal refers to two distinct peaks in the histograms, representing *agr*-OFF and *agr*-ON cells, which revealed pronounced QS heterogeneity at low to intermediate AIP concentrations (4–64 nM). The relative peak heights depended on both *agr*-type and AIP concentrations, with higher AIP levels shifting more cells into the *agr-*ON state.

Similar patterns were observed in native-*agr* laboratory strains with distinct genetic backgrounds (LAC-I, ST8; 502a-II, ST5; MW2-III, ST1 and MNTG-IV, ST2276) with an alternative P3-*yfp* reporter construct^7^ (Supplementary Fig. 3a–c and Supplementary Movie 2, example movies in Supplementary Data 2). The LAC-I and 502a-II strains showed concentration-dependent *agr-*activation, MW2-III barely responded to its homologous AIP, MNTG-IV exhibited elevated unstimulated basal *agr*-activation and high AIP sensitivity, and bimodal *agr*-activation was observable across all responsive *agr*-types.

The response time of the *agr-*system to AIP stimulation of NM-I, -II and -IV was largely unaffected by AIP concentration but varied among *agr*-types. NM‑IV responded rapidly and consistently (average time to reach 50% *agr*‑ON, 0.6 h), NM‑I was slower (1.4 h), and NM‑II slowest (2.5 h, Supplementary Fig. 4a). Neither NM-III nor the native MW-III reached the threshold of 50% *agr*-ON; therefore, no response time could be calculated (Supplementary Fig. 3d and Supplementary Fig. 4a). Native strains LAC-I, 502a-II and MNTG-IV responded with similar speed but with AIP concentration dependency for LAC-I and 502a-II (Supplementary Fig. 3d).

Higher AIP concentrations significantly prolonged the *agr*-ON state after AIP withdrawal (time until less than 50% *agr*-ON) in NM-IV (increased from 3.3 h at 4 nM to 15.4 h at 256 nM) while no such prolongation was recorded for NM-I and NM-II (Fig. 1f and Supplementary Fig. 4b). A similar AIP‑concentration-dependent prolongation was observed in the native strains LAC-I, 502a-II, and MNTG-IV, albeit with varying statistical strength (*p* = 0.062 for LAC‑I; *p* < 0.01 for 502a‑II and MNTG‑IV, Supplementary Fig. 3e). In contrast, NM‑I and NM‑II deactivated rapidly independent of AIP concentration (Supplementary Fig. 4b).

To determine whether the P3 promoter activation differences observed in microfluidics arose from pre-existing transcriptional variation rather than AIP sensitivity, we compared global gene expression profiles across the congenic strains. RNA-seq of early exponential cultures under mother-machine mimicking nutrient-rich, AIP-free conditions revealed uniformly low *agr*-locus expression and undetectable levels of *agr*-regulated genes (e.g., *psm*), with no significant differences between strains (Supplementary Fig. 5a–c). These data indicate that the microfluidic activation differences are not explained by differential baseline transcription.

To validate that GFP accurately reports *agr*-activation, we quantified expression of *agrA*, RNAIII, *psmβ2*, and the plasmid-encoded *gfp* by RT-qPCR in early exponential cultures with and without 256 nM synthetic AIP stimulation. AIP stimulation increased all four transcripts without affecting growth (Fig. 1g and Supplementary Fig. 6a). Consistent with single-cell observations, NM-III showed the lowest expression under both conditions, whereas NM-IV exhibited elevated basal expression and was the only strain with consistently detectable RNAIII in the absence of synthetic AIP. Correlation analysis between *gfp* and either *agrA*, RNAIII, or *psmβ2* confirmed that GFP provides a reliable proxy for *agr-*activation (Supplementary Fig. 6b–d), excluding reporter artifacts. Finally, these same early exponential cultures for qPCR were transferred to a plate reader. The AIP-stimulated populations displayed earlier and stronger GFP fluorescence than unstimulated controls (Supplementary Fig. 6e, f). This confirmatory experiment demonstrates that transcriptional differences translate into measurable fluorescence at the population level and reflect the activation hierarchy observed in microfluidics.

Together, RNA-seq and qPCR independently validate that *agr*-type intrinsically determines AIP sensitivity and *agr*-activation kinetics.

### Fitness impacts by AIP exposure differ between congenic and native-*agr* strains

**Fig. 2:**
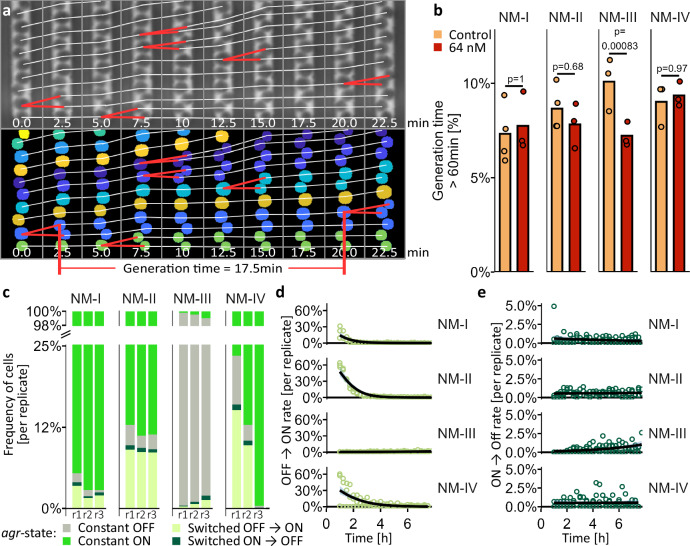
Single-cell dynamics of *agr-*activation from division to division explain population-level bimodality. **a** Representative image sequence of phase-contrast (top) with segmentation and tracks (bottom). Tracks are color-coded across frames; white lines connect track segments, and red lines mark division events. Generation time (interval between divisions) is shown for one cell. **b** The proportion of bacteria with prolonged generation time during the exposure phase was calculated per replicate based on the threshold of 60 min for control (yellow) and 64 nM stimulated bacteria (red). *P*-values are based on a generalized linear mixed-effects model with estimated marginal means and post hoc tests. Bars show means; dots show biological replicate values. **c**–**e** Show data for 64 nM synthetic AIP. **c** Single-cell *agr* dynamics (per cell from division to division) were classified as constant *agr*-ON (GFP threshold of 100 a.u., bright green), constant *agr*-OFF (gray), or switching (OFF → ON, light green, ON → OFF, dark green). All cells alive during the exposure phase were considered. The axis is broken to display the first 25% and the last 2%. One stacked bar represents one biological replicate (r1, r2, and r3). **d**, **e** Time-dependent analysis of switching events from the same dataset as in (**c**). **d** OFF → ON transitions; **e** ON → OFF transitions. The x-axis indicates the time of cell division (end of cell cycle); black lines represent binomial regression fits. Colored dots show time-resolved per-replicate data. *N* = 3; average of 100,755 cells per condition (Supplementary Table 1) for all panels.

QS-activation in *S. aureus* normally occurs at high cell density, where nutrient limitation couples metabolism to virulence regulation. In contrast, our microfluidic setup supplies a constant nutrient-rich medium, allowing *agr*-activation solely by synthetic AIP. This controlled environment enabled assessing whether AIP exposure and *agr*-activation, independent of nutrient stress, affect bacterial fitness. We tracked individual cells with observed birth and division events and calculated generation time as the interval between these events, while continuously monitoring *agr*-activation throughout each cell’s lifespan (Fig. 2a).

AIP exposure did not increase the proportion of delayed divisions (generation time > 60 min) in the responding *agr*-types (NM-I, -II, -IV; Fig. 2b). NM-III, which did not activate *agr*, showed a significant reduction in delayed divisions upon AIP exposure (Fig. 2b). Generation times without AIP stimulation did not differ significantly among congenic strains (mixed-effects model, *p* = 0.25, Supplementary Fig. 7a), confirming that observed changes were AIP-dependent.

Native-*agr* strains had statistically different generation times without AIP stimulation, with 502a-II being the slowest and MW2-III the fastest, illustrating a genetic background-dependent effect on cellular physiology (Supplementary Fig. 7b). Upon AIP exposure, LAC-I, 502a-II, and MNTG-IV exhibited significantly increased division delays, reaching 12%, 61%, and 30%, respectively (Supplementary Fig. 7c), with a disproportionately strong fitness impact in 502a-II. Cells with delayed division were consistently larger across all congenic and native strains (Supplementary Fig. 7d, e), indicating a physiological shift.

### Rare *agr*-state switching and *agr*-OFF state persistence underlie bimodal *agr*-activation

We quantified *agr*-activation of bacteria over their lifespan (Fig. 2a) to investigate if the observed bimodality of *agr-*activation, characterized by the presence of *agr*-OFF and *agr-*ON cells (Fig. 1f), could arise from frequent switching between *agr*-states at the single-cell level. During exposure to 64 nM synthetic AIP, a large fraction of NM-I, -II, and -IV cells remained constantly *agr*-ON during their individual lifespans (96, 88 and 88%, respectively, Fig. 2c). A small fraction remained constantly *agr*-OFF (0.9, 2, 0.3% for NM-I, -II and -IV, respectively), and a similar small fraction switched from *agr*-ON to *agr*-OFF despite continuous AIP exposure (0.4, 0.6, and 0.5% for NM-I, -II and -IV, respectively, Fig. 2c). OFF → ON transitions occurred predominantly within the first 4 h of AIP exposure, consistent with expected rapid AIP response (Fig. 2d). ON → OFF switching events remained rare and showed no consistent time-dependent pattern during AIP exposure in both congenic (Fig. 2e) and native strains except for MNTG-IV which had slightly more ON→OFF switches early during AIP exposure phase (Supplementary Fig. 7f–h).

### *agr*-ON showed higher intergenerational stability than *agr*-OFF under constant AIP exposure

In the previous section, we examined how single-cells transitioned between *agr-*states during their own lifespan. We now asked a complementary question: does *agr*-state persist across multiple generations, i.e., is there evidence of phenotypic heritability? Specifically, we tested whether the small population of non-responders (cells that remained *agr*-OFF despite AIP exposure) occurred randomly or clustered within lineages, which would indicate intergenerational stability of the *agr*-OFF state.

**Fig. 3:**
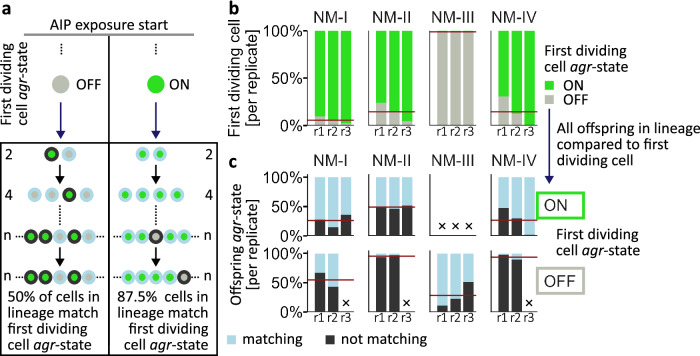
During AIP exposure, the *agr*-ON state exhibits greater intergenerational stability than the *agr*-OFF state. **a** Lineage trees were constructed for the first-dividing cell and their *agr*-state (the first division event recorded per lineage after AIP exposure start, gray: *agr*-OFF, green: *agr*-ON), and the *agr*-state of all offspring (based on GFP signal at division time) was quantified within the exposure window (blue: matching to first-dividing cell *agr*-state, dark gray: not matching to first-dividing cell *agr*-state). Example calculations are annotated for the drawn lineage. The same color scheme are used in (**b**,** c**). **b** Stacked bars of *agr-*state of first-dividing cells per replicate (r1, r2, and r3). Red horizontal lines indicate the average per strain. **c** Proportion of offspring per lineage retaining the same *agr-*state as the first-dividing cell (blue, matching first-dividing cell; black, not-matching first-dividing cell), shown separately for *agr*-ON (top, green in Fig. 2c) and *agr*-OFF (bottom, gray in Fig. 2c) first-dividing cells and by replicate. Red horizontal lines indicate the average per strain and the first dividing cell state. Crosses indicate replicates where <6 first-dividing cells in the respective *agr-*state were present (see **b**); therefore, offspring stability could not be assessed for that state. *N* = 3; average of 100,755 cells per condition (Supplementary Table 1) for all panels.

To address this, we tracked *agr*-states across cell lineages during constant AIP exposure. We focused on cells which started dividing shortly after AIP exposure, referred to as first-dividing cells, and their offspring (up to 10 generations, Fig. 3a). Most of the first-dividing cells of NM-I, -II, and -IV were *agr*-ON, indicating rapid activation in response to AIP while NM-III was almost entirely *agr*-OFF (Fig. 3b). The offspring of NM-I and -IV largely retained the *agr-*ON state (74 ± 11% and 73 ± 23%, respectively), demonstrating strong intergenerational stability, while NM-II had less matched *agr*-state offspring (51 ± 2%, Fig.3c). Lineages of the three responding NM-I, -II and -IV originating from *agr*-OFF frequently switched to *agr-*ON during AIP exposure (55, 95 and 93% for NM-I, -II and -IV, respectively), indicating low intergenerational stability of the *agr*-OFF state but with variability between the strains (Fig. 3c). Most NM-III offspring retained the *agr*-OFF state (72%).

The intergenerational stability of *agr*-ON was slightly lower in native LAC-I but higher in 502a-II and MNTG-IV compared to the respective congenic *agr*-type (Supplementary Fig. 7i, j), suggesting that genetic background influences how *agr*-states are maintained across generations.

These dynamics suggest that QS bimodality reflected a combination of stable *agr*-ON lineages and rare switching, while persistent *agr*-OFF lineages formed a minority under AIP exposure. The stabilization of *agr*-ON in *agr*-I, -II and -IV under sustained AIP signal is consistent with the positive-feedback architecture of the *agr-*system, whereas rare ON → OFF events may reflect stochastic fluctuations.

### Heterologous AIPs rapidly suppress pre-activated *agr-*systems at single-cell resolution

**Fig. 4:**
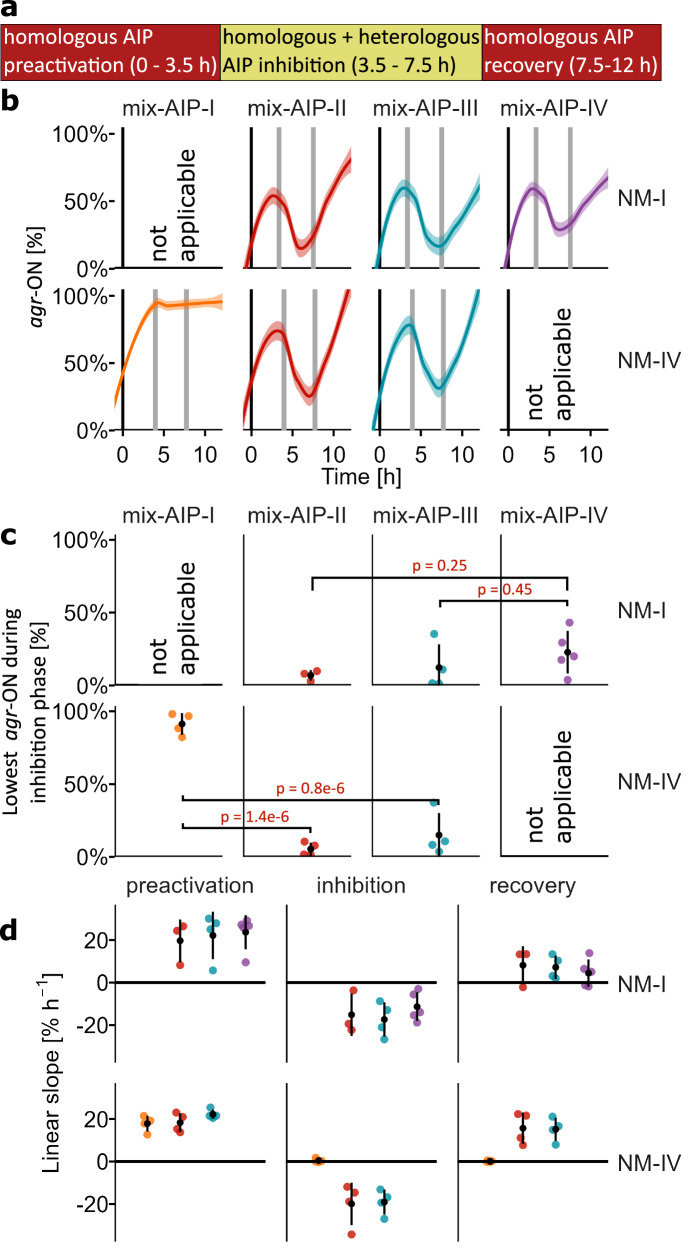
Heterologous AIPs suppress pre-activated *agr-*systems. **a** Experimental design: NM-I and NM-IV were sequentially exposed to 32 nM homologous AIP (pre-activation), a mixture of homologous and heterologous AIPs (both 32 nM, total 64 nM) and back to 32 nM homologous AIP alone. **b** Percentage of *agr*‑ON cells per biological replicate over time; vertical gray lines mark AIP switches. Percentages were calculated per biological replicate and time. Lines show LOESS‑smoothed trends, with shaded regions indicating pointwise 95% confidence intervals of the LOESS fit, colored by AIP type during the inhibition phase. On average, 1,897,476 cells per condition were analyzed (Supplementary Table4). **c** Lowest percentage of *agr*-ON cells during heterologous inhibition phase. Colored dots represent replicates; black dots and bars show mean ± SD. Statistical significance was assessed using two‑sided Dunnett’s tests with AIP‑IV and AIP‑I as references for NM‑I and NM‑IV, respectively. **d** The slope of per-replicate linear regression of the percentage of *agr*-ON is shown for the three experimental phases. Colored dots represent replicates; black dots and bars show mean ± SD. As mix-AIP-I for NM-IV reached full activation in the preactivation phase and did not get inhibited by AIP-I, the shown slope of 0 in the consecutive phases represents no change from the preactivation phase endpoint. For the entire figure: *N* = 3–5 independent mother-machine experiments inoculated from cultures obtained on separate days, details in Supplementary Table 4.

We next asked whether an already activated *agr-*system could be suppressed by heterologous AIPs, as reported in bulk population assays^12–14^. We focused on *agr*-types I and IV, which differ by a single amino acid in their AIPs and have been described as cross-activating^12–14^. Using mother-machine microfluidics, we preactivated bacteria with their homologous AIP for 3.5 h, then switched to a mixture of homologous and heterologous AIPs for 4 h and finally returned to homologous AIP alone for 4.5 h (Fig. 4a).

Contrary to results from bulk studies, all heterologous AIPs, including AIP-IV, which was expected to cross-activate, suppressed the *agr-*system of NM-I during mixed exposure. Moreover, no significant differences in inhibition strength were observed among the three AIPs (average lowest *agr*-ON fractions of 7%, 12%, and 23% for AIP-II, AIP-III, and AIP-IV, respectively; Fig. 4b, c; example movies in Supplementary Data 3). Similar inhibition patterns were observed in the native LAC-I strain (Supplementary Fig. 8a, b, example movies in Supplementary Data 4). For NM-IV, the heterologous AIP-II and AIP-III strongly suppressed the type-IV *agr*-system, whereas AIP-I did not. This was reflected by significant differences in inhibition strength, with average minimal *agr*-ON fractions of 91%, 5%, and 15% for AIP-I, -II, and -III, respectively (Fig. 4b, c). Thus, NM-I and NM-IV differed in their susceptibility to heterologous AIP inhibition despite near-identical AIP sequences, revealing asymmetry in cross-type interactions.

A*gr-*inhibition occurred rapidly, with the rate of decline in a*gr-*ON cells during mixed AIP exposure comparable to the activation rate during preactivation (Fig. 4d). Reactivation upon returning to homologous AIP began immediately but was slower than initial activation for NM-I (preactivation: 22% h^−^^1^, recovery: 6.3% h^−^^1^) but similar for NM-IV (preactivation: 20% h⁻¹, recovery: 15% h⁻¹, Fig. 4b–d). These kinetics highlight that while activation and inhibition proceed at similar speeds, recovery dynamics differ between *agr*-types.

Increasing the concentration of the heterologous AIP led to a similar overall pattern, with NM-IV remaining uninhibited by AIP-I and NM-I being inhibited by all tested heterologous AIP. However, for NM-I, the difference in inhibition strength between AIP-IV and AIP-II/AIP-III increased, potentially indicating incomplete inhibition by AIP-IV (Supplementary Fig. 9).

Our single-cell measurements show that heterologous AIPs can inhibit *agr*-activation despite constant homologous AIP supply, revealing *agr*-type specific inhibition. These effects may be less apparent in bulk assays, where AIP accumulation and population averaging can mask inhibitory dynamics. Thus, our microfluidic approach uncovers interaction patterns that depend on precisely controlled signaling environments and the ability to quantify *agr*-activation at the single-level, enabling discovery of partial inhibition.

### Spatially segregated co-cultures revealed distinct *agr* interaction regimes

**Fig. 5:**
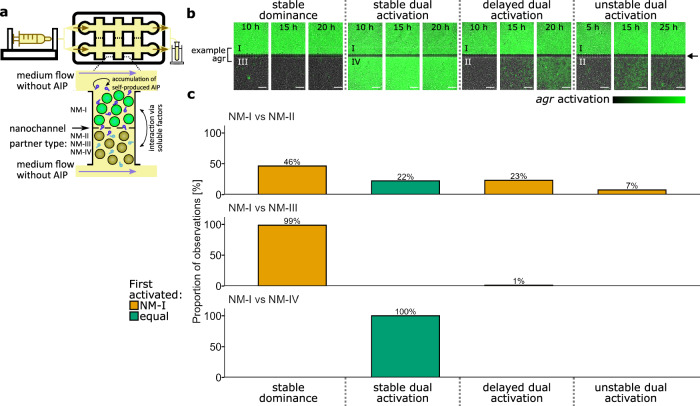
Diffusion-linked pairwise co-cultures of *agr*-types exhibit distinct interaction classes. **a** Schematic of connected-chamber microfluidic devices (chambers 50 × 50 µm). Growth medium without synthetic AIP was supplied independently to the top and bottom channels (20 × 100 µm) at 0.25 ml h^−1^. Nanochannels (0.4 µm gap) between chambers prevented cell mixing while permitting diffusion of soluble factors (including AIPs). NM-I was co-cultured with either NM-II, NM-III, or NM-IV. Each experiment was performed four times, encompassing up to 35 chamber pairs per replicate and each connected-chamber side sustaining communities of 1982 ± 271 cells. **b** Representative image sequence showing P3-*gfp* fluorescence at selected times post-inoculation (phase contrast overlaid with log_10_-transformed GFP; contrast adjusted for visualization). An arrow marks nanochannels; the two *agr-*types (representative example) are indicated on either side. Scale bar, 10 µm. **c** Distribution of interaction classes for each strain pairing (one row per pairing, summing to 100%): stable-dominance (one population constantly *agr-*ON, the other suppressed), stable-dual-activation (both constantly *agr-*ON with minor temporal differences), delayed-dual-activation (one population constantly *agr*-ON while the second population activated after a lag), and unstable-dual-activation (one population constantly *agr*-ON while the second population displayed fluctuating activation). Proportion is annotated. The *agr*-type that consistently or initially dominated is indicated by color (NM-I (orange) in all cases apart from equal (green) in stable-dual-activation). Connected-chamber pairs from all four biological replicates were combined. One chamber pair in the combination with NM-II was excluded due to difficulties categorizing it. Counts per class are listed in Supplementary Table 6.

Having shown that heterologous AIPs suppress preactivated *agr*-systems at single-cell resolution, we next asked how *agr-*interactions unfold between growing bacterial communities producing and sensing their own AIPs. We developed connected-chamber microfluidic devices in which adjacent populations grew in 50 × 50 µm, 0.85 µm-high chambers connected by 0.4 µm wide, 3 µm long nanochannels (Fig. 5a). Each chamber was supplied by its own 20 µm-high flow channel perfused at 0.25 ml h^−1^ by growth medium without synthetic AIP, creating advection in the flow channels and diffusion-limited exchange into the shallow chambers. This geometry prevents nutrient competition and cell translocation while permitting accumulation and diffusion of self-produced AIPs, enabling *agr*-crosstalk. Consistently, autoinduction occurred when the same congenic *agr-*strain was placed on both sides of the nanochannel-barrier for all four *agr*-types (Supplementary Fig. 10).

We co-cultured NM-I with either NM-II, NM-III or NM-IV and classified the temporal *agr-*interactions between the strains, focusing on the 5–25 h timeframe after inoculation. We observed four recurrent interaction classes (Fig. 5b and Supplementary Fig. 11, example movies in Supplementary Data 5): stable-dominance (one population constantly *agr*-ON while the other was suppressed), stable-dual-activation (both constantly *agr*-ON), delayed-dual-activation (one population constantly *agr-*ON while the second population activated delayed), and unstable-dual-activation (one population constantly *agr*-ON while the second population displayed fluctuating *agr*-activation). Class frequencies, combining all observed chamber-pairs from four replicates, depended on the strain pairing (Fig. 5c).

NM-I/NM-II co-cultures exhibited most prevalently (46%) stable-dominance of NM-I over NM-II, with rarer occurrences of all other classes. NM-I/NM-III co-cultures showed 99% stable-dominance of NM-I, while NM-I/NM-IV co-cultures showed 100% stable-dual-activation. Activation changes generally started near the nanochannels, illustrating an AIP diffusion-driven *agr-*activation among bacterial cells within the chambers (Supplementary Fig. 12).

When we repeated similar experiments with native-*agr* strains, we observed more diverse interaction patterns, characterized by two additional classes and a greater number of classes per *agr*-type pairing (Supplementary Fig. 13, example movies in Supplementary Data 6), highlighting a strong effect of genetic background on community-level signaling. The two additional classes are switched-dominance (the *agr*-ON population switches during observation) and initial-dominance (one population *agr*-ON early and both switching to *agr*-OFF later). LAC-I/502a-II pairings showed up to five different classes, with delayed dual activation being most prevalent (52%). LAC-I/MW2-III exhibited all six classes with stable-dominance of LAC-I being most prevalent (68%) and high occurrence of switched-dominance *agr*-ON from LAC-I to MW-III (18%). All six classes were observed for LAC-I/MNTG-IV pairings, with stable-dual-activation the most common (46%), followed by stable-dominance of MNTG-IV (24%). This stable-dominance outcome, absent in congenic I/IV pairings, showed that genetic background introduced variation into *agr-*interactions, leading in this case towards effective suppression of *agr-*I by AIP-IV, like observed with synthetic AIP mother-machine experiments.

We quantified early and late community descriptors to assess whether simple population features could explain the observed interaction classes beyond involved *agr*-types. Early community characteristics, such as initial cell numbers, early *agr*-activation, as well as chamber-wide cell density and cell-number fluctuations, carried little explanatory power in congenic strains but partly explained the interaction outcomes in native strains (Supplementary Fig. 14a–d).

These spatially segregated co-cultures showed that diffusional crosstalk organized community-level *agr*-activation, yielding dominance or dual-activation regimes depending on *agr-*pairing and genetic backgrounds. The initiation of *agr*-activation near nanochannels supported a diffusion-driven interaction model, while the divergence between congenic and native *agr-*strains underscored the importance of the genetic background in *agr* research. Together with our single-cell inhibition assays, these results illustrate that heterologous AIPs can readily interfere with *agr-*signaling at the community scale.

## Discussion

Using chemically controlled microfluidic environments and single-cell microscopy, we quantified *agr*-activation dynamics across all four *S. aureus agr*-types in congenic and native *agr*-type strains. These measurements revealed *agr-*type-specific differences in AIP sensitivity: *agr*-III consistently showed the lowest AIP sensitivity, whereas *agr*-IV was highly sensitive and exhibited elevated basal *agr*-activation without AIP stimulation. The observed bimodal *agr*-activation likely arose from subpopulations of cells that either remain *agr*-OFF or undergo rare ON → OFF switching despite continuous AIP supply. Functionally, heterologous AIPs inhibited *agr* in type-specific ways, including the unexpected partial inhibition of *agr*-I by AIP-IV. Finally, interactions between neighboring populations formed distinct classes with outcomes largely *agr*-type-driven in congenic pairings but more diverse and influenced by early population characteristics in native strains.

In both congenic and native *S. aureus* strains, *agr*-III was unresponsive to homologous AIP and *agr*-IV the most responsive *agr*-type, consistent with earlier batch‑culture findings^12,13,25^. The *agr*-III system remained functional, as shown by delayed but robust autoinduction in plate-reader assays and in connected-chamber experiments with *agr*-III on both sides. In these bulk contexts, *agr*-III activation occurred later than in other *agr*-types, consistent with our mother-machine observation that only the highest tested synthetic AIP concentrations triggered a small *agr-*ON fraction. This suggests that *agr-*III has a substantially higher AIP activation threshold. Current limitations in AIP quantification^26,27^ prevent confirmation of this hypothesis in bulk cultures. Differences in AIP maturation or export^28,29^ as alternative explanations are unlikely, as our use of exogenous synthetic AIP in microfluidics indicates that reduced sensitivity is not due to impaired AIP processing. Consequently, *agr*-III’s low sensitivity shifts the timing of *agr*-dependent virulence‑factor expression. These delayed kinetics may also reflect evolutionary optimization: *agr*‑III strains frequently harbor potent toxins, such as TSST-1^30^, expression of which is tightly governed by *agr* via the RNAIII/Rot regulatory axis^31^, a configuration that plausibly favors restrained or delayed activation to limit host damage while maintaining opportunities for transmission.

Both congenic and native *agr*-IV strains showed elevated basal *agr*-activation without AIP stimulation, potentially explained by stochastic promoter activation^32,33^. Phenotypic heterogeneity of *agr*-state likely confers a competitive advantage by accelerating population-level *agr-*responses when environmental conditions change^34^. The high AIP sensitivity and fast activation kinetics of *agr*‑IV directly support this interpretation, and the strong virulence associated with *agr*-IV backgrounds^11,35^ underscores its biological relevance. Moreover, exfoliative toxins, which are frequently carried in *agr*-IV/CC121 lineages^36,37^ and are regulated by *agr*^38^, may benefit from this rapid activation, as efficient expression of exfoliative activity is essential for disease and transmission. In contrast to the delayed and moderated activation of *agr*-III, the *agr*-IV architecture appears tuned for rapid, high-sensitivity *agr*-activation, suggesting fundamentally different QS strategies within the same species.

Independent qPCR measurements from early exponential phase cultures supported this activation hierarchy: across all strains, AIP stimulation increased expression of *agrA*, RNAIII, and the *agr*-regulated *psmβ2*. NM-III showed the lowest expression of all three genes under both conditions, consistent with its high activation threshold. NM-IV exhibited the highest basal and induced expression and was the only strain with consistently detectable RNAIII even without AIP stimulation. These transcriptional profiles corroborate the single-cell activation differences observed between *agr*-types. While these results confirm *agr*-type-specific activation dynamics in congenic strains, native strains revealed additional physiological consequences of AIP sensing.

In native strains, *agr*-type differences extended beyond AIP signaling to growth. The generation time of 502a-II increased markedly upon AIP stimulation. Because AIP accumulation typically correlates with nutrient limitation (in dense bulk populations), slower growth could represent an adaptive response to stress that is triggered by QS cues rather than by nutrient sensing itself^39–41^. Why this pronounced response emerged only in 502a-II remains unclear, but the absence of any comparable fitness cost in congenic strains, including NM-II, highlights the importance of the genetic background on *agr-*signaling interactions with cellular physiology.

Taken together, the *agr*-type-specific activation patterns and these additional physiological responses highlight that *S. aureus agr*-activation is shaped by a combination of signaling architecture and genetic-background‑specific context.

Our single-cell analysis revealed pronounced bimodal *agr*-activation, resulting in stable coexistence of *agr*-ON and *agr*-OFF bacteria, most prominently at intermediate AIP concentrations. While *agr*-OFF cells were clearly defined, the *agr*-ON peak showed more variability, with its fluorescence intensity depending on both AIP concentration and *agr*-type. This continuum within the *agr*-ON population indicates further heterogeneity among activated cells, which should be explored further. Overall, our data demonstrate that *agr-*bimodality arose from the intrinsic regulatory architecture of the QS system, rather than from spatial gradients, nutrient depletion, or other confounding bulk-culture effects^19,20^.

Mechanistically, the bimodal activation emerged from coexisting cell states: a major population that remained constantly *agr*-ON, indicating stabilization of the early OFF-to-ON switched bacteria, a small subpopulation of constant *agr*-OFF cells, and a rare but detectable class of ON-to-OFF switching cells contributing to the maintenance of the *agr*-OFF state. These subpopulations preserved a stable ON/OFF ratio under constant AIP exposure, indicating that *agr-*heterogeneity is dynamically maintained rather than a transient artifact. Importantly, the low frequency of switching events and their minimal contribution to total fluorescence would be entirely masked in bulk measurements.

Similar bimodal QS behaviors have been described in several bacterial systems^19,42,43^, reinforcing that heterogeneous QS activation is a widespread regulatory strategy rather than an anomaly of *agr*. In this context, *agr-*bimodality likely expands the phenotypic repertoire of *S. aureus* populations, enabling the simultaneous existence of virulence-competent cells and biofilm-enhanced subpopulations^10^. Such diversification aligns with bet-hedging strategies observed in bacteria, where maintaining subpopulations with differing activation thresholds improves survival in fluctuating environments, including host niches where QS cues vary^44,45^. Building on these intrinsic dynamics, we additionally investigated how *agr*-types influence one another when exposed to heterologous AIPs or competing strains.

AIP‑I fully activated *agr*‑IV, consistent with previous reports^14,25^, whereas AIP‑IV partially inhibited *agr*‑I, contrary to these earlier findings. Three factors are likely to contribute to this discrepancy. First, our use of microfluidics eliminated confounding effects from spent media, such as nutrient depletion or protease secretion^9^. Second, even subtle AIP sequence variations allow AgrC binding but prevent full activation, producing competitive inhibition or QS quenching^25,46^. Since *agr*-IV likely evolved from *agr*-I^16^, AIP-I may retain enough similarity to activate *agr*-IV, while AIP-IV binds but fails to fully activate *agr*-I, acting as a partial inhibitor. Third, single-cell resolution and constant AIP exposure enabled detection of partial inhibition that would be obscured by population averaging in bulk assays.

Beyond controlled synthetic AIP conditions, we quantified direct population interactions where strains produced their own AIPs and metabolites. Four recurrent interaction patterns emerged in congenic pairings, while native strains showed two additional classes. These outcomes reflected intrinsic signaling properties revealed by our mother‑machine assays. For example, NM‑III was always inhibited by NM‑I, consistent with NM‑I’s rapid activation, NM‑III’s higher AIP threshold, and known *agr*‑III inhibition by AIP-I^14,25^. NM-I and II showed comparable single-cell *agr*-activation kinetics, therefore leading to more stochastic interaction outcomes. In native strains, occasional dominance switch from LAC‑I to MW2‑III indicated that once activated, MW2‑III can maintain *agr*-activation and suppress LAC‑I, illustrating the importance of genetic background for interaction studies. While congenic I/IV co‑cultures showed dual-activation exclusively, matching previous cross-activation reports^12^, native MNTG‑IV could dominate LAC‑I, demonstrating population‑level inhibition of *agr*‑I by AIP‑IV in agreement with our mother-machine assays. Overall, native strain interactions were more variable and stochastic, whereas congenic strains behaved predictably. This determinism likely reflects intrinsic AgrC-AIP properties: for example, AgrC‑III is strongly inhibited by heterologous AIPs (IC₅₀ ≈1–3 nM), whereas AgrC‑I is inhibited only at higher concentrations (≈40–70 nM)^25^. Of note, our interaction studies focused on intraspecies pairings; interactions with cocolonizing staphylococci, known to modulate or inhibit *agr*^14^, or pathogens, such as *Pseudomonas aeruginosa*, remain important future directions.

Given these diverse interaction outcomes and the technical sensitivity required to resolve them, several limitations should be considered. Our *agr*‑P3 reporter was plasmid‑based rather than chromosomally integrated, and plasmids can impose metabolic burdens or induce compensatory mutations^47–49^, potentially affecting *agr*-activation thresholds and heterogeneity. In addition, both used *agr*-P3 reporters are multicopy plasmids, which may alter the stoichiometry between available promoter sites and phosphorylated AgrA. Because plasmid copy number varies between cells, this may also contribute to fluorescence variability within the *agr*‑ON population, which we omitted to analyze further. While the new plasmids eliminate the P2 promoter and thus avoid the AgrA sequestration inherent to the reporter used in native strains, the multicopy context still does not fully mirror the natural single-copy P2-P3 architecture. Nevertheless, strains showed stable generation times under AIP exposure (except for 502a‑II and NM‑III, which displayed inverse responses), and reproducible results across biological replicates and reporter plasmid constructs support reporter stability. Importantly, independent qPCR measurements in exponential‑phase cultures confirmed that *gfp* transcript levels strongly correlated with expression of *agrA*, RNAIII, and the *agr*‑regulated *psmβ*2 under AIP stimulation, demonstrating that the P3‑*gfp* reporter reliably reflects chromosomal *agr*-activation despite its multicopy nature. Furthermore, non‑fluorescent cells frequently produced fluorescent offspring, indicating neither plasmid loss nor disruptive mutations. Finally, while the observation of ON-to-OFF switching events and rapid GFP decline upon AIP inhibition suggests minimal temporal delay in our system, GFP maturation and stability may still introduce bias regarding *agr*-activation‑state persistence^50,51^.

At the clinical level, correlations between *agr*‑type and disease severity remain inconsistent, likely due to geographic and lineage‑specific effects^15,52–54^. Integrating single‑cell *agr-*phenotyping of clinical isolates, including AIP sensitivity, heterologous inhibition, and *agr*-bimodality, may help link QS behavior to colonization dynamics and infection outcomes.

Methodologically, the mother-machine and connected‑chamber setup and image analysis pipeline provide a scalable framework to quantify candidate QS inhibitors^55,56^ and dissect interspecies crosstalk at single-cell resolution. Biologically, the distinct AIP sensitivity hierarchies and *agr-*interaction patterns we identified emphasize meaningful differences between *agr*-types beyond genetic background, underscoring that inhibition efficacy may vary across *agr*-types and should be evaluated carefully when developing broad‑spectrum anti‑virulence strategies^38^.

## Methods

### Bacterial strains and growth conditions

Strains used in this study are listed in Table 1. Congenic Newman strains (abbreviated as NM-I, NM-II, NM-III, and NM-IV) were provided by Bo Shopsin and originate from Geisinger et al.^13^. The original authors replaced the native *agr-*locus with one of the four *agr*-types isolated from native-*agr* strains. Whole-genome sequencing revealed only a small number of mutations, likely introduced during deletion or insertion of the *agr*-system (polymorphisms listed in Supplementary Table 8).

**Table 1:** Strains and plasmids used in this study

*S. aureus* strains					
Strain	*agr*-type	Origin of the *agr* sequence	ST type	Plasmid	Reference
NM-I (EG802)	I	NTCT8325-4	ST8	None	Geisinger et al.^13^
NM-II (EG803)	II	RN6607 (502a)	ST8	None	Geisinger et al.^13^
NM-III (EG804)	III	RN3984	ST8	None	Geisinger et al.^13^
NM-IV (EG805)	IV	RN4850	ST8	None	Geisinger et al.^13^
NM-I	I	NTCT8325-4	ST8	pALC1484-P3seq2-GFP	This study
NM-II	II	RN6607 (502a)	ST8	pALC1484-P3seq2-GFP	This study
NM-III	III	RN3984	ST8	pALC1484-P3seq4-GFP	This study
NM-IV	IV	RN4850	ST8	pALC1484-P3seq3-GFP	This study
LAC	I	Native	ST8	pDB59	Yarwood et al.^7^
502a	II	Native	ST5	pDB59	Yarwood et al.^7^
MW2	III	Native	ST1	pDB59	Yarwood et al.^7^
MNTG	IV	Native	ST2276	pDB59	Yarwood et al.^7^
*E. coli* strains					
Strain	Description				Reference
DC10B	*dam*^*+*^ Δ*dcm* Δ*hsdRMS endA1 recA1*				Monk et al.^75^
IM08B	SA08BΩP_N25_-*hsdS* (CC8-1) (SAUSA300_0406) of NRS384 integrated between the *essQ* and *cspB* genes				Monk et al.^76^
Plasmids					
Plasmid name	Backbone	Antibiotic marker	Reporter	P3 origin	Reference
pALC1484	pALC1484	Cm10	GFP	None	Kahl et al.^57^
pALC1484-P3seq2-GFP	pALC1484	Cm10	GFP	MW2	This study
pALC1484-P3seq3-GFP	pALC1484	Cm10	GFP	RN4850	This study
pALC1484-P3seq4-GFP	pALC1484	Cm10	GFP	EG804	This study
pDB59	pUC18/pC194	Cm10	YFP	MN8	Yarwood et al.^7^

For the Newman (NM) strains, the originally listed strain identifier and *agr*-locus origin used to complement the Newman *agr-*knockout strain is indicated. These strains were transformed with P3-*gfp* reporter plasmids with perfectly matching P3 promoter sequence. The corresponding sequence can be found in Supplementary Table 9. All native-*agr* strains have been transformed with the pDB59 plasmid.

Additional *S. aureus* strains (LAC: *agr-*I; 502a: *agr-*II; MW2: *agr-*III; MNTG: *agr-*IV) carrying the pDB59 P3-*yfp* reporter plasmid (plasmid originally from Yarwood et al.^7^) were provided by Alexander Horswill.

For overnight cultures, single colonies were inoculated into 3 ml tryptic soy broth (TSB; BD) supplemented with 10 µg ml^−^¹ chloramphenicol (Cm10) for plasmid maintenance and 0.01% Tween20 to minimize aggregation and biofilm formation for any microscopy experiments. Cultures were incubated at 37 °C with shaking at 220 rpm.

Synthetic AIPs were purchased from Cambridge Research Biochemicals (now operating as BIOSYNTH, catalog codes CRB1000249, CRB1001687, CRB1001688 and CRB1001689 for AIP-I, -II, -III and -IV, respectively). Stock solutions were prepared in DMSO and stored at −20 °C. Before use, AIPs were diluted in phosphate-buffered saline (PBS) and added to growth medium at the indicated concentrations. DMSO concentrations were kept below thresholds affecting growth or *agr-*signaling. Control samples received 2.5% PBS instead of AIP, matching the buffer concentration used for AIP dilution.

### P3-*gfp* plasmid construction and bacterial transformation

We constructed perfectly matching P3 reporter plasmids for the four congenic Newman strains (Table 1). The respective P3 promoters of each *agr*-type were amplified using Taq Phusion (Thermo). The oligonucleotides (obtained from Microsynth Switzerland) and amplified genomic DNAs (gDNA) from the strains are listed in the Supplementary Table 9. In our set of strains, NM-I, NM-II and MW2 share the same P3 sequence, NM-III has a unique P3 sequence, and NM-IV and MNTG share the same P3 sequence. The amplicons were fused to the *gfpuv* gene by ligation into the pALC1484 plasmid^57^ using the appropriate restriction enzymes (Supplementary Table 9). All constructs were verified by PCR and sequencing. Chemically competent *Escherichia coli* DC10B or IM08B were transformed with the plasmids before transformation by electroporation into the respective Newman strains. Of note, our constructed P3-*gfp* plasmids only contain the P3 promoter, while the pDB59 carries both P2 and P3 promoters and was optimized for the MN8 strain^7^ which may impact AgrA binding efficiency and increase the required phosphorylated AgrA for P3 activation.

### Plate-reader-based *agr*-activation

Two-fold serial dilution (25 µl) of autoinducing peptide (AIP) in PBS was performed. Bacteria were then inoculated at on OD_600 nm_ of 0.0025 in TSB containing 10 µg ml^−1^ chloramphenicol, derived by washing twice an overnight culture in PBS and then diluted in PBS to an OD_600 nm_ of 0.5. Absorbance at 600 nm and fluorescence (GFP excitation: 475 nm, emission: 525 nm; YFP excitation: 500, emission: 540 nm) were measured with an iD3 plate reader (Molecular Devices) in 15 min intervals over 48 h with intermittent shaking. *Agr*-activation start was calculated as the first time a GFP measurement exceeded the threshold of 10^6^ a.u.

### Mother-machine microfluidic device

Microfluidic device molds were provided by Martin Ackermann; design and fabrication methods have been published^58^. In summary, the device contained six independent replicate channels, each containing approximately 4000 dead-end chambers (height: 0.93 µm; length: 25 µm; width: 1.2–1.6 µm) arranged perpendicular to a main flow channel (height: 21 µm; width: 200 µm). For fabrication, polydimethylsiloxane (PDMS; Sylgard 184 Silicone Elastomer Kit, Dow Corning) was mixed with curing agent at a 10:1 ratio, poured onto the mold, degassed for 10 min, and cured at 80 °C for 1 h. Devices were cut, inlet/outlet holes were punched (0.75 mm), and plasma-bonded to #1.5 glass slides (Corning).

A total flow of 0.5 ml h^−1^ entered the device and split into two parallel 21 µm-high, 200 µm-wide main channels, resulting in a mean flow velocity of ~1.6 × 10⁴ µm s^−1^ (*v* = *Q*/*A*). Each main channel independently supplied its corresponding set of mother-machine chambers. Because the chambers are only 0.93 µm high, the height mismatch prevents advection; solutes enter chambers exclusively by diffusion. Accordingly, the boundary condition at each chamber entrance is always a fresh medium at full AIP concentration whenever AIP was supplied. Diffusion across the 25 µm chamber length occurs within seconds (~1–6 s for metabolites and short peptides^59,60^), ensuring a spatially uniform chemical environment. Accordingly, GFP fluorescence profiles were homogeneous across chamber length (Supplementary Fig. 15; slope of log₁₀(intensity + 1) per chamber length [µm] = −0.0045 log_10_(AU) µm^−1^).

### Connected-chamber microfluidic device

Device molds were fabricated by SU‑8 lithography on silicon wafers. The design incorporated two 20 µm-high, 100 µm-wide flow channels, each feeding a grid of 100 chamber pairs (each chamber: height 0.85 µm; 50 µm × 50 µm). Each pair was separated by 0.4 µm-wide, 3 µm-long nanochannels, allowing solute diffusion but preventing cell translocation. This configuration enables co‑culturing of different strains in adjacent chambers with hundreds of parallel replicates. For fabrication, PDMS was mixed at a 5:1 ratio, poured onto molds, degassed 20 min, and cured at 80 °C for 2 h. Devices were cut, heated (120 °C, 20 min), inlet/outlets punched (1.5 mm), rinsed with isopropanol, and plasma-bonded to #1.5 glass slides (VWR). Changes in *agr*-activation typically originated near nanochannels and propagated across the chambers (Supplementary Fig. 12, Supplementary Data 5 and 6).

Each 20 µm-high, 100 µm-wide flow channel was perfused at 0.25 ml h^−1^, corresponding to a mean flow velocity of ~3.5 × 10⁴ µm s^−1^ (*v* = *Q*/*A*). Advection occurred only in these tall flow channels; solute entry into the shallow 0.85 µm-high chambers was diffusion-limited. Because each chamber is supplied by its own independent flow channel, no nutrient competition occurs between the two interacting populations. The diffusion‑dominated regime supports AIP accumulation inside chambers, while nanochannels mediate AIP‑driven QS crosstalk.

### Microfluidic device inoculation

Overnight cultures were diluted 1:50 in 4 ml of fresh medium (TSB + Cm10 for plasmid maintenance + 0.01% Tween20 to reduce aggregation) and incubated for 2 h. Cells were then washed twice with TSB + Cm10 + 0.1% Tween20 and concentrated to 80 µl. The dense suspension was loaded into devices from the outlet side using a pipette. The outlet and inlets were connected to a waste reservoir and a syringe pump (KF Technology, NE 1600), respectively. PTFE tubing (inner diameter: 0.3 mm; outer diameter: 0.76 mm; Adtech) was used for mother‑machine devices, and medical-grade Tygon tubing (inner diameter: 0.51 mm; outer diameter: 1.52 mm) for connected‑chamber chips.

For mother‑machine experiments, TSB + Cm10 + 0.01% Tween20 was used as growth medium, and cells were grown overnight in the device after loading to allow metabolic recovery and complete *agr*-deactivation before AIP exposure. In contrast, imaging of connected‑chamber chips began immediately after loading to capture community growth from inoculation. 1:1 ratio of TSB and PBS + Cm10 + 0.01% Tween20 was used for connected-chamber experiments. PBS was added to reduce division rates slightly. All experiments were conducted at 37 °C.

### Image acquisition

We used a fully automated Olympus IX83 P2ZF inverted microscope, controlled via CellSense 3.2 (Olympus), for imaging. Phase contrast (200 ms exposure, 130 intensity IX3 LED) and GFP fluorescence for congenic-*agr* strains (200 ms exposure, 20% intensity 490 nm CoolLED pE4000, quad-band filter for DAPI/GFP/Cy3/Cy5) or YFP fluorescence for native-*agr* strains (200 ms exposure, 20% intensity 500 nm CoolLED pE-4000, triple-band filter for CFP/YFP/mCherry) images were captured at 16-bit resolution in intervals ranging from 2 to 15 min using a 100×, 1.3 numerical aperture oil phase objective (Olympus) and an ORCA-flash 4.0 sCMOS camera (Hamamatsu). Up to 16 positions for each of the six replicates of mother-machine devices per chip and up to 36 positions per replicate on connected-chamber chips were manually selected.

### Image preprocessing

Pre-processing steps were executed in MATLAB (MathWorks) on the phase contrast images and differ slightly for mother-machine devices and connected-chamber chips (Supplementary Fig. 16). For mother-machine devices, the image region containing the physical position labeling was detected by a multi-stage binarization algorithm and cropped out to provide a non-changing region for image registration. On these, multimodal estimation of the geometric transformation for translation was performed to align consecutive frames to the first with a one-plus-one evolutionary optimization algorithm. The position of chambers was detected by a sequence of filtering and binarization steps on the first frame. Filled chambers on the first frame were determined with a circular Hough transformation for circle detection. Only these first frame-filled chambers (for all consecutive frames) were cropped out and copied into separate folders for further processing. The process is illustrated in Supplementary Fig. 16.

Connected-chamber chip registration was performed using the same translation estimation method, but on the entire downscaled and filtered image. Chamber detection was performed by detecting peaks of high intensity on the filtered image along the x and y coordinates, which correspond to the border of the chambers.

The computed image translation was applied to fluorescent images as well. Beforehand, fluorescent 16-bit images were normalized using [0.005, 0.75] • 2^16^ or [0.002, 0.85] • 2^16^, for mother-machine or connected chamber chips, respectively.

Positions affected by registration errors, chamber detection issues, frequent out-of-focus frames, or obvious cell death (likely due to pressure changes during media switches) were manually excluded after reviewing overview movies generated in MATLAB.

### Image analysis: cell segmentation and tracking

Single-cell segmentation was carried out using StarDist (v0.8.3)^23^ and tracking with a modified version of DeLTA (v2.0)^24^, both implemented in Python (v3.7 and v3.9, respectively). For mother-machine experiments, all cells within the lower half of the chamber were segmented. For connected-chamber experiments, a small border at the flow-channel opening and a safety margin around the nano-channels were excluded to ensure accurate chamber assignment of cells.

Due to the pre-trained StarDist models sub-optimally performance, we trained two custom segmentation models tailored to the distinct microfluidic designs. Differences in cell density and image complexity between mother-machine and connected-chamber chips necessitated this approach. We generated 707 and 508 training images for mother-machine (one chamber per image) and connected-chamber chips (random 100 × 100 px crops), respectively (Supplementary Fig. 17a–d). Annotation was performed in FIJI using LABKIT^61^. A preliminary model trained on 100 annotated images was iteratively refined through prediction-correction cycles until the full dataset was completed. Final training started from a blank model, and parameters included: 64 rays, 64 × 64 px patches, validation split 0.125, batch size 8, 400 epochs, 100 steps per epoch, and data augmentation (random flips, rotations, intensity changes, and noise addition). Only the best-performing model (lowest validation loss) was retained. Segmentation precision was satisfactory for both designs (Supplementary Fig. 17e, f). Cell properties, including fluorescence intensity reported here as *agr*-activation, were quantified using regionprops from scikit-image (v0.19.3)^62^. Pixel area from regionprops was transformed to µm^2^ using the spatial calibration factor (0.065) retrieved from the microscope. For mother-machine experiments, cells were filtered by size (area: 0.3–2.5 µm²). Response-time analysis included only replicates reaching ≥50% activated cells. For connected-chamber experiments, only chambers with a median cell count ≥700 were analyzed.

Tracking in connected-chamber chips was not feasible due to high cell density and limited image acquisition rates. Existing pipelines for mother-machine cell tracking, including DeLTA 2.0, were optimized for rod-shaped bacteria and performed poorly on cocci. To address this, we generated 11,671 new cocci-specific tracking event sets for mother-machine devices using scripts provided by Owen M. O’Connor^24^ and combined them with 4732 randomly selected sets from the original dataset. We modified the original weight function for a more uniform distribution and retrained the deep-learning model with the following parameters: validation split 0.125, batch size 8, 900 epochs, 1500 steps per epoch (200 for validation), and early stopping (patience: 30 epochs). Default DeLTA augmentation was applied, and only the best model (lowest validation loss) was retained.

We adapted DeLTA 2.0 to use StarDist2D segmentations instead of its built-in segmentation algorithm for improved accuracy. Outputs, including lineage information and fluorescence intensities, were converted into Python pandas tables using code from Simon van Vliet^63^. For analysis, we included only cells with an observed mother cell and division event. The time between these division events was defined as the generation time. Cells with a generation time <10 min (assumed erroneous tracking) or >6 h (assumed dead) were excluded. The delayed division threshold of 60 min is the 93-quantile of control experiments, chosen to clearly separate normal and impaired division. Constant *agr*-ON, constant *agr*-OFF and switched *agr*-state were defined by the *agr*-activation of bacterial cells right after and before division. Intergenerational stability analysis required ≥6 lineages per *agr*-state per replicate. A lineage was defined as all offspring cells with observable division events during the AIP exposure phase, starting from the first recorded cell division event after AIP exposure start, up to a maximum of 10 generations after the first-dividing cell. The proportion of *agr*-state of offspring cells was calculated based on the average *agr*-activation of each individual cell within the lineage. The proportion of matching *agr*-state to the first dividing cell is reported.

Data presentation: Figs. 1, 4, and 5 show StarDist segmentation data; Figs. 1d depict raw single-cell data, while other panels present aggregated metrics. Figures 2 and 3 display DeLTA tracking data. Connected-chamber data were aggregated into descriptive statistics for 6.5 × 6.5 µm bins or entire chambers.

### Classifying temporal *agr-*interactions of the connected-chamber chips

Mean GFP or YFP signal per community was used to binarize *agr-*activation into ON and OFF states (>100 a.u.). The proportion of ON state within the experimental time window of 5–25 h, chosen because by then chambers were generally filled with bacteria and any carryover *agr-*activation was reset, was calculated and used for *agr-*interaction classification. Only frames with at least 30 cells per chamber have been considered, and only chambers with a median number of cells over time larger than 700 within the experimental time window were included. A*gr*-interactions have been classified regarding the proportion of time each of the two interacting communities is in the activated *agr-*state and the relative activity of these two.

Stable-dominance was defined as minimally 40% unequal ON state between the two interacting *agr-*types, maximally 10% simultaneous ON state and either of the communities being ON for at least 60% and the other being ON maximally for 15%.

Stable-dual-activation was defined as at least 40% simultaneous ON state and maximally 40% unequal ON state.

Delayed-dual-activation was defined as an intermediate proportion of unequal ON state among the interacting *agr-*types (>30%) and at least 10% of simultaneous ON state. Either one of the communities was required to be ON for at least 40% of the time, and the other more than 65%.

Unstable-dual-activation was defined as maximally 15% of simultaneous ON state, minimally 30% unequal ON state and one of the communities being ON for less than 60% and being ON already in the first 40% of observed time.

Initial-dominance was defined as both OFF minimally 30%, one maximally 20% ON and the other more than 20% ON and the last ON timepoint of it happening early during the experimental phase.

Switched-dominance was defined as at least 30% unequal ON state among the interacting *agr-*types and either of them being ON in the early phase of the experiment (first 40% of time observed) and either of the communities being ON for minimally 15% and maximally 80%.

One NM-I vs NM-II chamber pair failed to classify unambiguously and was excluded.

### Whole-genome sequencing and comparative genomics

DNA was isolated from bacterial colonies of the congenic-*agr* strains without the reporter plasmid grown on Columbia Sheep Blood agar plates (CSB, bioMérieux) and libraries prepared with the QIASeq FX DNA library kit (Qiagen) and sequenced with a MiSeq instrument using MiSeq reagent kit V2 (Illumina). 150 bp paired-end reads were assembled with SPAdes (v3.15.5^64^) with the “careful” argument and default k-mer size. Annotation was performed with prokka (v1.14.6^65^), and polymorphisms detected with snippy (v4.6.0^66^) using https://github.com/judithbergada/Pipeline_GenomeAnalysis.

### Exponential culture growth and RNA isolation for RNA sequencing and qPCR

We grew overnight cultures in TSB ± Cm10 at 37 °C, centrifuged them at 18.8 × *g* for 2 min, and washed them twice in PBS. Then, we diluted this culture 1:20 into fresh TSB ± Cm10, and regrew for 1.5 h. We repeated this wash-dilute-regrow cycle twice more to remove AIPs and reset *agr-*activation, and collected cells after 1.5 h of the third subculture. For qPCR, the last culture was performed twice in parallel, one without AIP stimulation and one with 256 nM homologous, synthetic AIP. We stored bacterial pellets of exponential cultures in RNAlater until RNA extraction. Total RNA was isolated using the RNeasy Plus Mini kit (Qiagen) following manufacturer instructions, with additionally adding lysostaphin for improved lysis (0.1 mg ml^−^¹, Biosynth) and followed by double DNase treatment (TURBO DNase, Invitrogen). RNA was stored at −80 °C until further usage.

### RNA-seq analysis

We used congenic-*agr* strains without the reporter plasmid for the cultures. The Functional Genomics Center Zurich (University of Zurich and ETH Zurich) prepared paired-end 150 bp RNA-seq libraries using the TruSeq RNA Library Prep Kit and sequenced them on an Illumina NovaSeq X Plus without rRNA depletion, yielding on average 35 million reads per sample. We processed data with a custom pipeline: quality control and filtering with fastp (v0.23.4^67^ and FastQC (v0.11.8 http://www.bioinformatics.babraham.ac.uk/projects/fastqc); rRNA removal using sortmerna (4.3.7, database v4.3.4^68^); alignment to the *S*. *aureus* Newman reference genome (RefSeq GCF_000010465.1) with bowtie2 (v2.5.2^69^); and read summarization with featureCounts (subread v2.0.8^70^). We imported counts into R and performed differential expression analysis using DESeq2 (2.0.8v^71^) and apeglm^72^ shrinkage, and enhanced genome annotation using AureoWiki pan-genome resources^73^.

### qPCR

We used congenic-*agr* strains with the reporter plasmids for the cultures. cDNA was produced using the GoScript Reverse Transcription System (Promega) following manufacturer instructions and using 10.5 µl of samples after normalizing RNA concentration. qPCR was performed on a CFX Connect System (BIO-RAD) with the primers listed in Supplementary Table 10 obtained from Microsynth Switzerland. Efficiency for each primer pair was calculated and utilized for relative expression calculation. Samples below the limit of detection (Cq = 30) are indicated in Fig. 1g.

Bacterial load as colony-forming units was quantified by serial dilution onto Columbia Sheep Blood agar plates (bioMérieux). The cultures were then transferred to a plate-reader (iD3, Molecular Devices) to record GFP fluorescence and optical density over 24 h growth at 37 °C with intermittent shaking (see above).

### Statistical analysis

Biological replicates were defined as independently started cultures grown on separate days. For mother‑machine experiments, all AIP concentrations were tested in parallel whenever possible by loading aliquots from the same culture into separate flow channels of the same microfluidic device. Each biological replicate, therefore, consisted of a new culture, with individual AIP conditions obtained from parallel channels loaded from that culture. For connected‑chamber experiments, each biological replicate was performed on a separate microfluidic device, with up to three devices imaged in parallel. Mixed‑effects models included the replicate identifier as a random effect to account for the non‑independence of AIP conditions derived from the same overnight culture. Plate-reader as well as RNAseq experiments have been performed on independent cultures started on separate days.

All statistical analyses and figure generation were performed in R (v4.5.0) using RStudio and the tidyverse package^74^. Response times to AIP concentration changes across biological replicates were assessed using linear regression with an interaction term for *agr-*type and log₂-transformed AIP concentration, followed by EMM *post-hoc* tests (Supplementary Figs. 3 and 4). Differential gene expression (RNA-seq) from exponential-phase cultures was analyzed using DESeq2^71^ and apeglm^72^ with default parameters (Supplementary Fig. 5). Log_10_ transformed relative expression (qPCR) was analyzed using a mixed-effects model with the interaction term for *agr*-type and AIP concentration (factor of 0 or 256 nM) and random intercept of replicate, followed by estimated marginal means (EMM) https://rvlenth.github.io/emmeans/*post-hoc* tests (Fig. 1g). Linear regression correlation of *gfp* with either of the other three transcripts was calculated and slope, R^2^ and *p*-value reported (Supplementary Fig. 6). The proportion of bacteria with prolonged generation time was analyzed using a generalized linear mixed-effects model (binomial family) including *agr*-type × AIP concentration interaction and a random intercept for replicate, followed by EMM *post-hoc* tests (Fig. 2B and Supplementary Fig. 7c). Log_10_ transformed generation time of control experiments was assessed with a mixed-effects model followed by EMM pairwise comparisons (Supplementary Fig. 7a, b). Cell area was assessed using a linear mixed-effects model with a two-way interaction between AIP concentration and generation-time cutoff (Supplementary Fig. 7d, e). The proportion of lineages switching *agr*-state was modeled using binomial regression with time and *agr*-type as interacting predictors (Fig. 2d, e, and Supplementary Fig. 7). Lowest proportions of activated bacteria across replicates were compared using Dunnett’s test (Fig. 4c, Supplementary Figs. 9b and 10b). Linear regression for each of the phases of heterologous AIP exposure experiments and strain and AIP combination was calculated, and the slope visualized in Fig. 4d, Supplementary Figs. 9c and 10c. Interaction dynamics in connected-chamber chips were analyzed using multinomial regression https://CRAN.R-project.org/package=nnet based on four predefined parameters (Supplementary Fig. 14). Correlation between cell position within chamber and *agr*-activation was assessed with linear regression per strain and AIP concentration (Supplementary Fig. 15).

### Declaration of generative AI and AI-assisted technologies in the writing process

The manuscript was written by the authors. Microsoft M365 Copilot (GPT-5.2, last accessed on 11th of February 2026) was employed for grammar correction, text streamlining and improving language clarity and as a tool to explore alternative phrasings. GitHub Copilot (Claude Sonnet 4.5, last accessed at 13th of February 2026) was used for code readability and efficiency improvements and streamlining. The authors have thoroughly reviewed, verified, and edited any passages generated by any LLM, taking full responsibility for the manuscript and overall quality and accuracy.

### Reporting summary

Further information on research design is available in the Nature Portfolio Reporting Summary linked to this article.

## Supplementary information

Supplementary Information

Description of Additional Supplementary Files

Supplementary Data 1

Supplementary Data 2

Supplementary Data 3

Supplementary Data 4

Supplementary Data 5

Supplementary Data 6

Supplementary Movie 1

Supplementary Movie 2

Reporting Summary

Transparent Peer Review File

## Data Availability

Example movies of one position for each experimental condition are shared as Supplementary Data 1–6 (compressed folders containing multiple labeled mp4 files). Raw image files of all example movies, including the output of the fully executed bioimage analysis pipeline, are deposited on BioImage Archive with the accession number S-BIAD2927 for congenic *agr*-strains and with the accession number S-BIAD1046 for native *agr*-strains. Additional raw images or processed data are available upon request and are not deposited online due to large file sizes. Retrained StarDist2D segmentation models for the mother-machine and connected-chamber designs, as well as the retrained DeLTA 2.0 tracking model and the used training data for these three models has been deposited on Zenodo 10694024. Raw DNA sequencing data of both the congenic and native strains are deposited in the European Nucleotide Archive (ENA) at EMBL-EBI under accession number PRJEB72799. The 1.5 h samples of the ENA RNA sequencing repository with accession number PRJEB107700 were used in this study; specific sample identifiers are listed in Supplementary Table 11. Tabular source data for all main and Supplementary Figs. are available in the Figshare repository 31338796.

## Code availability

Code for image processing is available here: 10.5281/zenodo.18623629. Whole genome sequencing analysis code is available here: https://github.com/judithbergada/Pipeline_GenomeAnalysis (version of commit fb858a0). RNAseq analysis pipeline is available here: 10.5281/zenodo.18492655.
