# Supplementary material for: Single-cell approach dissecting agr quorum sensing dynamics in Staphylococcus aureus
Source: Nat Commun. 2026 May 20;17:8139. doi: 10.1038/s41467-026-73552-9 (PMC13458059; doi:10.1038/s41467-026-73552-9)
Supplement: Supplementary file 1 [file 41467_2026_73552_MOESM11_ESM.pdf]

nature portfolio

Corresponding author(s): Annelies S. Zinkernagel

Last updated by author(s): Feb 14, 2026

Reporting Summary

Nature Portfolio wishes to improve the reproducibility of the work that we publish. This form provides structure for consistency and transparency in reporting. For further information on Nature Portfolio policies, see our Editorial Policies and the Editorial Policy Checklist.

Statistics

For all statistical analyses, confirm that the following items are present in the figure legend, table legend, main text, or Methods section.

n/a	Confirmed	
☐	☒	The exact sample size (*n*) for each experimental group/condition, given as a discrete number and unit of measurement
☐	☒	A statement on whether measurements were taken from distinct samples or whether the same sample was measured repeatedly
☐	☒	The statistical test(s) used AND whether they are one- or two-sided<br>*Only common tests should be described solely by name; describe more complex techniques in the Methods section.*
☐	☒	A description of all covariates tested
☐	☒	A description of any assumptions or corrections, such as tests of normality and adjustment for multiple comparisons
☐	☒	A full description of the statistical parameters including central tendency (e.g. means) or other basic estimates (e.g. regression coefficient) AND variation (e.g. standard deviation) or associated estimates of uncertainty (e.g. confidence intervals)
☐	☒	For null hypothesis testing, the test statistic (e.g. *F*, *t*, *r*) with confidence intervals, effect sizes, degrees of freedom and *P* value noted<br>*Give P values as exact values whenever suitable.*
☒	☐	For Bayesian analysis, information on the choice of priors and Markov chain Monte Carlo settings
☒	☐	For hierarchical and complex designs, identification of the appropriate level for tests and full reporting of outcomes
☒	☐	Estimates of effect sizes (e.g. Cohen's *d*, Pearson's *r*), indicating how they were calculated

Our web collection on statistics for biologists contains articles on many of the points above.

Software and code

Policy information about availability of computer code

Data collection	We used a fully automated Olympus IX83 P2ZF inverted microscope, controlled via CellSense 3.2 (Olympus), for imaging. qPCR was performed on a CFX Connect System (BIO-RAD). Plate-reader (iD3, Molecular Devices) to record GFP fluorescence and optical density was operated with the systems software.
Data analysis	Image analysis<br>We developed an image-analysis pipeline operating exclusively on phase-contrast images to maximize generalizability, enabling all fluorescence channels to be reserved for reporter quantification (here, GFP or YFP for P3 activation). Initial steps including image registration and chamber detection were performed in MATLAB (MathWorks, details in Supplementary Methods and Supplementary Fig.16). Positions affected by registration errors, chamber detection issues, frequent out-of-focus frames, or obvious cell death (likely due to pressure changes during media switches) were manually excluded after reviewing overview movies generated in MATLAB.<br>Single-cell segmentation was carried out using StarDist (v0.8.3)23 and tracking with a modified version of DeLTA (v2.0)24, both implemented in Python (v3.7 and v3.9, respectively). For mother-machine experiments, all cells within the lower half of the chamber were segmented. For connected-chamber experiments, a small border at the flow-channel opening and a safety margin around the nano-channels were excluded to ensure accurate chamber assignment of cells.<br>Due to the pre-trained StarDist models sub-optimally performance, we trained two custom segmentation models tailored to the distinct microfluidic designs. Differences in cell density and image complexity between mother-machine and connected-chamber chips necessitated this approach. We generated 707 and 508 training images for mother-machine (one chamber per image) and connected-chamber chips (random 100 × 100 px crops), respectively (Supplementary Fig. 17a-d). Annotation was performed in FIJI using LABKIT63. A preliminary model trained on 100 annotated images was iteratively refined through prediction-correction cycles until the full dataset was completed. Final training started from a blank model and parameters included: 64 rays, 64 × 64 px patches, validation split 0.125, batch size 8, 400 epochs, 100 steps per epoch, and data augmentation (random flips, rotations, intensity changes, and noise addition). Only the best-performing model

(lowest validation loss) was retained. Segmentation precision was satisfactory for both designs (Supplementary Fig.17e f). Cell properties, including fluorescence intensity reported here as agr-activation, were quantified using regionprops from scikit-image (v0.19.3)64. Pixel area from regionprops was transformed to µm2 using the spatial calibration factor (0.065) retrieved from the microscope. For mother-machine experiments, cells were filtered by size (area: 0.3-2.5 μm^2). Response-time analysis included only replicates reaching ≥50% activated cells. For connected-chamber experiments, only chambers with a median cell count ≥700 were analyzed.

Tracking in connected-chamber chips was not feasible due to high cell density and limited image acquisition rates. Existing pipelines for mother-machine cell tracking, including DeLTA 2.0, were optimized for rod-shaped bacteria and performed poorly on cocci. To address this, we generated 11,671 new cocci-specific tracking event sets for mother-machine devices using scripts provided by Owen M. O'Connor24 and combined them with 4,732 randomly selected sets from the original dataset. We modified the original weight function for a more uniform distribution and retrained the deep-learning model with the following parameters: validation split 0.125, batch size 8, 900 epochs, 1,500 steps per epoch (200 for validation), and early stopping (patience: 30 epochs). Default DeLTA augmentation was applied, and only the best model (lowest validation loss) was retained.

We adapted DeLTA 2.0 to use StarDist2D segmentations instead of its built-in segmentation algorithm for improved accuracy. Outputs, including lineage information and fluorescence intensities, were converted into Python pandas tables using code from Simon van Vliet65. For analysis, we included only cells with an observed mother cell and division event. The time between these division events was defined as generation time. Cells with generation time <10 min (assumed erroneous tracking) or >6 h (assumed dead) were excluded. Delayed division threshold of 60 min is the 93-quantile of control experiments, chosen to clearly separate normal and impaired division. Constant agr-ON, constant agr-OFF and switched agr-state were defined by the agr-activation of bacterial cells right after and before division. Intergenerational stability analysis required ≥6 lineages per agr-state per replicate. A lineage was defined as all offspring cells with observable division events during AIP exposure phase starting from the first recorded cell division event after AIP exposure start up to maximum of 10 generations after the first-dividing cell. The proportion of agr-state of offspring cells was calculated based on average agr-activation of each individual cell within the lineage. Proportion of matching agr-state to the first dividing cell is reported.

Whole-genome sequencing and comparative genomics

DNA was isolated from bacterial colonies of the congenic-agr strains without the reporter plasmid grown on Columbia Sheep Blood agar plates (CSB, bioMérieux) and libraries prepared with the QIASeq FX DNA library kit (Qiagen) and sequenced with a MiSeq instrument using MiSeq reagent kit V2 (Illumina). 150 bp paired-end reads were assembled with SPAdes (v3.15.566) with the 'careful' argument and default k-mer size. Annotation was performed with prokka (v1.14.667), and polymorphisms detected with snippy (v4.6.068) using https://github.com/judithbergada/Pipeline_GenomeAnalysis.

RNA-seq analysis

We used congenic-agr strains without the reporter plasmid for the cultures. The Functional Genomics Center Zurich (University of Zurich and ETH Zurich) prepared paired-end 150 bp RNA-seq libraries using the TruSeq RNA Library Prep Kit and sequenced them on an Illumina NovaSeq X Plus without rRNA depletion, yielding on average 35 million reads per sample. We processed data with a custom pipeline: quality control and filtering with fastp (v0.23.469 and FastQC (v0.11.870); rRNA removal using sortmerna (4.3.7, database v4.3.471); alignment to the S. aureus Newman reference genome (RefSeq GCF_000010465.1) with bowtie2 (v2.5.272); and read summarization with featureCounts (subread v2.0.873). We imported counts into R and performed differential expression analysis using DESeq2 (2.0.8v74) and apeglm75 shrinkage, and enhanced genome annotation using AureoWiki pan-genome resources76.

Code availability

Code for image processing is available here: https://doi.org/10.5281/zenodo.18623629. Whole genome sequencing analysis code is available here: https://github.com/judithbergada/Pipeline_GenomeAnalysis (version of commit fb858a0). RNAseq analysis pipeline is available here: https://doi.org/10.5281/zenodo.18492655.

For manuscripts utilizing custom algorithms or software that are central to the research but not yet described in published literature, software must be made available to editors and reviewers. We strongly encourage code deposition in a community repository (e.g. GitHub). See the Nature Portfolio guidelines for submitting code & software for further information.

Data

Policy information about availability of data

All manuscripts must include a data availability statement. This statement should provide the following information, where applicable:

- Accession codes, unique identifiers, or web links for publicly available datasets
- A description of any restrictions on data availability
- For clinical datasets or third party data, please ensure that the statement adheres to our policy

Example movies of one position for each experimental condition are shared as Supplementary Data 1-6 (compressed folders containing multiple labelled mp4 files). Raw image files of all example movies including the output of the fully executed bioimage analysis pipeline are deposited on BioImage Archive with the accession number S-BIAD2927 (https://www.ebi.ac.uk/biostudies/bioimages/studies/S-BIAD2927) for congenic agr-strains and with the accession number S BIAD1046 (https://www.ebi.ac.uk/biostudies/bioimages/studies/S-BIAD1046) for native agr-strains. Additional raw images or processed data are available upon request and are not deposited online due to large file sizes. Retrained StarDist2D segmentation models for the mother-machine and connected-chamber designs as well as the retrained DeLTA 2.0 tracking model and the used training data for these three models has been deposited on Zenodo 10694024 (https://zenodo.org/doi/10.5281/zenodo.10694024). Raw DNA sequencing data of both the congenic and native strains are deposited in the European Nucleotide Archive (ENA) at EMBL-EBI under accession number PRJEB72799 (https://www.ebi.ac.uk/ena/browser/view/PRJEB72799). The 1.5 h samples of the ENA RNA sequencing repository with accession number PRJEB107700 (https://www.ebi.ac.uk/ena/browser/view/PRJEB107700) were used in this study; specific sample identifiers are listed in Supplementary Table 11. Tabular source data for all main and supplementary figures are available in the Figshare repository 31338796 (https://doi.org/10.6084/m9.figshare.31338796).

Data presentation: Fig. 1, 4, and 5 show StarDist segmentation data; Fig. 1D and 4B depict raw single-cell data, while other panels present aggregated metrics. Fig. 2 and 3 display DeLTA tracking data. Connected-chamber data were aggregated into descriptive statistics for 6.5 × 6.5 µm bins or entire chambers. Interaction patterns were classified based on fluorescence intensity during 5-25 h post-inoculation; classification details are provided in Supplementary Methods. One NM-I vs NM-II chamber pair failed to classify unambiguously and was excluded.

Research involving human participants, their data, or biological material

Policy information about studies with human participants or human data. See also policy information about sex, gender (identity/presentation), and sexual orientation and race, ethnicity and racism.

Reporting on sex and gender	*Use the terms sex (biological attribute) and gender (shaped by social and cultural circumstances) carefully in order to avoid confusing both terms. Indicate if findings apply to only one sex or gender; describe whether sex and gender were considered in study design; whether sex and/or gender was determined based on self-reporting or assigned and methods used. Provide in the source data disaggregated sex and gender data, where this information has been collected, and if consent has been obtained for sharing of individual-level data; provide overall numbers in this Reporting Summary. Please state if this information has not been collected. Report sex- and gender-based analyses where performed, justify reasons for lack of sex- and gender-based analysis.*
Reporting on race, ethnicity, or other socially relevant groupings	*Please specify the socially constructed or socially relevant categorization variable(s) used in your manuscript and explain why they were used. Please note that such variables should not be used as proxies for other socially constructed/relevant variables (for example, race or ethnicity should not be used as a proxy for socioeconomic status). Provide clear definitions of the relevant terms used, how they were provided (by the participants/respondents, the researchers, or third parties), and the method(s) used to classify people into the different categories (e.g. self-report, census or administrative data, social media data, etc.) Please provide details about how you controlled for confounding variables in your analyses.*
Population characteristics	*Describe the covariate-relevant population characteristics of the human research participants (e.g. age, genotypic information, past and current diagnosis and treatment categories). If you filled out the behavioural & social sciences study design questions and have nothing to add here, write "See above."*
Recruitment	*Describe how participants were recruited. Outline any potential self-selection bias or other biases that may be present and how these are likely to impact results.*
Ethics oversight	*Identify the organization(s) that approved the study protocol.*

Note that full information on the approval of the study protocol must also be provided in the manuscript.

Field-specific reporting

Please select the one below that is the best fit for your research. If you are not sure, read the appropriate sections before making your selection.

[x] Life sciences [ ] Behavioural & social sciences [ ] Ecological, evolutionary & environmental sciences

For a reference copy of the document with all sections, see nature.com/documents/nr-reporting-summary-flat.pdf

Life sciences study design

All studies must disclose on these points even when the disclosure is negative.

Sample size	No sample size calculation was performed. Experiments were performed at least in biological triplicates. Certain experiments or conditions were repeated more than three times to balance number of observed individual bacterial cells or connected-chambers across conditions. A single mother-machine experiments allows observation of tens of thousands of individual bacterial cells allowing extreme technical replication of results. 3-7 biological replicates are generally deemed acceptable in the field (while some even report on single replicates). Due to similar argumentation, four biological replicates of connected-chamber experiments allowing observations of up to 35 technical replication, each containing around 2000 cells per side, was deemed an acceptable sample size. RNA sequencing in biological triplicates is a widely accepted approach. Given that we observed good agreement between the three replicates and the data is only part of the supplement, we chose to not repeat more. Similarly, qPCR and platereader experiments showed good agreement between the 3 biological replicates and represent a standard number of repeats.
Data exclusions	General: we manually discarded microscopy time-lapse series affected by image registration errors, chamber detection issues, high rates of out-of-focus frames, or obvious cell death, potentially attributable to media switch-induced pressure changes. Programmatically, chambers with low cell counts were excluded. Segmented cells were further filtered according to cell size to include only cells with area (um2) between 0.3 and 2.5. For cell tracking, cells with a tracked lifespan below 10min or above 6h were excluded. For response time calculation, only replicates reaching the threshold of 50% activated bacteria were included. For assessment of intergenerational stability, only replicates with at least 8 lineages in a given founder agr state were included. For connected chamber experiments, only chambers with a median cell number of 700 or higher were included. qPCR samples below the limit of detection (Cq = 30) are indicated in Fig.1g.
Replication	All experiments were repeated at least three times at different days using independent bacterial cultures. Biological replication resulted in good agreement.
Randomization	Each mother-machine chip contained six independent growth channels allowing testing of six conditions. The assignment of condition to channel was randomized. For connected-chamber chips, the order of inoculation of agr-I and the partner type was alternated between biological replicates.
Blinding	No blinding was performed at any step as experiments were performed by the same person which needed to know AIP dilutions and strain agr-type for correct exposure. We do not expect blinding affecting the results.

Reporting for specific materials, systems and methods

We require information from authors about some types of materials, experimental systems and methods used in many studies. Here, indicate whether each material, system or method listed is relevant to your study. If you are not sure if a list item applies to your research, read the appropriate section before selecting a response.

Materials & experimental systems

n/a	Involved in the study
☐	☐ Antibodies
☐	☐ Eukaryotic cell lines
☐	☐ Palaeontology and archaeology
☐	☐ Animals and other organisms
☐	☐ Clinical data
☐	☐ Dual use research of concern
☐	☐ Plants

Methods

n/a	Involved in the study
☐	☐ ChIP-seq
☐	☐ Flow cytometry
☐	☐ MRI-based neuroimaging

Antibodies

Antibodies used

Describe all antibodies used in the study; as applicable, provide supplier name, catalog number, clone name, and lot number.

Validation

Describe the validation of each primary antibody for the species and application, noting any validation statements on the manufacturer's website, relevant citations, antibody profiles in online databases, or data provided in the manuscript.

Eukaryotic cell lines

Policy information about cell lines and Sex and Gender in Research

Cell line source(s)

State the source of each cell line used and the sex of all primary cell lines and cells derived from human participants or vertebrate models.

Authentication

Describe the authentication procedures for each cell line used OR declare that none of the cell lines used were authenticated.

Mycoplasma contamination

Confirm that all cell lines tested negative for mycoplasma contamination OR describe the results of the testing for mycoplasma contamination OR declare that the cell lines were not tested for mycoplasma contamination.

Commonly misidentified lines
(See ICLAC register)

Name any commonly misidentified cell lines used in the study and provide a rationale for their use.

Palaeontology and Archaeology

Specimen provenance

Provide provenance information for specimens and describe permits that were obtained for the work (including the name of the issuing authority, the date of issue, and any identifying information). Permits should encompass collection and, where applicable, export.

Specimen deposition

Indicate where the specimens have been deposited to permit free access by other researchers.

Dating methods

If new dates are provided, describe how they were obtained (e.g. collection, storage, sample pretreatment and measurement), where they were obtained (i.e. lab name), the calibration program and the protocol for quality assurance OR state that no new dates are provided.

☐ Tick this box to confirm that the raw and calibrated dates are available in the paper or in Supplementary Information.

Ethics oversight

Identify the organization(s) that approved or provided guidance on the study protocol, OR state that no ethical approval or guidance was required and explain why not.

Note that full information on the approval of the study protocol must also be provided in the manuscript.

Animals and other research organisms

Policy information about studies involving animals; ARRIVE guidelines recommended for reporting animal research, and Sex and Gender in Research

Laboratory animals

For laboratory animals, report species, strain and age OR state that the study did not involve laboratory animals.

Wild animals

Provide details on animals observed in or captured in the field; report species and age where possible. Describe how animals were

	caught and transported and what happened to captive animals after the study (if killed, explain why and describe method; if released, say where and when) OR state that the study did not involve wild animals.
Reporting on sex	*Indicate if findings apply to only one sex; describe whether sex was considered in study design, methods used for assigning sex. Provide data disaggregated for sex where this information has been collected in the source data as appropriate; provide overall numbers in this Reporting Summary. Please state if this information has not been collected. Report sex-based analyses where performed, justify reasons for lack of sex-based analysis.*
Field-collected samples	*For laboratory work with field-collected samples, describe all relevant parameters such as housing, maintenance, temperature, photoperiod and end-of-experiment protocol OR state that the study did not involve samples collected from the field.*
Ethics oversight	*Identify the organization(s) that approved or provided guidance on the study protocol, OR state that no ethical approval or guidance was required and explain why not.*

Note that full information on the approval of the study protocol must also be provided in the manuscript.

Clinical data

Policy information about clinical studies

All manuscripts should comply with the ICMJE guidelines for publication of clinical research and a completed CONSORT checklist must be included with all submissions.

Clinical trial registration	*Provide the trial registration number from ClinicalTrials.gov or an equivalent agency.*
Study protocol	*Note where the full trial protocol can be accessed OR if not available, explain why.*
Data collection	*Describe the settings and locales of data collection, noting the time periods of recruitment and data collection.*
Outcomes	*Describe how you pre-defined primary and secondary outcome measures and how you assessed these measures.*

Dual use research of concern

Policy information about dual use research of concern

Hazards

Could the accidental, deliberate or reckless misuse of agents or technologies generated in the work, or the application of information presented in the manuscript, pose a threat to:

No	Yes	
x		Public health
x		National security
x		Crops and/or livestock
x		Ecosystems
x		Any other significant area

Experiments of concern

Does the work involve any of these experiments of concern:

No	Yes	
x		Demonstrate how to render a vaccine ineffective
x		Confer resistance to therapeutically useful antibiotics or antiviral agents
x		Enhance the virulence of a pathogen or render a nonpathogen virulent
x		Increase transmissibility of a pathogen
x		Alter the host range of a pathogen
x		Enable evasion of diagnostic/detection modalities
x		Enable the weaponization of a biological agent or toxin
x		Any other potentially harmful combination of experiments and agents

Plants

Seed stocks

Report on the source of all seed stocks or other plant material used. If applicable, state the seed stock centre and catalogue number. If plant specimens were collected from the field, describe the collection location, date and sampling procedures.

Novel plant genotypes

Describe the methods by which all novel plant genotypes were produced. This includes those generated by transgenic approaches, gene editing, chemical/radiation-based mutagenesis and hybridization. For transgenic lines, describe the transformation method, the number of independent lines analyzed and the generation upon which experiments were performed. For gene-edited lines, describe the editor used, the endogenous sequence targeted for editing, the targeting guide RNA sequence (if applicable) and how the editor was applied.

Authentication

Describe any authentication procedures for each seed stock used or novel genotype generated. Describe any experiments used to assess the effect of a mutation and, where applicable, how potential secondary effects (e.g. second site T-DNA insertions, mosiacism, off-target gene editing) were examined.

ChIP-seq

Data deposition

☐ Confirm that both raw and final processed data have been deposited in a public database such as GEO.

☐ Confirm that you have deposited or provided access to graph files (e.g. BED files) for the called peaks.

Data access links
May remain private before publication.

For "Initial submission" or "Revised version" documents, provide reviewer access links. For your "Final submission" document, provide a link to the deposited data.

Files in database submission

Provide a list of all files available in the database submission.

Genome browser session
(e.g. UCSC)

Provide a link to an anonymized genome browser session for "Initial submission" and "Revised version" documents only, to enable peer review. Write "no longer applicable" for "Final submission" documents.

Methodology

Replicates

Describe the experimental replicates, specifying number, type and replicate agreement.

Sequencing depth

Describe the sequencing depth for each experiment, providing the total number of reads, uniquely mapped reads, length of reads and whether they were paired- or single-end.

Antibodies

Describe the antibodies used for the ChIP-seq experiments; as applicable, provide supplier name, catalog number, clone name, and lot number.

Peak calling parameters

Specify the command line program and parameters used for read mapping and peak calling, including the ChIP, control and index files used.

Data quality

Describe the methods used to ensure data quality in full detail, including how many peaks are at FDR 5% and above 5-fold enrichment.

Software

Describe the software used to collect and analyze the ChIP-seq data. For custom code that has been deposited into a community repository, provide accession details.

Flow Cytometry

Plots

Confirm that:

☐ The axis labels state the marker and fluorochrome used (e.g. CD4-FITC).

☐ The axis scales are clearly visible. Include numbers along axes only for bottom left plot of group (a 'group' is an analysis of identical markers).

☐ All plots are contour plots with outliers or pseudocolor plots.

☐ A numerical value for number of cells or percentage (with statistics) is provided.

Methodology

Sample preparation

Describe the sample preparation, detailing the biological source of the cells and any tissue processing steps used.

Instrument

Identify the instrument used for data collection, specifying make and model number.

Software

Describe the software used to collect and analyze the flow cytometry data. For custom code that has been deposited into a community repository, provide accession details.

Cell population abundance	*Describe the abundance of the relevant cell populations within post-sort fractions, providing details on the purity of the samples and how it was determined.*
Gating strategy	*Describe the gating strategy used for all relevant experiments, specifying the preliminary FSC/SSC gates of the starting cell population, indicating where boundaries between "positive" and "negative" staining cell populations are defined.*

☐ Tick this box to confirm that a figure exemplifying the gating strategy is provided in the Supplementary Information.

Magnetic resonance imaging

Experimental design

Design type	*Indicate task or resting state; event-related or block design.*
Design specifications	*Specify the number of blocks, trials or experimental units per session and/or subject, and specify the length of each trial or block (if trials are blocked) and interval between trials.*
Behavioral performance measures	*State number and/or type of variables recorded (e.g. correct button press, response time) and what statistics were used to establish that the subjects were performing the task as expected (e.g. mean, range, and/or standard deviation across subjects).*

Acquisition

Imaging type(s)	*Specify: functional, structural, diffusion, perfusion.*
Field strength	*Specify in Tesla*
Sequence & imaging parameters	*Specify the pulse sequence type (gradient echo, spin echo, etc.), imaging type (EPI, spiral, etc.), field of view, matrix size, slice thickness, orientation and TE/TR/flip angle.*
Area of acquisition	*State whether a whole brain scan was used OR define the area of acquisition, describing how the region was determined.*

Diffusion MRI ☐ Used ☐ Not used

Preprocessing

Preprocessing software	*Provide detail on software version and revision number and on specific parameters (model/functions, brain extraction, segmentation, smoothing kernel size, etc.).*
Normalization	*If data were normalized/standardized, describe the approach(es): specify linear or non-linear and define image types used for transformation OR indicate that data were not normalized and explain rationale for lack of normalization.*
Normalization template	*Describe the template used for normalization/transformation, specifying subject space or group standardized space (e.g. original Talairach, MNI305, ICBM152) OR indicate that the data were not normalized.*
Noise and artifact removal	*Describe your procedure(s) for artifact and structured noise removal, specifying motion parameters, tissue signals and physiological signals (heart rate, respiration).*
Volume censoring	*Define your software and/or method and criteria for volume censoring, and state the extent of such censoring.*

Statistical modeling & inference

Model type and settings	*Specify type (mass univariate, multivariate, RSA, predictive, etc.) and describe essential details of the model at the first and second levels (e.g. fixed, random or mixed effects; drift or auto-correlation).*
Effect(s) tested	*Define precise effect in terms of the task or stimulus conditions instead of psychological concepts and indicate whether ANOVA or factorial designs were used.*

Specify type of analysis: ☐ Whole brain ☐ ROI-based ☐ Both

Statistic type for inference (See Eklund et al. 2016)	*Specify voxel-wise or cluster-wise and report all relevant parameters for cluster-wise methods.*
Correction	*Describe the type of correction and how it is obtained for multiple comparisons (e.g. FWE, FDR, permutation or Monte Carlo).*

Models & analysis

n/a	Involved in the study
☐	☐ Functional and/or effective connectivity
☐	☐ Graph analysis
☐	☐ Multivariate modeling or predictive analysis

Functional and/or effective connectivity	*Report the measures of dependence used and the model details (e.g. Pearson correlation, partial correlation, mutual information).*
Graph analysis	*Report the dependent variable and connectivity measure, specifying weighted graph or binarized graph, subject- or group-level, and the global and/or node summaries used (e.g. clustering coefficient, efficiency, etc.).*
Multivariate modeling and predictive analysis	*Specify independent variables, features extraction and dimension reduction, model, training and evaluation metrics.*
