# Supplementary material for: Single-cell approach dissecting agr quorum sensing dynamics in Staphylococcus aureus
Source: Nat Commun. 2026 May 20;17:8139. doi: 10.1038/s41467-026-73552-9 (PMC13458059; doi:10.1038/s41467-026-73552-9)
Supplement: Supplementary file 2 [file 41467_2026_73552_MOESM12_ESM.pdf]

nature portfolio

Peer Review File

Single-cell approach dissecting *agr* quorum sensing dynamics in *Staphylococcus aureus*

Corresponding Author: Professor Annelies Zinkernagel

This file contains all reviewer reports in order by version, followed by all author rebuttals in order by version.

Version 0:

Reviewer comments:

Reviewer #1

(Remarks to the Author)
Interesting manuscript that dives into the exploring the differences between agr types in S. aureus. There are several interesting findings including that induction of agr leads to extended generation time. The major concerns is that there genetic strain backgrounds interfere with the conclusions that are drawn for the different agr types and that the experimental setup may influence the conclusions drawn about the systems (see below)

Minor issues:
You have too many digits in your numbers (for example line 99 the number 5.51 should be 6 to indicate the level of certainty on the number)

Abstract – does not mention implications concerning interactions with the non-aureus species – this could perhaps increase relevance.

l. 19 what do you mean by intergenerational?

l. 25 the perspective is a bit far-fetched based on the findings listed in the abstract

l. 47 also AgrA regulated virulence gene expression

l. 86-90 mother machine requires a few more words – how do you control that it is single cells going through? (sonication? And how do you check?) And what is the advantaged of this approach?
Figure 1 could be split up to give a more detailed presentation of the mother cell machine

l. 91: the strains used in these experiments are natural carriers of agr I-IV which means that there may be substantial heterogeneity in their genetic backgrounds. This is a problem when analyzing agr activity. The authors measure the differences in expression of key regulators of agr in figure 2 but even though the expression levels of these regulators appear fairly similar (except agrD) then the activity may vary substantial and thus it is hard to draw firm conclusions from the reporter strains.

L. 102 the bimodal distribution: can it be related to the setup so that depending on the location of the cells the agr is more or less expressed? It could look a bit like that from figure 1C,

l. 164 explain the intergenerational with a few more words – does it mean that cells switch agr expression levels upon cell division?
Figure 5B: do the switches (grey lines) indicate the time points shown in figure 5A? they do not seem to match (3,5,4 hours respectively)

l. 189 are all the reporter strains use here producing there own AIPs? In figure 6A there is indicated AIP addition – is that on top of auto-production? Furthermore are the cells growing equally well in the top and bottom chamber? This could influence AIP production and the results obtained. The setup appears somewhat artificial and difficult to make firm conclusions from,

Figure 6 B-E: it would be useful if you in the figures show which agr type is in each of the two chambers.

(Remarks on code availability)

Reviewer #2

(Remarks to the Author)
The authors use sophisticated microfluidics and single-cell technology to elucidate the dynamics of quorum sensing in S. aureus by arg, a global regulator or virulence. While most of our current understanding of the four S. aureus agr types and their cross-activating or cross-inhibitory activities result from studies in batch cultures, the study by Bär et al reveals agr activities under continuous flow conditions, which reflect the in- vivo situation much better than previous approaches. Given the pivotal role of agr in S. aureus infections, the presented data are of high potential relevance. Some parts of the manuscript have a strongly theoretical character, which is at times not easy to comprehend and I am not sure if I understood all parts correctly.

I suggest the authors explain several parts of the manuscript better to allow even reads with limited background in regulatory principles to follow all the messages.

Fig. 1 shows that the agr-III strain does not respond to exogenous AIP and shows agr promoter P3 activity under none of the tested conditions. Given the fact that spontaneous agr mutations can easily occur, what is the authors' evidence that this strain has a functional agr at all? Arg dysfunction would also explain why the agr-III strain shows the shortest generation time. If there is clear evidence for functionality, it should be mentioned in the beginning to clarify this critical point. The discussion of agr-III at several parts of the manuscript should be reconsidered in the light of knowledge on agr functionality or dysfunction.

Fig. 1E shows P3 activity for three of the test strains under two different conditions – at rather early time points upon addition of exogenous AIP, and at late time points without AIP addition. I suppose, the second is due to autoinduction upon accumulation of endogenous AIP? This point should be explained better when results from 1E are described.

The authors describe P3 activity as "bimodal" but it remains elusive what this means. What exactly shows two modes here? Please explain this point better, also in the abstract.

The authors analyze several regulators of agr promoters including sarX, sarT, and mgrA. It would be helpful to find a schematic figure in the manuscript describing, which regulator controls which of the arg promoters.

Page 7, second paragraph: the four different agr interaction classes are interesting to note, yet not easy to explain and therefore somewhat puzzling. What is known about affinities of the various AIPs for the AIP receptor AgrC? Can such affinities be calculated based on AlphFold data? Could affinity differences explain the different behaviors to some extent?

Last part of the results: I found this section particularly difficult to comprehend. Please consider rephrasing to help readers to understand the messages.

Specific points:
Lines 77 and 89: please explain "mother machine".
Line 129: what exactly is meant with "agr regulators"? Do the authors refer to AgrA? Please rephrase to clarify.
Lines 165-168: Difficult to comprehend, please rephrase.
Line 282: Do the authors refer here to AIP structure? Please clarify.

(Remarks on code availability)

Reviewer #3

(Remarks to the Author)
Nature Communications manuscript NCOMMS-24-15425-T

Bär et al.

Title: "Single-cell approach dissecting agr quorum sensing dynamics in Staphylococcus aureus."

Staphylococcus aureus is a versatile pathogen that encodes a wide array of virulence factors to respond to different challenges in the host. The production of these factors is carefully regulated in response to environmental cues to ensure they are produced when needed. Therefore, the regulation of accessory factors plays a key role in the pathogen's ability to invade and infect the host.

S. aureus encodes 16 two-component systems (TCSs), but the primary regulator of virulence is the quorum sensing (QS)

accessory gene regulator (agr) TCS. The agr operon is comprised of two divergent transcripts, RNAII and RNAIII, that are transcribed from the opposed promoters P2 and P3, respectively. RNAII encodes the TCS and QS proteins of the agr system and RNAIII is the intercellular effector. This complex system has been studied for decades by numerous groups, but mostly in the context of bacterial communities and not at the single-cell level.

In this manuscript by Bär et al., the authors employed microfluidics, time-lapse microscopy, and other methods to study agr regulation at the single-cell level to identify differences in AIP sensitivity among agr-types that could not be discerned previously via the analyses of bulk cultures. This kind of study is important, and the results are interesting and reflect technical expertise. However, more attention should have been paid to the S. aureus strains, as the choice of plasmids and constructs severely limits the relevance and applicability of the results.

Specific remarks:

1. P3 L91 and P11 L318: The Greenberg lab made pDB59 and PMID: 14996815 is the correct reference.

2. P3 L91 and P11 L318: Plasmid pDB59 contains the agr P2-P3 region amplified from strain MN8 to generate agr P3-yfp. This turns out to be a very important detail because the MN8 P2-P3 region has several mismatches when compared to the P2-P3 regions of the four strains used in this study. While a few mismatches here and there are not necessarily cause for alarm, one of the mismatches is particularly important: MN8 has a mismatch in the -35 element of P3. From what I can tell, the P3s of at least three of the strains used in this study (I could not obtain the genome of MNTG) do not have this MN8 -35 mismatch.

LAC P3 -35: TTGGAA
502a P3 -35: TTGGAA
MW2 P3 -35: TTGGAA
MN8 P3 -35: ATGGAA

The MN8 mismatch is a mutation away from -35 consensus, so RNA polymerase likely interacts differently with the MN8 P3 than the P3s of the four strains. Also, depending on the AgrA study you look at, this position is either within an AgrA binding site (PMID: 15516566) or immediately adjacent to an AgrA binding site (PMID: 28955870 and 21908676), so there is a possibility that this mutation affects AgrA binding. Therefore, pDB59 was not a good choice for this study because the MN8 P2-P3 region does not reflect the agr systems of the selected strains.

3. P3 L91 and P11 L318: Plasmid pDB59 is a multicopy plasmid. This is highly problematic to this study for three reasons. The first is that AgrA is known to have a higher affinity for the P2 promoter. Plasmid pDB59 is derived from pDB39, which is a shuttle vector that uses a pC194 replicon for S. aureus replication. Normally, pC194 will average around 100 copies per cell. This means that phosphorylated AgrA will need to reach levels high enough to saturate the many copies of P2 before it starts to bind to and activate a P3. This is very different than how it works with the native P2-P3.

The second is that multicopy plasmids vary in copy number from cell to cell. Unfortunately, this is where a single-cell study works against the authors because it is not possible to control the copy number precisely for any given cell. Numerous factors, such as the precise concentration of antibiotics in the media, will influence the precise plasmid copy number. Naturally, P3 activation in any given cell will be affected by plasmid copy number.

The third is that once P3 is activated, it is one of the strongest promoters in S. aureus. For this reason, the agr locus cannot be stably maintained on an episomal plasmid, even on ones that are extremely low copy, because mutations are strongly selected owing to the extremely high metabolic burden. Likewise, it is well known among groups that work on agr that P3 fusions on multicopy plasmids are highly unstable and rapidly accumulate mutations in P3. This has even been documented a few times (PMID: 25192956). Admittedly, the MN8 P3 may not have this problem because of the -35 element mutation, though it is likely that it also has this issue.

To avoid the aforementioned issues, P3 fusions should be maintained in single-copy integration vectors. The P3 promoter, in a single copy, is strong enough if that is a concern.

4. P11 L317: Four agr-types were used for this study: LAC, agr-I, ST8; 502a, agr-II; MW2, agr-III; and MNTG, agr-IV. The issue that I see here is that different agr types are compared but the strain backgrounds are not isogenic. It is more accurate to say AIP sensitivity among four strains was compared, rather than four agr-types.

There are established experimental systems that control for the strain background when comparing different agr-types (PMID: 22467783). The four agr-types can be individually inserted into a single strain background with the native agr locus deleted. This should have been done here. Otherwise, there are too many variables that come with different strain backgrounds.

Less substantial remarks:

1. Figure 1,3: Please give a brief description of controls.

2. Controls such as agr deletions would be good to add.

3. P15 L443: “To assess baseline gene expression profiles of all four strains, overnight cultures grown at 37° C in TSB with Cm10 and 0.01% Tween20 were diluted 1:20 in fresh medium and regrown for 1.5 h.”

Typically, a single subculture of an overnight does not result in true baseline agr activity. The overnight culture is full of AIP and a 1:20 dilution still has sufficient AIP to activate QS. Generally, subculturing a second or third time with exponentially growing cultures will achieve a baseline.

(Remarks on code availability)
NA

Version 1:

Reviewer comments:

Reviewer #1

(Remarks to the Author)
I am happy with the changes and acknowledge the large amount of extra work that has been performed to accommodate mine and other reviewers concerns.

(Remarks on code availability)

Reviewer #2

(Remarks to the Author)
All my concerns have been appropriately addressed by the authors.

(Remarks on code availability)

Reviewer #3

(Remarks to the Author)
Nature Communications manuscript NCOMMS-24-15425A-Z

Bär et al.

Title: “Single-cell approach dissecting agr quorum sensing dynamics in Staphylococcus aureus.”

The authors have made a substantial effort to address the reviewers’ comments, and the revised manuscript represents a clear improvement over the original submission. In particular, the new analysis of baseline gene expression across agr types (Supplementary Figure 5) is well executed and adds a layer of clarity to the study.

My previous concerns have been satisfactorily addressed, and I have no further comments.

(Remarks on code availability)
NIL

Open Access This Peer Review File is licensed under a Creative Commons Attribution 4.0 International License, which permits use, sharing, adaptation, distribution and reproduction in any medium or format, as long as you give appropriate credit to the original author(s) and the source, provide a link to the Creative Commons license, and indicate if changes were made.
In cases where reviewers are anonymous, credit should be given to 'Anonymous Referee' and the source.
The images or other third party material in this Peer Review File are included in the article's Creative Commons license, unless indicated otherwise in a credit line to the material. If material is not included in the article's Creative Commons license and your intended use is not permitted by statutory regulation or exceeds the permitted use, you will need to obtain permission directly from the copyright holder.
To view a copy of this license, visit https://creativecommons.org/licenses/by/4.0/

Reviewer #1 (Remarks to the Author):

Interesting manuscript that dives into the exploring the differences between agr types in S. aureus. There are several interesting findings including that induction of agr leads to extended generation time. The major concerns is that there genetic strain backgrounds interfere with the conclusions that are drawn for the different agr types and that the experimental setup may influence the conclusions drawn about the systems (see below)

Minor issues:

You have too many digits in your numbers (for example line 99 the number 5.51 should be 6 to indicate the level of certainty on the number)

Thank you for pointing this out. We have carefully reviewed all numerical values in the manuscript and adjusted the number of displayed digits to reflect the level of certainty in the measurements.

Abstract – does not mention implications concerning interactions with the non-aureus species – this could perhaps increase relevance.

We appreciate this suggestion and agree that the implications regarding interactions with non-aureus species are important. However, due to strict word count limitations in the abstract and the fact that we did not perform experiments addressing these interactions, we decided not to include this aspect at this stage. We acknowledge its relevance and mention this important aspect in the discussion.

l. 19 what do you mean by intergenerational?

The rewritten abstract no longer mentions intergenerational stability. Instead, we provide a more detailed explanation in the corresponding results section (L207+). In brief, our goal was to quantify whether the *agr*-state of bacterial cells persists through multiple division events. This question is particularly relevant for cells that did not activate their *agr*-system despite AIP stimulation: Is this state only temporary, or do such cells produce offspring that remain in a deactivated state?

l. 25 the perspective is a bit far-fetched based on the findings listed in the abstract

We appreciate this observation. The abstract now is focused on results. The last sentence now reads: "Our approach links single-cell signaling to population outcomes and uncovers *agr*-type-specific asymmetries with potential consequences for strain competition and virulence."

l. 47 also AgrA regulated virulence gene expression

Thank you for pointing this out. We have expanded the description of virulence regulation by the *agr*-system to include direct AgrA regulation. The revised sentence now reads: "At high extracellular AIP concentrations, phosphorylated AgrA additionally activates P3 and directly upregulates phenol-soluble modulins (PSMs) production[9]."

l. 86-90 mother machine requires a few more words – how do you control that it is single cells going through? (sonication? And how do you check?) And what is the advantaged of this approach?

Thank you for this helpful comment. We have clarified the mother-machine methodology in the revised manuscript. In our study, "single-cell resolution" refers to the ability to observe and quantify *agr*-activation and cell-physiological traits at the level of individual cells, rather than ensuring that each chamber begins with exactly one founder cell.

Seeding of mother-machine chambers.

Cells are introduced by pushing a concentrated, dense exponential-phase culture through the main channel. Individual bacteria stochastically enter the side chambers, typically resulting in one to a few cells per chamber. No sonication or special single-cell loading procedure is used. This loading is stressful and we let bacteria recover overnight which additionally ensures full *agr*-inactivation. This loading procedure is described in the Methods section starting at L554.

How we verify single-cell resolution.

Each chamber has a height of 0.93 µm, which physically restricts cells to a single optical plane, enabling reliable single-cell segmentation, fluorescence quantification, and lineage tracking over time. Chambers containing multilayered growth do not occur because the height constraint prevents vertical stacking. Cell number per chamber remains constant as division events push out excess bacteria.

Advantages of the mother-machine approach.

The confinement of cells to a single focal plane, combined with high-frequency imaging, allows us to track divisions and fluorescence changes of individual cells throughout the experiment. Mother-machine microfluidic platforms provide precise and stable chemical control: medium from the large flow channel continuously diffuses into the shallow chambers, preventing accumulation of secreted factors such as AIPs and metabolites and ensuring that cells experience the exact external environment intended for stimulation. This setup is therefore ideally suited to dissect *agr*-activation dynamics, heterogeneity, and timing at single-cell resolution under fully defined and constant conditions, features that cannot be achieved in bulk culture.

We have added a concise description of these points to the Results (starting at L96) and expanded methodological details in the Methods section, including device dimensions and imaging workflow.

Figure 1 could be split up to give a more detailed presentation of the mother cell machine

Thank you for the suggestion. Since the mother-machine design was established previously and not developed by us, we decided not to include the illustration of it as a separate figure, as this has already been comprehensively presented in the original publications introducing the technique. However, we have added more descriptive details in the first section of the results and corresponding methods section to improve clarity and anticipate that this will provide sufficient understanding of the experimental tool.

l. 91: the strains used in these experiments are natural carriers of agr I-IV which means that there may be substantial heterogeneity in their genetic backgrounds. This is a problem when analyzing agr activity. The authors measure the differences in expression of key regulators of

agr in figure 2 but even though the expression levels of these regulators appear fairly similar (except agrD) then the activity may vary substantial and thus it is hard to draw firm conclusions from the reporter strains.

We thank the reviewer for highlighting this important issue. We fully agree that comparing *agr*-activity across strains with different genetic backgrounds can confound interpretation, as background-specific regulatory differences may mask or mimic *agr*-type–dependent behavior. This concern was also raised by another reviewer.
To eliminate genetic background effects, we repeated all experiments using the congenic-*agr* strain set generated by Novick's group (PMID: 22467783), in which the native-*agr*-locus of Newman is deleted and replaced with *agr*-I, -II, -III, or -IV. We constructed perfectly P3-matched GFP reporter plasmids for each congenic strain and repeated the microfluidic, and RNA-seq experiments and performed complementary qPCR validation under these controlled genetic conditions.
Using these congenic strains, we confirmed all major observations from the initial submission, including differences in AIP sensitivity, delayed activation of *agr*-III, elevated basal activity of *agr*-IV, and the presence of *agr*-bimodality. Some findings seen only in native strains, such as the markedly increased generation time of 502a-II, did not occur in the *agr*-II congenic strain and are therefore attributable to genetic background rather than *agr*-type.
Importantly, RNA-seq of the congenic strains from fully *agr*-reset early exponential revealed highly similar baseline expression profiles across all *agr*-types, including the *agr*-locus and known *agr*-regulators (new Supplementary Fig. 5). This demonstrates that differences seen in microfluidics arise from *agr*-type-specific activation dynamics rather than pre-existing transcriptional differences.
The RNA-seq dataset from the initial submission has been removed entirely. Another reviewer rightly pointed out that the original culturing protocol did not fully reset *agr* due to insufficient subculturing, making those data unreliable for baseline comparisons. Only the new, technically correct dataset generated with congenic strains is included in the revised manuscript.
Together, the new congenic dataset and the native-strain observations from the initial submission provide a coherent and robust picture of how *agr*-type influences AIP sensitivity and interaction outcomes.

L. 102 the bimodal distribution: can it be related to the setup so that depending on the location of the cells the agr is more or less expressed? It could look a bit like that from figure 1C,

Thank you for suggesting this additional quality control analysis. To address this, we performed a statistical analysis of the effect of y-position within the chamber on $\log_{10}$ fluorescence intensity using a mixed-effects model. The model included random intercepts for replicate and chamber, and fixed effects for the three-way interaction of *agr*-type, AIP concentration, and y-position. We have included a visualization of this analysis in Supplementary Fig. 15.
While some slopes were statistically significant due to the large sample size, the average $\log_{10}$ slope of y-position was only -0.0049 AU/µm. This translates to a difference of approximately 1.3 AU across a 25 µm chamber depth, far below the 100 AU threshold used to classify bacteria as *agr*-activated or deactivated. This is further supported by theoretical diffusion of nutrients and AIP into the chambers detailed now together with the new Supplementary Fig.15 in the methods (L527+). In summary, we do not expect the position to be responsible for bimodal *agr*-activation distributions.

l. 164 explain the intergenerational with a few more words – does it mean that cells switch agr expression levels upon cell division?

Thank you for pointing this out. By "intergenerational stability of *agr*-activation state" we refer to the following: when examining the lineage (up to 10 generations) of descendants from a bacterial cell that divided early during AIP exposure, we assessed how many cells in that lineage retained the same *agr*-activation state as the original cell. If most descendants maintained the same state, we defined this as intergenerational stability of the *agr*-state within that lineage. We have added more details describing this definition in the Results section (L207+) and expanded the explanation in the Methods section (L624+).

Figure 5B: do the switches (grey lines) indicate the time points shown in figure 5A? they do not seem to match (3,5,4 hours respectively)

Thank you for catching this inconsistency. This figure is now presented as Fig. 4 and associated Supplementary Figs. 9-10. The gray lines in Fig. 4b indeed indicate the time points of AIP condition switches. We have corrected the annotations and the time points mentioned in Fig. 4a. Specifically, switch 1 (from homologous to mixed homologous + heterologous AIP) occurred at 3.5 h, and switch 2 (back to homologous alone) occurred at 7.5 h.

l. 189 are all the reporter strains use here producing there own AIPs? In figure 6A there is indicated AIP addition – is that on top of auto-production?

Thank you for pointing out this ambiguity. This figure is now Fig. 5. We apologize for the unclear schematic. No AIP was added to the medium; the label "medium flow -AIP" was intended to indicate "minus AIP," meaning no exogenous AIP was added. We have revised this to "medium flow without AIP" for clarity in the figure. Only self-produced AIP can activate or inhibit the *agr*-system in these connected chambers as no synthetic AIP were supplied in the growth medium. All strains used in these types of experiments were able to produce their own AIP to a high enough level for *agr*-autoinduction, as seen in control experiments in which the same *agr*-type was growing on both sides of the connected-chambers (Supplementary Fig. 10). We have added further clarification in the Results section (L283+) and updated the figure legend accordingly.

Furthermore are the cells growing equally well in the top and bottom chamber? This could influence AIP production and the results obtained.

Thank you for raising this concern. The bacterial cells grow equally well in both chambers. Physically, the chambers are mounted flat, so there is no difference in z-height. We have added details in the Results section (L287+): "Each chamber was supplied by its own 20 µm-high flow channel perfused at 0.25 ml h^{-1} by growth medium without synthetic AIP, creating advection in the flow channels and diffusion-limited exchange into the shallow chambers. This geometry prevents nutrient competition and cell translocation while permitting accumulation and diffusion of self-produced AIPs, enabling *agr*-crosstalk." Additional details are provided in the Methods section (L537+). The nano-channels connecting the two chambers are the only means of interaction between populations, while both chambers receive identical nutrient supply on the other side of the chamber.

The setup appears somewhat artificial and difficult to make firm conclusions from, Figure 6 B-E: it would be useful if you in the figures show which agr type is in each of the two chambers.

Thank you for this comment. The corresponding data are now presented in Fig. 5. We acknowledge that the connected-chambers system, like many experimental setups, represents an artificial simplification of reality. However, it provides a unique opportunity to study interactions between bacterial populations mediated by secreted substances without requiring fluorescent tagging for strain identification. The reported use case models potential interactions between two *agr*-types during for example colonization of a body site. We could hypothesize that simultaneous activation of two *agr*-types could lead to stronger-than-intended *agr*-activation, potentially causing tissue damage and triggering immune responses. Furthermore, this system can be readily adapted to study interactions with other staphylococcal species producing distinct AIPs or with other commensal and pathogenic species.
We also note that repeating these experiments with congenic-*agr* strains resulted in more consistent interaction patterns, which appear to depend primarily on *agr*-type rather than uncontrolled factors such as initial seeding density. This highlights the important contribution of genetic background to any interaction study.
Initially, we refrained from labeling *agr*-types in the figure panels because the images were intended as representative examples of interaction classes, which were observed across multiple *agr*-type combinations. However, we understand that including this information improves clarity and relevance. We have therefore added labels indicating the *agr*-types in each chamber as "example *agr*" in the figure panels.

Reviewer #2 (Remarks to the Author):

The authors use sophisticated microfluidics and single-cell technology to elucidate the dynamics of quorum sensing in S. aureus by agr, a global regulator or virulence. While most of our current understanding of the four S. aureus agr types and their cross-activating or cross-inhibitory activities result from studies in batch cultures, the study by Bär et al reveals agr activities under continuous flow conditions, which reflect the in- vivo situation much better than previous approaches. Given the pivotal role of agr in S. aureus infections, the presented data are of high potential relevance. Some parts of the manuscript have a strongly theoretical character, which is at times not easy to comprehend and I am not sure if I understood all parts correctly.

I suggest the authors explain several parts of the manuscript better to allow even reads with limited background in regulatory principles to follow all the messages.

We thank the reviewer for this helpful suggestion. Because Nature Communications reaches a broad readership, we have revised the manuscript to make the regulatory context and chosen analysis approaches more accessible. Specifically, we expanded the introduction to provide a clearer overview of the *agr*-regulatory architecture, simplified several technically dense sections in the Results, and added concise explanations to guide readers through the rationale of each analysis. The Discussion was also restructured to emphasize the regulatory implications of our findings in a manner that is understandable to non-specialists of quorum sensing systems. We believe these changes substantially improve clarity and readability while preserving scientific precision.

Fig. 1 shows that the agr-III strain does not respond to exogenous AIP and shows agr promoter P3 activity under none of the tested conditions. Given the fact that spontaneous agr mutations can easily occur, what is the authors' evidence that this strain has a functional agr at all? Arg dysfunction would also explain why the agr-III strain shows the shortest generation time. If there is clear evidence for functionality, it should be mentioned in the beginning to clarify this critical point. The discussion of agr-III at several parts of the manuscript should be reconsidered in the light of knowledge on agr functionality or dysfunction.

We thank the reviewer for raising this important point. The native *agr*-III strain used in the initial submission (MW2) indeed showed almost complete absence of *agr*-activation, which could raise concerns regarding *agr* functionality. However, several independent lines of evidence confirm that *agr*-III of the chosen strain is functional.

1. The native *agr*-III strain (MW2) is known to possess an intact *agr*-system.
MW2 is a widely used laboratory strain for studying community-associated-MRSA (USA400 lineage), and prior work has demonstrated *agr*-activity, including studies of spontaneous loss and reversion of *agr*-function (PMID: 31387900). Whole-genome sequencing of our MW2 stock likewise revealed no disruptions in the *agr*-locus (genome available at: https://www.ebi.ac.uk/ena/browser/view/PRJEB72799).
2. Batch-culture plate-reader assays confirmed *agr*-III autoinduction.
When cultured under conditions that allow self-produced AIP to accumulate, MW2 produced a clear P3-YFP signal, albeit with a delay relative to other *agr*-types (Supplementary Fig. 1d-f). This delay is consistent with reduced sensitivity rather than dysfunction.

3. All key experiments were repeated using a congenic *agr*-III strain in an identical genetic background.
To eliminate any background-related confounding, we repeated the core experiments with congenic-*agr* strains (Newman genetic background, native-*agr* replaced with any *agr*-type). The congenic *agr*-III strain also displayed:
• measurable but delayed P3 activation in plate-reader assays (Supplementary Fig. 1a-c), and
• clear but delayed autoinduction in connected-chamber assays when paired with itself (Supplementary Fig. 10).
These results confirm that delayed *agr*-activation is an intrinsic feature of *agr*-III, not a mutation in MW2.
4. The shorter generation time is background-dependent, not *agr*-III-dependent.
The congenic *agr*-III strain in the Newman background showed no reduction in generation time compared with other congenic *agr*-types in the absence of AIP (Supplementary Fig. 7a). This indicates that the shorter generation time observed in MW2 (Supplementary Fig.7b) is attributable to its genetic background, not to *agr*-III per-se.
5. We now clarify *agr*-III functionality early in the Results.
To prevent any ambiguity, we have added a statement at the beginning of the Results (L111): "We validated reporter performance and AIP activity in bulk cultures using a plate-reader, confirming a functional *agr*-system in all strains as well as the modulation of *agr*-activation strength and timing upon the addition of synthetic, homologous AIP (Supplementary Fig.1a-f)."
In summary: all our functional assays, across both native and congenic strains, support that *agr*-III is functional but markedly less sensitive, leading to delayed activation. We have revised the manuscript to make this explicit and to ensure that interpretation of *agr*-III reflects these data.

Fig. 1E shows P3 activity for three of the test strains under two different conditions – at rather early time points upon addition of exogenous AIP, and at late time points without AIP addition. I suppose, the second is due to autoinduction upon accumulation of endogenous AIP? This point should be explained better when results from 1E are described.

We thank the reviewer for highlighting this point. To avoid misunderstanding, we now clarify that endogenously produced AIP cannot accumulate in the mother-machine, as the continuous nutrient-rich flow rapidly removes secreted peptides. Consequently, autoinduction does not occur in this setup and the *agr*-system can only be activated with synthetic, exogenously supplied AIP. We have explicitly added this explanation to the first Results section (L97+).
Figure 1f does not show *agr*-activity under different culture conditions; rather, it presents histograms derived from the same time-series data shown in Fig. 1d, aggregated over three defined intervals that summarize the activation trajectory:

"pre": the 2 h immediately before AIP addition (baseline P3 activity),
"+AIP": the final 2 h of synthetic AIP exposure (fully induced state),
"post": 3–5 h after AIP removal (recovery to *agr*-OFF dynamics).

This representation allows clearer visualization of bimodal activation distributions, highlighting, for example, that *agr*-IV exhibits both a higher baseline ("pre") and a more prolonged activated state ("post") relative to other *agr*-types. The activity seen at the "post" timepoint can be explained by slow downregulation of the *agr*-system. As the GFP signal is

lost rapidly in some *agr*-types, we can assume that the remaining GFP signal is not due to slow GFP degradation.
To address the reviewer's concern, we updated the figure legend and refined the Results text accordingly (L134+).
We believe these changes clarify that the late activation observed in Fig. 1f reflects persistence of synthetic AIP-induced activation, not endogenous AIP accumulation.

The authors describe P3 activity as "bimodal" but it remains elusive what this means. What exactly shows two modes here? Please explain this point better, also in the abstract.

We thank the reviewer for pointing out the need for clearer wording. We now explicitly define what we mean by "bimodal" both in the abstract and in the Results section. In the revised manuscript (L134+), we added: "Distributions of single-cell *agr*-activation in these mother-machine experiments were bimodal at specific observation intervals corresponding to the three experimental phases (Fig.1f). Here, "bimodal" refers to two distinct peaks in the histograms, representing *agr*-OFF and *agr*-ON cells, which revealed pronounced QS heterogeneity at low to intermediate AIP concentrations (4 – 64 nM)." This phrasing clarifies that the bimodality refers to the distribution of single-cell P3-GFP intensities, not to separate experimental conditions. We also updated the abstract to briefly convey this definition. We believe these changes address the reviewer's concern and make the concept of bimodality fully clear to a broad readership.

The authors analyze several regulators of agr promoters including sarX, sarT, and mgrA. It would be helpful to find a schematic figure in the manuscript describing, which regulator controls which of the arg promoters.

We thank the reviewer for this suggestion. In the revised manuscript, the RNA-seq analysis has been substantially reduced and moved to the Supplementary Information. The initial submission included RNA-seq of the native-*agr* strains which were completely removed following another reviewer's concerns regarding experimental design. We now present only the repeated, cleaner dataset generated after triple 1.5 h subculturing of the congenic-*agr* strains to fully reset *agr*-activation and eliminate residual AIP. Importantly, the main text no longer analyzes *sarX, sarT, mgrA*, or other *agr*-associated regulators specifically, and therefore a schematic detailing their regulatory interactions would no longer be relevant to the streamlined scope of the paper. Instead, our Results section regarding RNA-seq is only stating the absence of any transcriptional differences between the four congenic-*agr* strains. Given these adjustments, we believe adding a schematic figure would no longer enhance clarity and could distract from the focused contribution of this study.

Page 7, second paragraph: the four different agr interaction classes are interesting to note, yet not easy to explain and therefore somewhat puzzling. What is known about affinities of the various AIPs for the AIP receptor AgrC? Can such affinities be calculated based on AlphFold data? Could affinity differences explain the different behaviors to some extent?

We thank the reviewer for these insightful questions. Affinity differences between AIPs and their cognate AgrC receptors indeed provide important mechanistic context for interpreting the interaction regimes we observe. The most comprehensive assay can be found in Lyon et al.

(2002), where they quantified activation and inhibition across all four *agr*-types using a standardized *Δagr S. aureus* RN6911 background complemented with plasmids expressing single AgrC alleles and a P3–blaZ reporter (Biochemistry 2002, https://doi.org/10.1021/bi026049u). This system isolated receptor-ligand interactions independently of other *agr*-components.

Their data show that homologous EC_{50} values for AgrC-I–IV (AIP-I–IV) all fall in the low-nanomolar range (≈13–30 nM), whereas inhibitory IC_{50} values differ far more dramatically between receptor–ligand pairs. Notably, AgrC-III exhibits very strong inhibition by heterologous AIPs (e.g., IC_{50} ≈ 1–3 nM for AIP-I, -II, -IV), whereas AgrC-I is inhibited only at much higher concentrations (40, and 70 nM for AIP-II and -III). These biochemical properties are consistent with our findings: the strong inhibition of *agr*-III by AIP-I aligns with the prevalent dominance of *agr*-I over *agr*-III in our congenic co-cultures, while the weaker inhibition and partial cross-activation between AIP-IV and AgrC-I match the stable dual-activation observed for the I/IV pairing.

Regarding the use of AlphaFold: according to our understanding, current structural-prediction methods, including AlphaFold-Multimer, cannot reliably estimate peptide-receptor binding affinities or rank interaction strengths, particularly for membrane-embedded histidine kinases such as AgrC. These approaches predict static structures but not the conformational energetics that determine activation versus inhibition. We therefore consider computational affinity predictions outside the scope of this study.

Overall, our connected-chamber interaction classes can be partly explained by known AIP–AgrC affinity and inhibition patterns, while the increased diversity observed in native strain pairings likely reflects additional genetic-background-dependent factors such as AIP production & maturation rates, protease activity, and membrane composition. We have added clarifying statements in the Discussion to help readers understand how published affinity data relate to the interaction regimes observed in our microfluidic co-cultures.

Last part of the results: I found this section particularly difficult to comprehend. Please consider rephrasing to help readers to understand the messages.

We thank the reviewer for pointing out that the final section of the Results was difficult to follow. We agree that the original statistical modeling description was dense and that the presentation could be clearer. Because the repeated experiments performed with the new congenic strain collection produced much simpler and more interpretable interaction classes, we have substantially streamlined this part of the manuscript.

Specifically, the detailed statistical analysis is now moved primarily to the Supplement, and the main text focuses only on the key message. The revised Results now state (L323): "We quantified early and late community descriptors to assess whether simple population features could explain the observed interaction classes beyond involved *agr*-types. Early community characteristics, such as initial cell numbers, early *agr*-activation, as well as chamber-wide cell density and cell-number fluctuations carried little explanatory power in congenic strains but partly explained the interaction outcomes in native strains (Supplementary Fig.14)."

We also expanded the legend of Supplementary Fig. 14 to provide a clear explanation of the modeling approach, the variables used, and how to interpret the multinomial regression results. This restructuring separates the technical details from the main narrative and improves readability for a broad audience. We hope these revisions make the conclusions of this analysis more accessible and easier to understand.

Specific points:
Lines 77 and 89: please explain “mother machine”.

We thank the reviewer for raising this point. We now provide a concise explanation of the mother-machine microfluidic device in the first Results section (L97+), where it is introduced and used experimentally. Because this paragraph in the Introduction is meant only to summarize the study design, we opted not to duplicate the full description here.

Line 129: what exactly is meant with “agr regulators”? Do the authors refer to AgrA? Please rephrase to clarify.

We thank the reviewer for this comment. In the revised manuscript, the analysis that previously mentioned “*agr* regulators” has been restructured. This section was based on the earlier experimental design, which another reviewer correctly identified as flawed. After repeating the experiments with the congenic strain set, we found no significant transcriptional differences among the strains during early exponential phase. For this reason, neither AgrA nor any other *agr*-associated regulators are discussed at this point in the manuscript anymore. The potential ambiguity has therefore been resolved in the revised version.

Lines 165-168: Difficult to comprehend, please rephrase.

We thank the reviewer for this suggestion. In the revised manuscript, we clarified this sentence to ensure that the meaning is explicit. The updated text now reads (L208): “We quantified *agr*-activation of bacteria over their lifespan (Fig.2a) to investigate if the observed bimodality of *agr*-activation, characterized by the presence of *agr*-OFF and *agr*-ON cells (Fig.1f), could arise from frequent switching between agr-states at the single-cell level.” This revised phrasing both re-explains the concept of *agr*-bimodality and states our hypothesis more clearly, which we hope improved understanding.

Line 282: Do the authors refer here to AIP structure? Please clarify.

We thank the reviewer for pointing out this ambiguity. In the revised manuscript, we now explicitly refer to AIP sequence variation, which is the relevant determinant in this context. The updated sentence in the Discussion (L422+) reads: “. Second, even subtle AIP sequence variations allow AgrC binding but prevent full activation, producing competitive inhibition or QS quenching[25,46].” This revised phrasing avoids the term “structure,” makes it clear that we are referring specifically to sequence-level differences in the AIPs, and should improve reader understanding.

Reviewer #3 (Remarks to the Author):

Nature Communications manuscript NCOMMS-24-15425-T
Bär et al.

Title: "Single-cell approach dissecting agr quorum sensing dynamics in Staphylococcus aureus."

Staphylococcus aureus is a versatile pathogen that encodes a wide array of virulence factors to respond to different challenges in the host. The production of these factors is carefully regulated in response to environmental cues to ensure they are produced when needed. Therefore, the regulation of accessory factors plays a key role in the pathogen's ability to invade and infect the host.

S. aureus encodes 16 two-component systems (TCSs), but the primary regulator of virulence is the quorum sensing (QS) accessory gene regulator (agr) TCS. The agr operon is comprised of two divergent transcripts, RNAII and RNAIII, that are transcribed from the opposed promoters P2 and P3, respectively. RNAII encodes the TCS and QS proteins of the agr system and RNAIII is the intercellular effector. This complex system has been studied for decades by numerous groups, but mostly in the context of bacterial communities and not at the single-cell level.

In this manuscript by Bär et al., the authors employed microfluidics, time-lapse microscopy, and other methods to study agr regulation at the single-cell level to identify differences in AIP sensitivity among agr-types that could not be discerned previously via the analyses of bulk cultures. This kind of study is important, and the results are interesting and reflect technical expertise. However, more attention should have been paid to the S. aureus strains, as the choice of plasmids and constructs severely limits the relevance and applicability of the results.

Specific remarks:

1. P3 L91 and P11 L318: The Greenberg lab made pDB59 and PMID: 14996815 is the correct reference.

Thank you for pointing out that mistake, the correct reference is now cited both at first mentioning in the Results as well as in the Methods section.

2. P3 L91 and P11 L318: Plasmid pDB59 contains the agr P2-P3 region amplified from strain MN8 to generate agr P3-yfp. This turns out to be a very important detail because the MN8 P2-P3 region has several mismatches when compared to the P2-P3 regions of the four strains used in this study. While a few mismatches here and there are not necessarily cause for alarm, one of the mismatches is particularly important: MN8 has a mismatch in the -35 element of P3. From what I can tell, the P3s of at least three of the strains used in this study (I could not obtain the genome of MNTG) do not have this MN8 -35 mismatch.

LAC P3 -35: TTGGAA
502a P3 -35: TTGGAA

MW2	P3	-35:	TTGGAA
MN8	P3	-35:	ATGGAA

The MN8 mismatch is a mutation away from -35 consensus, so RNA polymerase likely interacts differently with the MN8 P3 than the P3s of the four strains. Also, depending on the AgrA study you look at, this position is either within an AgrA binding site (PMID: 15516566) or immediately adjacent to an AgrA binding site (PMID: 28955870 and 21908676), so there is a possibility that this mutation affects AgrA binding. Therefore, pDB59 was not a good choice for this study because the MN8 P2-P3 region does not reflect the agr systems of the selected strains.

We thank the reviewer for this detailed and technically insightful comment revealing that the MN8-derived P2–P3 region in pDB59 contains sequence differences, including the mismatch in the P3 region spanning AgrA and polymerase binding site that could negatively influence RNA polymerase and/or AgrA in the strains used in the initial submission. We appreciate the reviewer pointing out the exact position of the MN8 deviation and its potential implications for promoter recognition.
To address this concern, all main-text experiments in the revised manuscript were repeated using a newly constructed P3–*gfp* reporter plasmid in which: i) the P3 promoter exactly matches the chromosomal P3 sequence of each congenic strain, i.e., AgrA and polymerase binding sites are sequence-perfect for each respective strain; and ii) only P3, and not P2, is included (avoiding the dual-promoter architecture of pDB59). This design ensures that promoter recognition and AgrA binding on the plasmid reporter reflect the chromosomal context without introducing MN8-specific mismatches.
The original pDB59-based data are now used in the Supplement for comparison. We explicitly acknowledge the limitations of pDB59 in the Methods (L514): "Of note, our constructed P3-*gfp* plasmids only contain the P3 promoter while the pDB59 carries both P2 and P3 promoters and was optimized for the MN8 strain[7] which may impact AgrA binding efficiency and increase required phosphorylated AgrA for P3 activation."
Importantly, despite the revealed limitations of pDB59, the key biological patterns obtained with native-*agr* strains carrying pDB59 closely match those obtained with the new, fully sequence-matched reporters in congenic-*agr* strains. This agreement suggests that, while pDB59 is not ideal for strain-specific promoter comparisons, its use did not alter the central conclusions of our study. We hope that the revised experimental design and the explicit clarification now included in the manuscript address the reviewer's concern.

3. P3 L91 and P11 L318: Plasmid pDB59 is a multicopy plasmid. This is highly problematic to this study for three reasons. The first is that AgrA is known to have a higher affinity for the P2 promoter. Plasmid pDB59 is derived from pDB39, which is a shuttle vector that uses a pC194 replicon for S. aureus replication. Normally, pC194 will average around 100 copies per cell. This means that phosphorylated AgrA will need to reach levels high enough to saturate the many copies of P2 before it starts to bind to and activate a P3. This is very different than how it works with the native P2-P3.

We thank the reviewer for this important and technically detailed comment. We fully agree that the multicopy nature of pDB59, and more generally of pC194-based replicons, can influence *agr*-reporter dynamics by altering the balance of available promoter sites for phosphorylated AgrA. In particular, as the reviewer notes, the presence of the P2 promoter on pDB59 could sequester AgrA and raise the threshold required for P3 activation.

To address this, all main-text experiments were repeated with newly constructed P3-gfp reporter plasmids as noted in the previous comment: i) the P3 promoter exactly matches the chromosomal P3 sequence of each congenic strain, i.e., AgrA and polymerase binding sites are sequence-perfect for each respective strain; and ii) only P3, and not P2, is included (avoiding the dual-promoter architecture of pDB59). This design eliminates the major concern raised by the reviewer, AgrA sequestration by plasmid-borne P2.

We acknowledge that our new P3-*gfp* plasmids still carry the pC194 origin and are therefore multicopy, although the removal of P2 eliminates the most problematic confounder. To evaluate whether multicopy P3 alone distorts *agr*-readouts, we performed independent qPCR measurements of chromosomal *agrA*, RNAIII, *psmβ2*, and plasmid-encoded *gfp* in early exponential-phase cultures with and without AIP stimulation. The transcriptional correlations between *gfp* and the chromosomal *agr*-regulated genes (*agrA*: 0.51; RNAIII: 1.3; *psmβ2*: 1.3 on a log–log scale; Supplementary Fig. 6) demonstrate that *gfp* expression faithfully tracks *agr*-activation, especially for directly P3-controlled RNAIII and *psmβ2*. This strongly suggests that multicopy P3 does not uncouple plasmid-based readouts from chromosomal *agr*-regulation in our experimental conditions.

Importantly, the qualitative and quantitative patterns obtained with the new reporter plasmids closely match those obtained using pDB59 in native strains, indicating that the presence of P2 on pDB59 did not materially alter the central biological conclusions.

Nevertheless, we agree that the plasmid-based reporter does not fully replicate the native single-copy P2–P3 architecture. We now explicitly discuss this limitation and the implications for AgrA availability and activation thresholds in the revised Discussion (section starting at L439). Future studies using chromosomally integrated reporters or rapidly degradable fluorophores will be needed to fully eliminate plasmid-related confounders.

We hope that these additions and clarifications satisfactorily address the reviewer's concern and demonstrate the robustness of our revised experimental design.

The second is that multicopy plasmids vary in copy number from cell to cell. Unfortunately, this is where a single-cell study works against the authors because it is not possible to control the copy number precisely for any given cell. Numerous factors, such as the precise concentration of antibiotics in the media, will influence the precise plasmid copy number. Naturally, P3 activation in any given cell will be affected by plasmid copy number.

We thank the reviewer for raising this important point. We agree that plasmid copy number cannot be controlled at the single-cell level and may vary between cells. However, in our microfluidic setups the environmental conditions (including nutrient and antibiotic concentrations) are held at steady state, which should reduce copy-number fluctuations across the population. Especially the small mother-machine chambers should have uniform chemical conditions. In the connected-chamber experiments, where chemical gradients may develop, we analyzed aggregated single-cell fluorescence intensities onto chamber-level behavior and binarized *agr*-activation (*agr*-OFF vs *agr*-ON). This reduces the influence of cell-to-cell variation in plasmid number on our conclusions for these experiments.

In the mother-machine experiments, although individual plasmid copy numbers cannot be measured, the extremely large number of quantified cells minimizes the influence of rare outliers. The continuous GFP distributions of *agr*-ON cells, which likely reflect multiple biological as well technical sources of variation, were not used for direct quantitative inference apart from visualization, especially the illustration of bimodal *agr*-activity distribution. All conclusions, including the bimodal *agr*-activation, were drawn from or emphasized by binarized *agr*-activation states, for which the *agr*-OFF population is sharply defined. We

therefore chose not to analyze the width of the *agr*-ON fluorescence distribution, as plasmid copy-number differences cannot be excluded as a contributing factor. The characterization of the continuum of *agr*-activation remains an interesting question for future research.

To acknowledge this limitation, we have added a statement to the Discussion (L447+) explicitly noting that multicopy plasmids introduce uncontrollable variation in promoter dosage and may contribute to fluorescence variability.

The third is that once P3 is activated, it is one of the strongest promoters in S. aureus. For this reason, the agr locus cannot be stably maintained on an episomal plasmid, even on ones that are extremely low copy, because mutations are strongly selected owing to the extremely high metabolic burden. Likewise, it is well known among groups that work on agr that P3 fusions on multicopy plasmids are highly unstable and rapidly accumulate mutations in P3. This has even been documented a few times (PMID: 25192956). Admittedly, the MN8 P3 may not have this problem because of the -35 element mutation, though it is likely that it also has this issue.

We thank the reviewer for raising this critical point. We fully agree that P3 is one of the strongest promoters in *S. aureus* and that maintaining P3-driven reporters on multicopy plasmids can impose strong selective pressure for promoter-attenuating mutations. We therefore examined this issue carefully, considering further the cited reference (PMID: 25192956). While this paper demonstrates that expression from multicopy plasmids is highly variable, with *agr*-P3-blaZ fusions on the episomal pJC1280 vector displaying broad activity ranges whereas chromosomally integrated fusions produce uniform expression, it does not directly report P3 sequence mutations or *agr*-specific promoter instability. We presume the citation was intended to highlight expression variability of multicopy P3 reporters, which indeed aligns with the broader field experience that high-strength promoters on multicopy plasmids are prone to instability. We agree that this represents a relevant limitation.
Several features of our experimental design, however, make it unlikely that promoter mutation substantially altered our main conclusions:

1. Short activation windows and constant nutrient supply.
   In the mother-machine, *agr*-activation occurs only transiently and under nutrient-rich, steady-state conditions, markedly reducing the selective pressure typically associated with prolonged high-level P3 activity usually linked with nutrient stress. Only one strain (502a-II) showed a strong AIP-dependent growth impairment, underscoring that genetic-background-specific interaction effect with *agr*-activation, and not promoter instability, as the likely driver of stress in our system.
2. *Agr* OFF-to-ON switching argues against irreversible plasmid loss.
   We frequently observed non-fluorescent cells dividing producing fluorescent daughter cells. This behavior is incompatible with promoter-inactivating mutations or plasmid loss, and indicates that the GFP reporter remained functional during the timescales of our experiments.
3. Strong qPCR correlation with chromosomal *agr*-output.
   In early exponential cultures obtained by triple dilution-growth cycles starting from an overnight culture, *gfp* expression correlated closely with chromosomal *agrA*, RNAIII, and *psmβ2* expression. Such parallel transcriptional responses would be unlikely if promoter mutations had accumulated either in the preceding overnight culture in which nutrient depleted and the *agr*-system autoinduced or in the exponential

cultures. We acknowledge that population-level qPCR cannot exclude rare, mutated subpopulations, but it supports overall reporter integrity.

4. Concordance between old and new reporter systems.
   The redesigned P2-free, sequence-matched P3-*gfp* plasmids reproduced the key qualitative activation behaviors observed with pDB59, despite independent plasmid constructions and alternative genetic background. This reproducibility indicates that, although plasmid instability is a valid theoretical concern, any promoter variants that may have arisen did not systematically distort the activation patterns central to our conclusions.
   We nevertheless fully acknowledge that multicopy plasmids place P3 in a non-physiological genetic context and that promoter mutation remains an intrinsic risk of plasmid-based reporters. We now explicitly discuss this limitation in the revised manuscript (section beginning at L447).

To avoid the aforementioned issues, P3 fusions should be maintained in single-copy integration vectors. The P3 promoter, in a single copy, is strong enough if that is a concern.

We thank the reviewer for this important recommendation. We agree that chromosomal, single-copy integration of a P3 reporter is the most rigorous and physiologically accurate way to quantify P3 activity, and we explicitly acknowledge this in the revised Discussion. Integrating P3 reporters would indeed eliminate plasmid copy-number variability, reduce metabolic burden, and prevent selective pressures that can arise from maintaining strong promoters on episomal replicons.
In the context of the present study, however, several layers of evidence indicate that our revised reporter system provides a reliable and interpretable readout of *agr*-activation, despite the inherent limitations of multicopy plasmids:

1. We eliminated the major mechanistic confounder identified by the reviewer by constructing new P3-*gfp* reporters containing only the precise chromosomal P3 sequence for each congenic strain and removing P2 entirely, thereby avoiding AgrA sequestration.
2. We observe *agr* OFF→ON switching of individual cells and across division events, which is incompatible with stable promoter inactivation or plasmid loss, indicating that promoter function is maintained over the course of our experiments.
3. Independent qPCR validation demonstrated strong correlation between *gfp* and chromosomal *agrA*, RNAIII, and *psmβ2* expression, suggesting that plasmid-encoded P3 activity tracks chromosomal *agr*-activation at the transcript level.
4. Biological reproducibility across reporter architectures and strain backgrounds is high: the new chromosomally matched, P2-free reporters in congenic strains produced the same qualitative activation hierarchy and heterologous inhibition patterns as the native strains carrying pDB59.
5. The cultivation regime minimizes selection for promoter mutants. In the mother-machine, *agr*-activation is transient, nutrient supply is constant, and high P3 activity does not persist across many generations, conditions under which selection for attenuating mutations is expected to be minimal.

We therefore believe that, while single-copy integration would certainly be the ideal long-term solution, the combination of redesigned plasmids, extensive validation, and

consistent biological behavior across strains makes the current dataset robust and fit for purpose. To ensure transparency, we now explicitly state these limitations and future directions in the Discussion (section beginning at L447), where we highlight chromosomal reporters and low-copy systems as a natural next step to further refine agr quantification.

4. P11 L317: Four agr-types were used for this study: LAC, agr-I, ST8; 502a, agr-II; MW2, agr-III; and MNTG, agr-IV. The issue that I see here is that different agr types are compared but the strain backgrounds are not isogenic. It is more accurate to say AIP sensitivity among four strains was compared, rather than four agr-types.
There are established experimental systems that control for the strain background when comparing different agr-types (PMID: 22467783). The four agr-types can be individually inserted into a single strain background with the native agr locus deleted. This should have been done here. Otherwise, there are too many variables that come with different strain backgrounds.

We thank the reviewer for raising this important point. We fully agree that comparing *agr*-types across unrelated genetic backgrounds can confound interpretation, as genotype-specific regulatory circuits may mask or amplify *agr*-type-dependent behaviors. This concern was also raised by other reviewers, and in response we obtained and employed the congenic-*agr* strain set described in the mentioned publication by Novick's group, PMID: 22467783 (Newman background; native-*agr* deleted and replaced with *agr*-I–IV), kindly provided by Bo Shopsin. All main experiments in the revised manuscript were repeated using this clean genetic system, together with sequence-matched P3-*gfp* reporters.
Using these congenic strains allowed us to assess *agr*-type effects independent of genetic background, and the key findings from the initial submission were confirmed and strengthened:

- *agr*-III displays markedly reduced AIP sensitivity and delayed *agr*-activation
- *agr*-IV has elevated unstimulated basal *agr*-activity
- bimodal *agr*-activation is an intrinsic feature of the system and may be explained by simultaneous existence of a majority of *agr*-ON bacteria with both low frequency of *agr*-OFF and ON-to-OFF switching subpopulations
- *agr*-I can be inhibited by AIP-IV at the single-cell level

We now treat the native strain dataset of the initial submission as a biologically valuable complementary comparison, highlighting how *agr*-signaling manifests in native genetic backgrounds while distinguishing these results from the *agr*-type-isolated insights obtained with the congenic strains. The strong concordance between the two datasets, despite their different architectures, reinforces the robustness of our conclusions. These comparisons and their respective limitations are now explicitly discussed throughout the Results and Discussion sections.
However, the connected-chamber interaction assays became more deterministic in the congenic background, consistent with intrinsic *agr*-type differences. By contrast, the native strain pairings included already in the initial submission showed broader outcome variability, illustrating the additional layer of complexity introduced by differing genetic backgrounds.

We hope these revisions demonstrate that we have addressed the reviewer's concern comprehensively: the revised manuscript provides both a clean *agr*-type comparison in a

congenic background and a contextual view of how *agr*-behavior manifests across diverse *S. aureus* lineages.

Less substantial remarks:

1. Figure 1,3: Please give a brief description of controls.

We thank the reviewer for this comment. We have now added a concise explanation of the control conditions in the Results, Methods, and the figure legends. Specifically:

- Results (L116): "In the absence of synthetic AIP (Control; 2.5% Phosphate-Buffered-Saline, PBS), NM-I, NM-II, and NM-III showed no detectable *agr*-activation, indicating that self-produced AIP remained below their P3 activation thresholds (Fig. 1d)."
- Methods (L502): "Control samples received 2.5% PBS instead of AIP, matching the buffer concentration used for AIP dilution."
- Figure 1 legend: "Control replicates received 2.5% PBS instead of AIP."

In brief, the control microfluidic experiments were imaged identically to AIP-exposed experiments, except that media was switched to media containing the equivalent volume of PBS alone (used buffer to dilute synthetic AIPs), ensuring that any observed changes arise from AIP and not from the buffer or slightly reduced nutrient concentrations.

2. Controls such as agr deletions would be good to add.

We thank the reviewer for this suggestion. We considered the use of an *agr*-deletion strain as a control. The *Δagr* mutant used as the parental background for constructing the congenic-*agr* strains (PMID: 22467783) is genetically incapable of producing their own AIPs nor phosphorylated AgrA. Consistent with this, the strain shows no detectable P3-GFP activation in bulk plate-reader assays, even under such conditions that allow endogenous AIP to accumulate or when provided with synthetic AIP (data reported below). Given this genetic constraint and the absence of P3 activation in bulk assays, we would expect the *Δagr* strain to remain *agr*-OFF under all microfluidic conditions as well, independent of AIP exposure. As such, microfluidic experiments with this background would not provide additional information beyond confirming its constitutive lack of *agr*-activation.

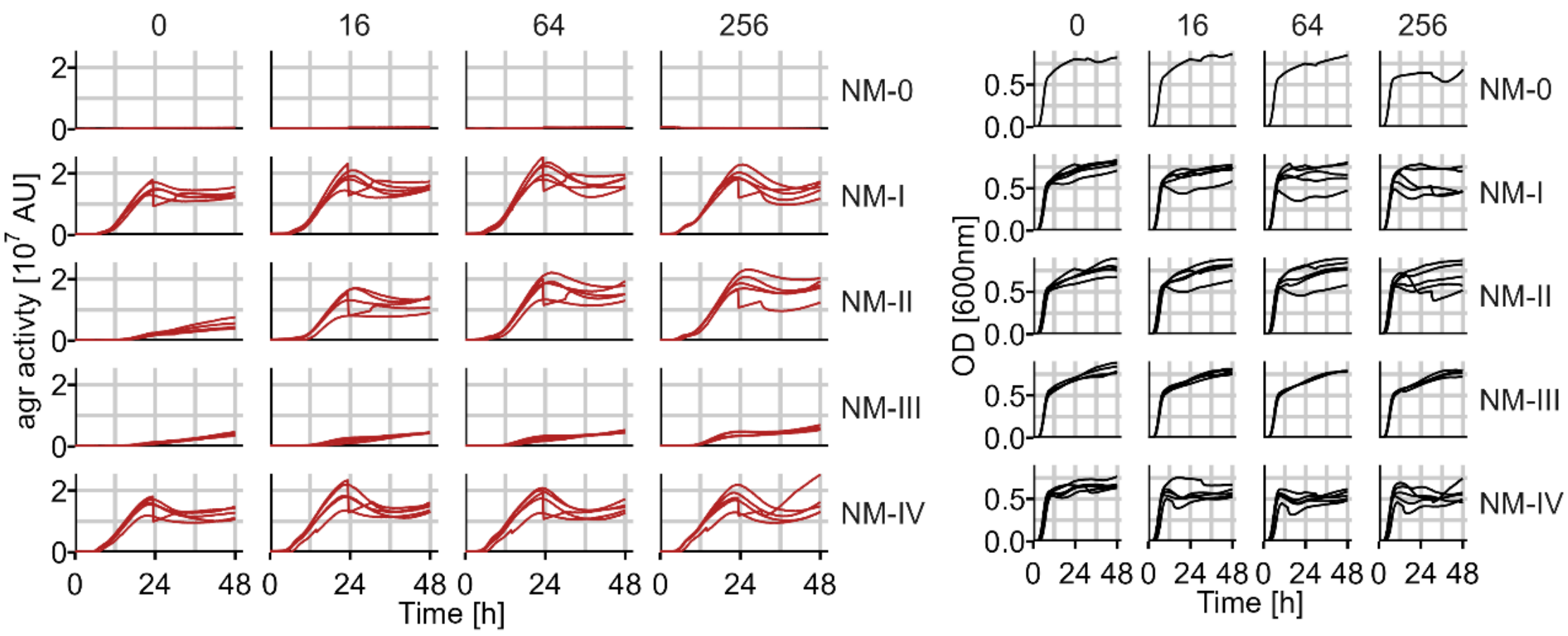

Figure: 96-well plates were inoculated at an OD_{600} of 0.0025 derived by PBS washing and diluting an overnight culture. The plate was incubated with intermittent shaking at 37°C and GFP fluorescence as

well as OD recorded every 15min for 48h. AIP concentrations provided at plate-reader inoculation are annotated above columns. NM-0 is the *Δagr* mutant, provided with AIP-I (chosen as the parental strain Newman is *agr*-type I). Only one replicate was performed for this strain. The data for the other strains is the same as shown in Supplementary Fig. 1.

3. P15 L443: "To assess baseline gene expression profiles of all four strains, overnight cultures grown at 37° C in TSB with Cm10 and 0.01% Tween20 were diluted 1:20 in fresh medium and regrown for 1.5 h."
Typically, a single subculture of an overnight does not result in true baseline agr activity. The overnight culture is full of AIP and a 1:20 dilution still has sufficient AIP to activate QS. Generally, subculturing a second or third time with exponentially growing cultures will achieve a baseline.

We thank the reviewer for highlighting this important technical point. We agree that a single 1:20 dilution of an overnight culture is insufficient to reset *agr*-activity, as residual AIP can remain at concentrations capable of sustaining QS activation. As suggested, we have now implemented a protocol including triple subculturing with PBS washes to ensure complete AIP removal and full *agr*-reset for all baseline transcriptomic measurements and the newly added qPCR validations.

Specifically, overnight cultures were washed twice in PBS, diluted 1:20 into fresh TSB, grown for 1.5 h, and this wash–dilute–regrow cycle was repeated a total of three times before sampling. RNA-seq was then performed on the third 1.5 h subculture of the congenic *agr*-I–IV Newman strains. These revised experiments show no significant differences in baseline gene expression between *agr*-types (Supplementary Fig. 5). These results confirmed that *agr* is fully inactive under early exponential conditions and there are no baseline expression differences that may explain AIP sensitivity differences observed in mother-machine.
We appreciate the reviewer's suggestion; it substantially improved the rigor of the baseline transcriptomic comparisons, and only the revised data are now included in the manuscript. The previous RNA -seq data has been removed.
