# Supplementary material for: Single-cell approach dissecting agr quorum sensing dynamics in Staphylococcus aureus
Source: Nat Commun. 2026 May 20;17:8139. doi: 10.1038/s41467-026-73552-9 (PMC13458059; doi:10.1038/s41467-026-73552-9)
Supplement: Supplementary file 3 [file 41467_2026_73552_MOESM1_ESM.pdf]

Supplementary Information

Single-cell Approach Dissecting *agr* Quorum Sensing Dynamics in *Staphylococcus aureus*

Julian Bär [1], Mariane Pivard [1], Samuel G. V. Charlton [2], Andrew G. Kempchinsky [1], Alejandro Gómez-Mejia [1], Giovanni Stefano Ugolini [2], Srikanth Mairpady Shambat [1], Eleonora Secchi [2], Annelies S. Zinkernagel [1,*]

1 Department of Infectious Diseases and Hospital Epidemiology, University Hospital Zürich, University of Zürich, Zürich, Switzerland
2 Department of Civil, Environmental and Geomatic Engineering, ETH Zurich, Zurich, Switzerland
* Corresponding author: Annelies Zinkernagel, Rämistrasse 100, 8091 Zürich, Switzerland.
E-mail address: annelies.zinkernagel@usz.ch

Supplementary Figures **Page 2**

Supplementary Tables **Page 19**

Supplementary Figures

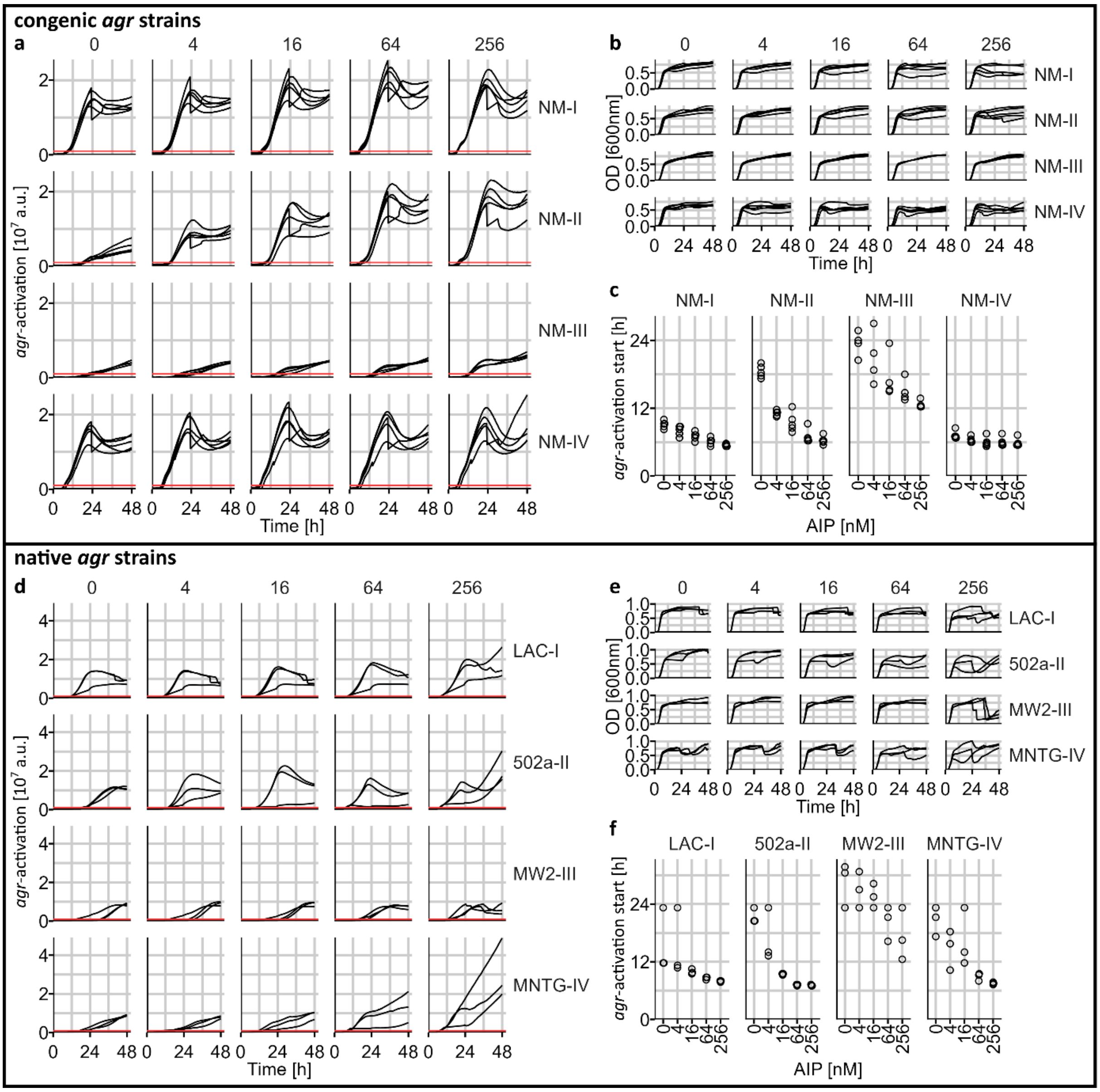

Supplementary Figure 1: Plate-reader based *agr*-activation of the congenic and native *agr*-strains. a. *Agr*-activation (GFP fluorescence in arbitrary units (a.u.)) was measured over 48 h in a plate-reader with different concentrations of synthetic, homologous AIP provided at the beginning of growth (inoculation at OD_{600} of 0.0025 in TSB + Cm10). Experiments were repeated 4-5 times. The red line indicates the threshold ($2*10^6$ AU) used for *agr*-activation start calculation shown in panel c. **b.** The optical density (OD_{600}) was measured simultaneously. **c.** *agr*-activation start defined as the time when the GFP signal surpassed the threshold shown in panel a. **d-f** show the same as a-c but for the native *agr*-strains.

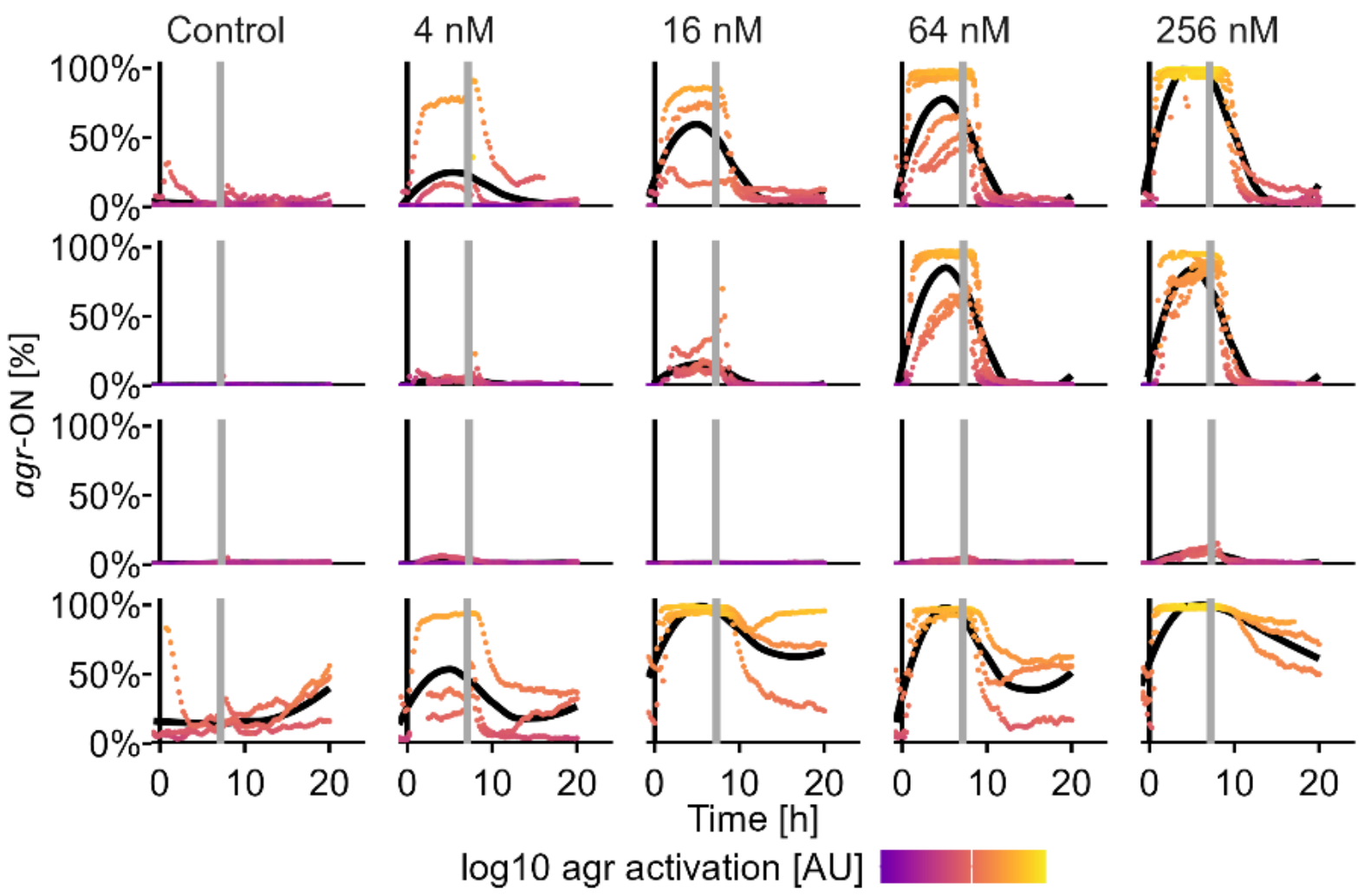

Supplementary Figure 2: Proportion of *agr*-ON cells of the congenic-*agr* strains. Percentage of *agr*-ON bacteria (GFP signal per cell > 100 arbitrary units) calculated per biological replicate of the mother-machine experiments shown in Fig. 1. AIP removal is indicated with a vertical gray line and synthetic AIP concentration annotated on top of the panels. Color indicates $\log_{10}$ *agr* activation (GFP signal). As NM-III cells respond very weakly to synthetic, homologous AIP, the threshold of 100 AU is almost never reached. Black lines show LOESS smoothing.

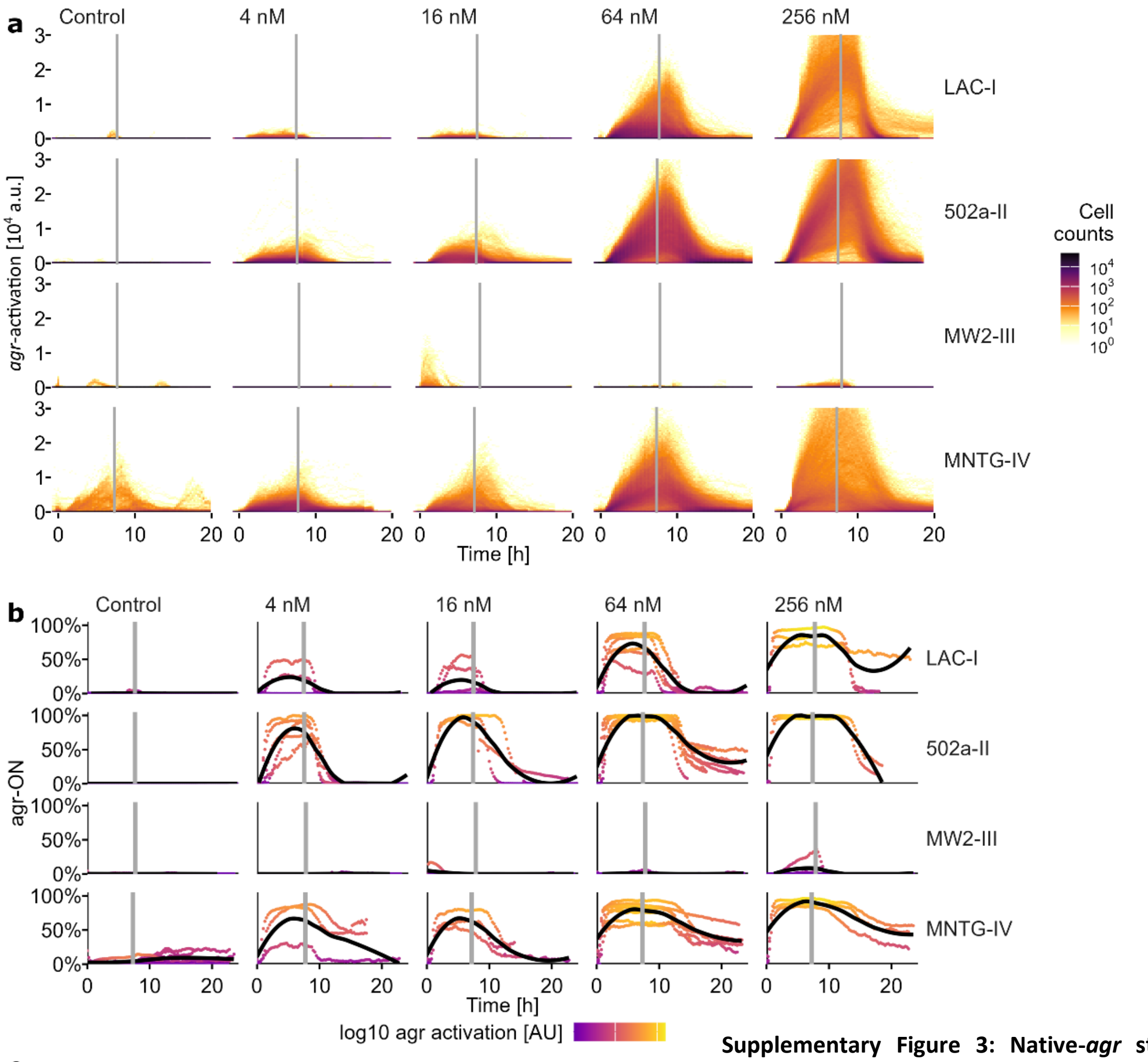

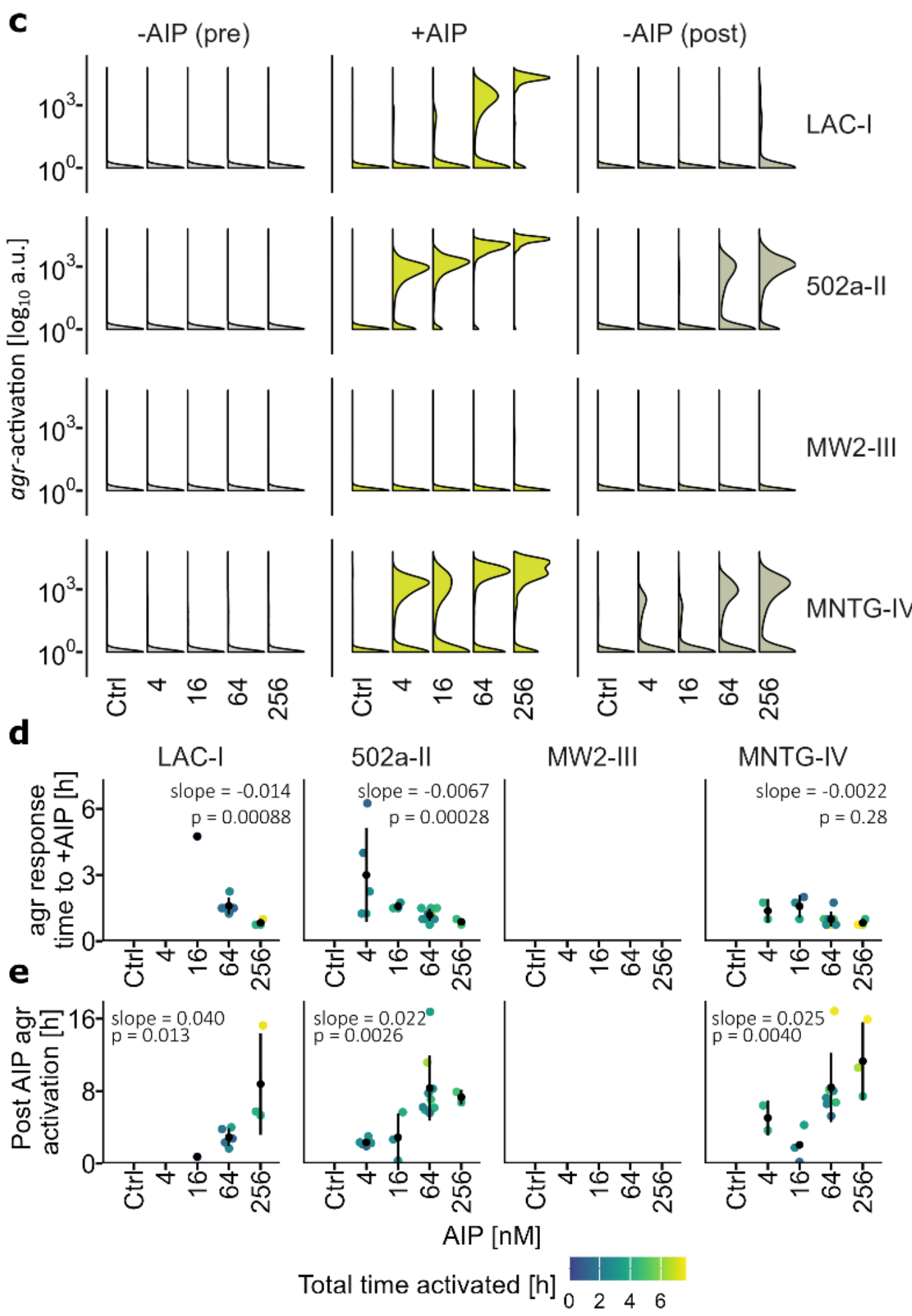

Supplementary Figure 3: Native-*agr* strains show comparable AIP sensitivity patterns as the congenic-*agr* strains. a. Single-cell *agr*-activation (GFP intensity in arbitrary units, a.u.) during synthetic, homologous AIP exposure (concentration indicated above each column) and after AIP removal (gray lines, plus/minus symbols). Each row represents one *agr*-type. Dot color reflects $\log_{10}$ cell counts. On average, 1'704'038 cells per condition were analyzed (Supplementary Table 2). **b.** Proportion of *agr*-ON cells (YFP signal per cell > 100 arbitrary units calculated per biological replicate (dots); Black lines show LOESS smoothing. AIP removal is indicated with a vertical gray line and exogenous AIP concentration annotated at top of panels. **c** Histograms of $\log_{10}$ *agr*-activation (subset of data shown in a) during defined intervals: -AIP (pre) 2 h before exposure (2 to 0 h); +AIP, final 2 h of exposure (5.5 to 7.5 h); -AIP (post), 3-5 h after AIP removal (10.5 to 12.5 h). **d-e.** Each dot represents a biological replicate (N = 3-7 independent mother-machine experiments), details in Supplementary Table 2), colored by total *agr*-activation time; black dots/bars show mean ± SD. Statistical significance was assessed using linear regression on $\log_2$ AIP concentration (slope and p-value annotated) with estimated marginal means *post-hoc* tests. **d.** Response time: time until >50% of cells became *agr*-ON after AIP addition. **e.** Post-AIP activation time: time until <50% of cells remained *agr*-ON after AIP removal. Only conditions reaching the 50% threshold are shown; no timing was computed when thresholds were not met (e.g., only one replicate for LAC-I at 16 nM).

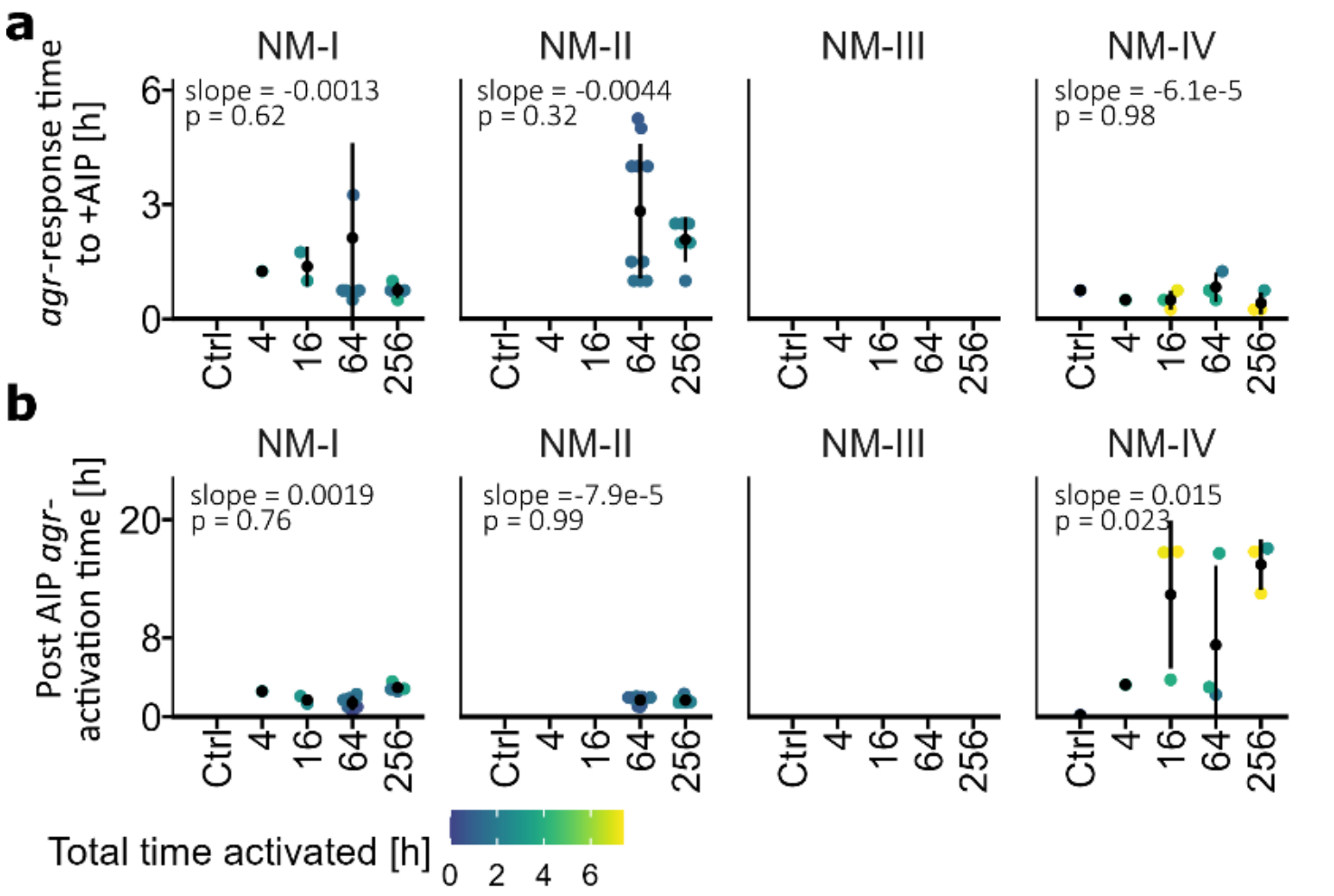

Supplementary Figure 4: *agr*-response time of congenic-*agr* strains. Each dot represents a biological replicate (N = 3-7, details in Supplementary Table 1), colored by total *agr*-activation time; black dots/bars show mean ± SD. Statistical significance was assessed using linear regression on $\log_2$ AIP concentration with estimated marginal means *post-hoc* tests (slope and p-value annotated). **a.** Response time: time until >50% of cells became *agr*-ON after AIP addition. **b.** Post-AIP activation time: time until <50% of cells remained *agr*-ON after AIP removal. Only conditions reaching the 50% threshold are shown; no timing was computed when thresholds were not met (e.g., only one replicate for NM-I at 4 nM).

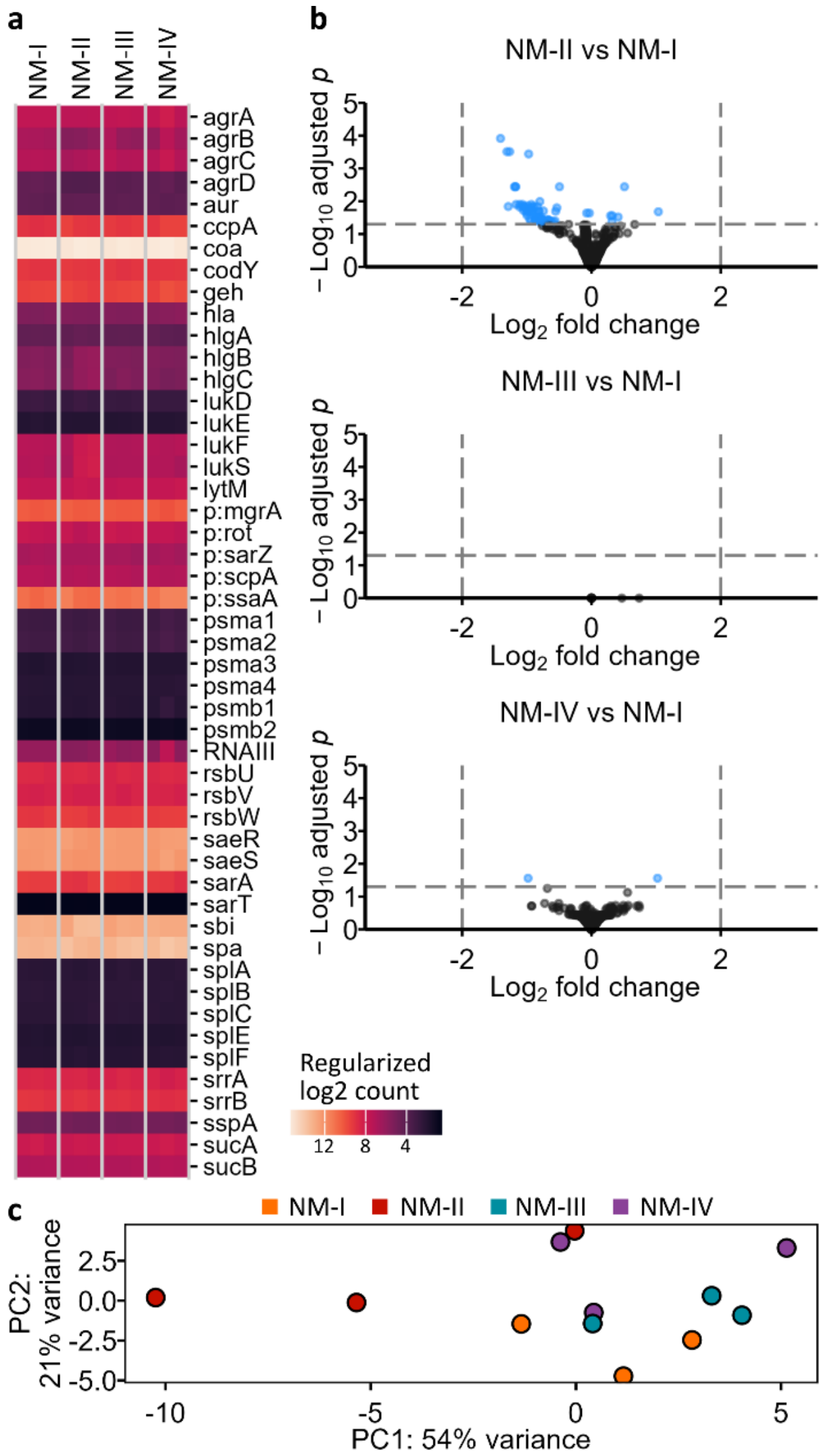

Supplementary Figure 5: No differences in baseline expression between the congenic-*agr* strains. RNA sequencing of exponentially growing cultures (1.5 h TSB) without AIP stimulation was performed to quantify gene expression profiles of the four congenic-*agr* strains to investigate if these could explain AIP sensitivity differences observed in the mother-machine experiments. N = 3 biological replicates from independent cultures, performed on separate days. **a.** Heatmap of rlog transformed RNA counts of the four genes of the RNAII operon, regulators known to interact with the *agr*-system and downstream affected genes. Columns represent *agr*-type and biological replicates. Rows are sorted alphabetically. Gene names starting with "p:" were not present in the available Newman annotation file and originate from the pan genome annotation of AureoWiki. **b.** Volcano plots of DESeq2 based pairwise comparisons of any *agr*-type with NM-I. **c.** Principal component (PC) analysis of regularized $\log_2$ (rlog) transformed RNA sequencing counts. Dots represent three independent biological replicates. PC1 and PC2 explained 54% and 21% of the variance, respectively, and axis ratio is scaled accordingly.

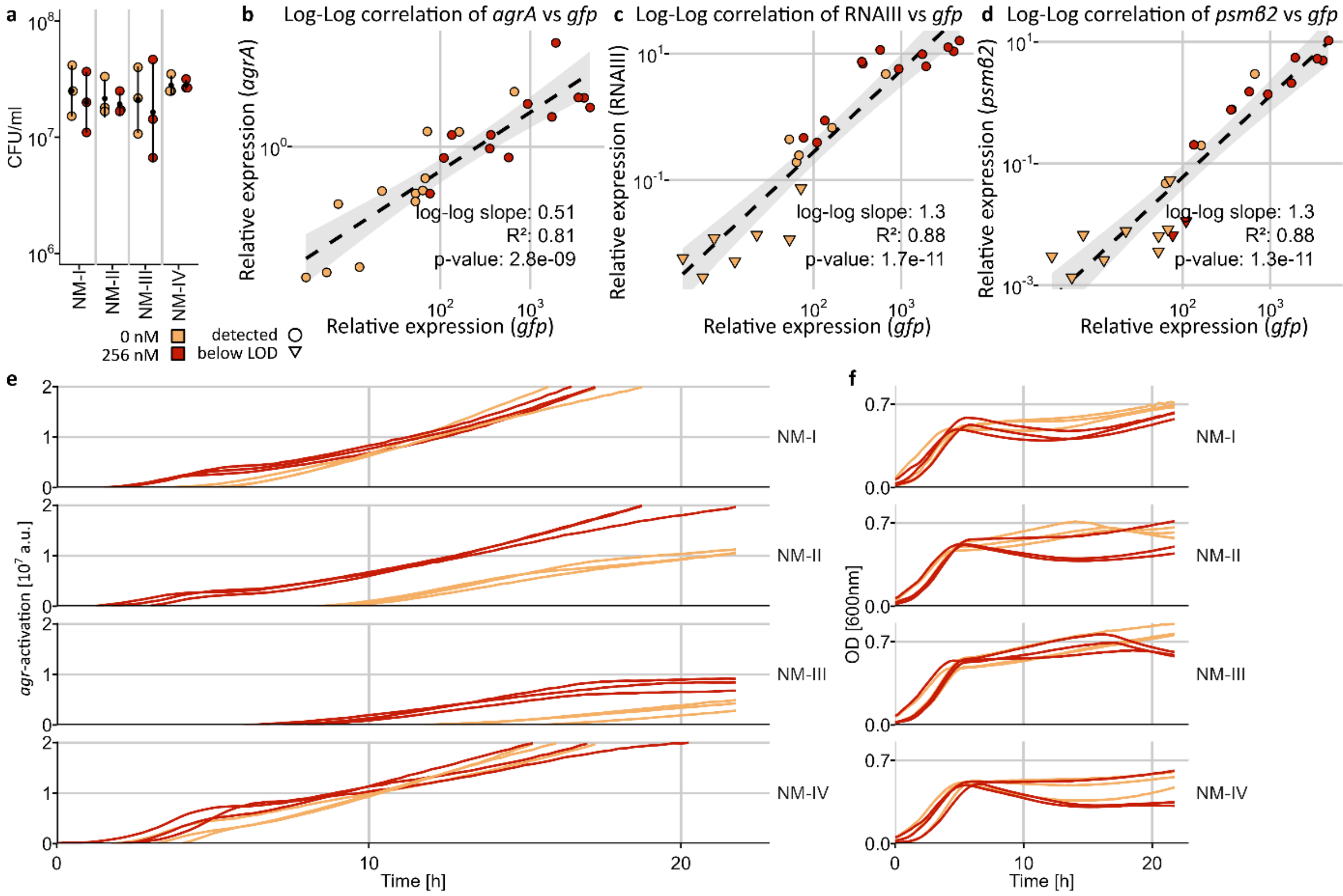

Supplementary Fig. 6: Additional data for Fig.1g: qPCR of exponentially growing cultures (1.5h TSB culture) with (red) and without (yellow) AIP stimulation. Experiments were performed in biological triplicates from independent cultures, performed on separate days. **a.** Corresponding bacterial density (CFU/ml), demonstrating the absence of growth differences. Colored dots show replicates, bars indicate mean ± SD. **b-d.** Correlation plots comparing qPCR-based relative expression of the genes *agrA*, RNAIII and *psmβ2* with *gfp*. Same data as shown in Fig.1g. Dots and triangles are individual replicates, triangles indicate samples below limit of detection (LOD; Cq = 30), dashed lines show log-log linear regression and shaded area indicate SD. **ef.** After RNA isolation for qPCR, cultures were transferred to a plate-reader for GFP fluorescence and optical density measurements. **e.** GFP-based *agr*-activation (arbitrary units, a.u.) during the first 24 h of growth (curves clipped if above y-axis limit). Color indicates AIP exposure condition. **f.** Optical density measured simultaneously.

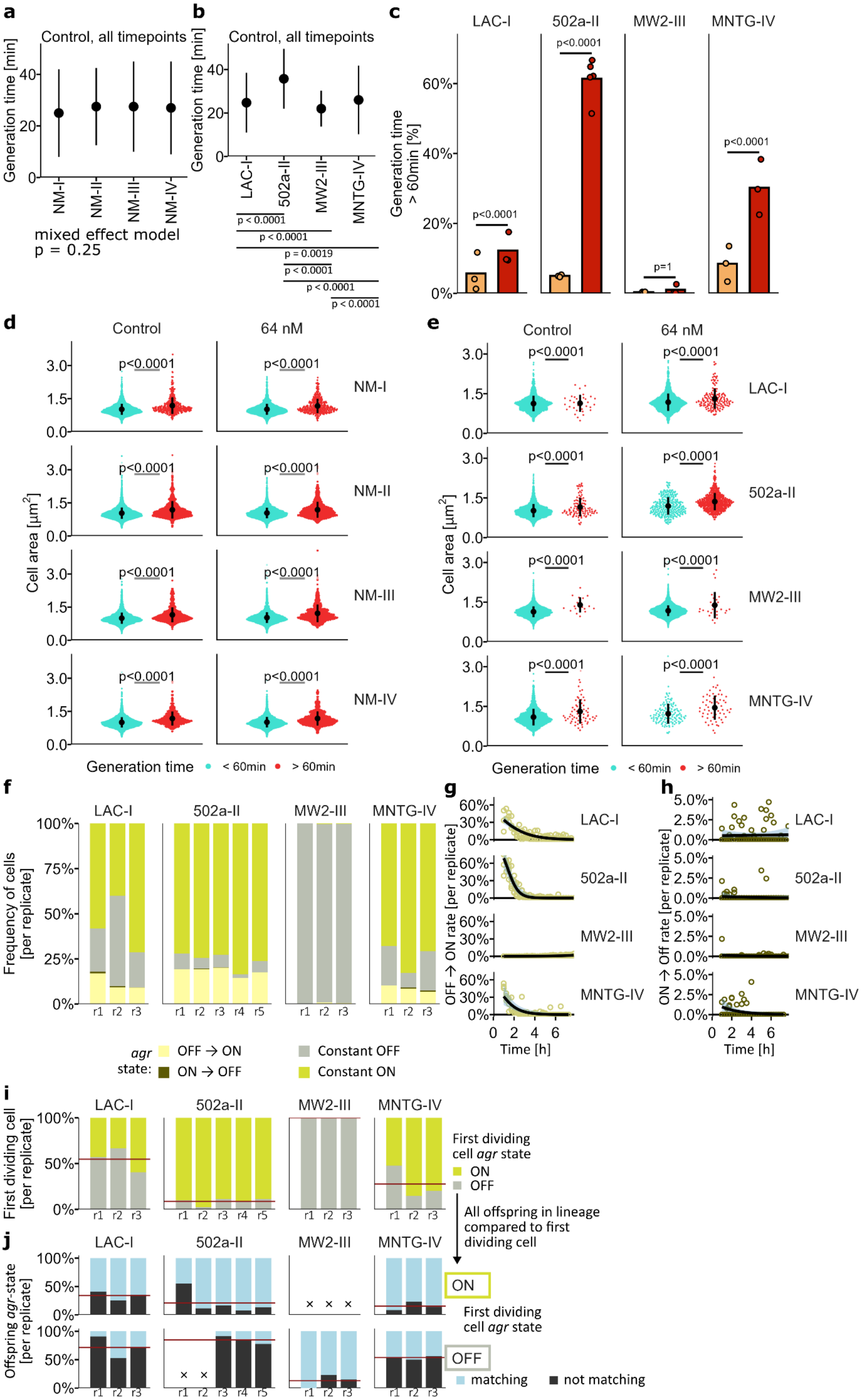

a
Control, all timepoints
Generation time [min]
0
20
40
NM-I
NM-II
NM-III
NM-IV
mixed effect model
p = 0.25
b
Control, all timepoints
Generation time [min]
0
20
40
LAC-I
502a-II
MW2-III
MNTG-IV
p < 0.0001
p < 0.0001
p = 0.0019
p < 0.0001
p < 0.0001
p < 0.0001
c
LAC-I
502a-II
MW2-III
MNTG-IV
Generation time > 60min [%]
0%
20%
40%
60%
p<0.0001
p<0.0001
p=1
p<0.0001
d
Control
64 nM
Cell area [µm²]
0.0
1.5
3.0
p<0.0001
NM-I
NM-II
NM-III
NM-IV
Generation time
< 60min
> 60min
e
Control
64 nM
Cell area [µm²]
0.0
1.5
3.0
p<0.0001
LAC-I
502a-II
MW2-III
MNTG-IV
Generation time
< 60min
> 60min
f
LAC-I
502a-II
MW2-III
MNTG-IV
Frequency of cells [per replicate]
0%
25%
50%
75%
100%
r1 r2 r3 r4 r5
agr state:
OFF → ON
ON → OFF
Constant OFF
Constant ON
g
OFF → ON rate [per replicate]
0%
30%
60%
LAC-I
502a-II
MW2-III
MNTG-IV
0 2 4 6
Time [h]
h
ON → Off rate [per replicate]
0.0%
2.5%
5.0%
LAC-I
502a-II
MW2-III
MNTG-IV
0 2 4 6
Time [h]
i
LAC-I
502a-II
MW2-III
MNTG-IV
First dividing cell [per replicate]
0%
50%
100%
r1 r2 r3 r4 r5
First dividing cell agr state
ON
OFF
All offspring in lineage compared to first dividing cell
j
LAC-I
502a-II
MW2-III
MNTG-IV
Offspring agr-state [per replicate]
0%
50%
100%
r1 r2 r3 r4 r5
ON
First dividing cell agr state
OFF
matching
not matching

Supplementary Figure 7: Generation time, single-cell *agr*-dynamics and cell size. a-b. Median ± interquartile range of generation time calculated over all control (0 nM AIP) experiments. **a.** shows data for congenic-*agr* strains and **b.** for the native strains. Statistical significance was assessed with a mixed effects model on $\log_{10}$ transformed generation time followed by estimated marginal means pairwise comparisons. As the overall model was non-significant for congenic strains, no pairwise comparisons were computed. Number of cells and replicates are listed in Supplementary Table 1 and 2. **c.** The proportion of native *agr*-type bacteria with prolonged generation time during exposure phase was calculated per replicate based on the threshold of 60 min. Bars show means; dots show biological replicate values (N =3-5, details in Supplementary Table 2). P-values are based on a generalized linear mixed-effects model with estimated marginal means *post hoc* tests. **c** and **d.** Maximal cell area during AIP exposure, separated by the generation time threshold of 60 min. Black dot and bar show mean ± SD. P-value based on linear mixed effect model on $\log_{10}$ transformed cell area and estimated marginal means *post-hoc* test (number of cells and replicates listed in Supplementary Table 1 and 2). Colors indicate generation time state. **d.** for the congenic Newman strains and **e.** for the native-*agr* strains. **f.** For the native-*agr* strains, single-cell *agr*-dynamics (per cell from division to division) were classified as constant ON, constant OFF, or switching (OFF→ON, ON→OFF) using a 100 arbitrary unit fluorescence threshold. All cells alive during exposure phase were considered. One stacked bar represents one biological replicate. **g-h.** For the native-*agr* strains, time-dependent analysis of switching events from the same dataset as in panel e. **g**, OFF→ON transitions; **h**, ON→OFF transitions. The x-axis indicates the time of cell division (end of cell cycle); black lines represent binomial regression fits (N = 3-5, details in Supplementary Table 2). **i.** *agr*-state of first-dividing cells per replicate of native-*agr* strains (stacked bars). Red horizontal lines indicate average per *agr*-type. **j.** Proportion of offspring per lineage retaining the same *agr*-state as the first-dividing cell of native-*agr* strains (blue, matching first-dividing cell; black, not matching first-dividing cell), shown separately for ON (top, yellow in i) and OFF (bottom, gray in i) first-dividing cells and by replicate. Red horizontal lines indicate average per *agr*-type and first dividing cell state. Stars indicate replicates where no first-dividing cells, or fewer than six, were present in a given *agr*-state (see h); therefore, offspring stability could not be assessed for that state.

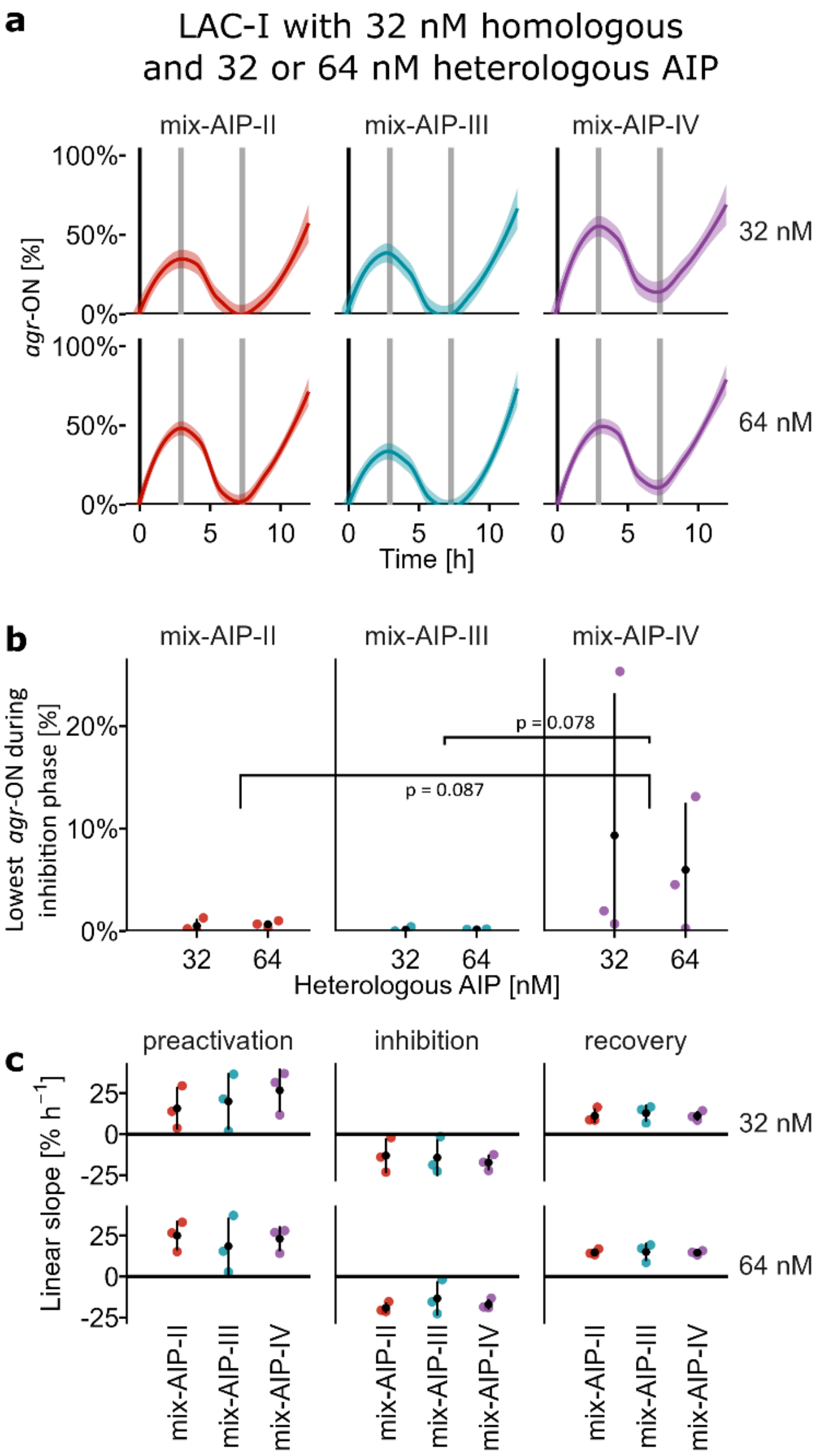

Supplementary Figure 8: Heterologous AIP exposure suppresses the *agr*-system of the LAC-I strain. a. Percentage of *agr*-ON LAC-I based on single-cell *agr*-activation (YFP fluorescence >100 arbitrary units) shown over time across exposure phases; vertical gray lines mark AIP switches. Heterologous AIP concentration is annotated. Lines show LOESS-smoothed trends, with shaded regions indicating pointwise 95% confidence intervals of the LOESS fit, colored by AIP type during the inhibition phase. On average, 610’290 cells per condition were analyzed (Supplementary Table 5). **b.** Lowest percentage of *agr*-ON cells during heterologous inhibition phase. Colored dots represent replicates; black dots and bars show mean ± SD. Statistical significance was assessed using Dunnett’s test with AIP-IV as reference, combining both heterologous AIP concentrations. **c.** The slope of per-replicate linear regression of the percentage of *agr*-ON is shown for the three experimental phases. Colored dots represent replicates; black dots and bars show mean ± SD. N = 3 independent mother-machine experiments inoculated from cultures obtained on separate days.

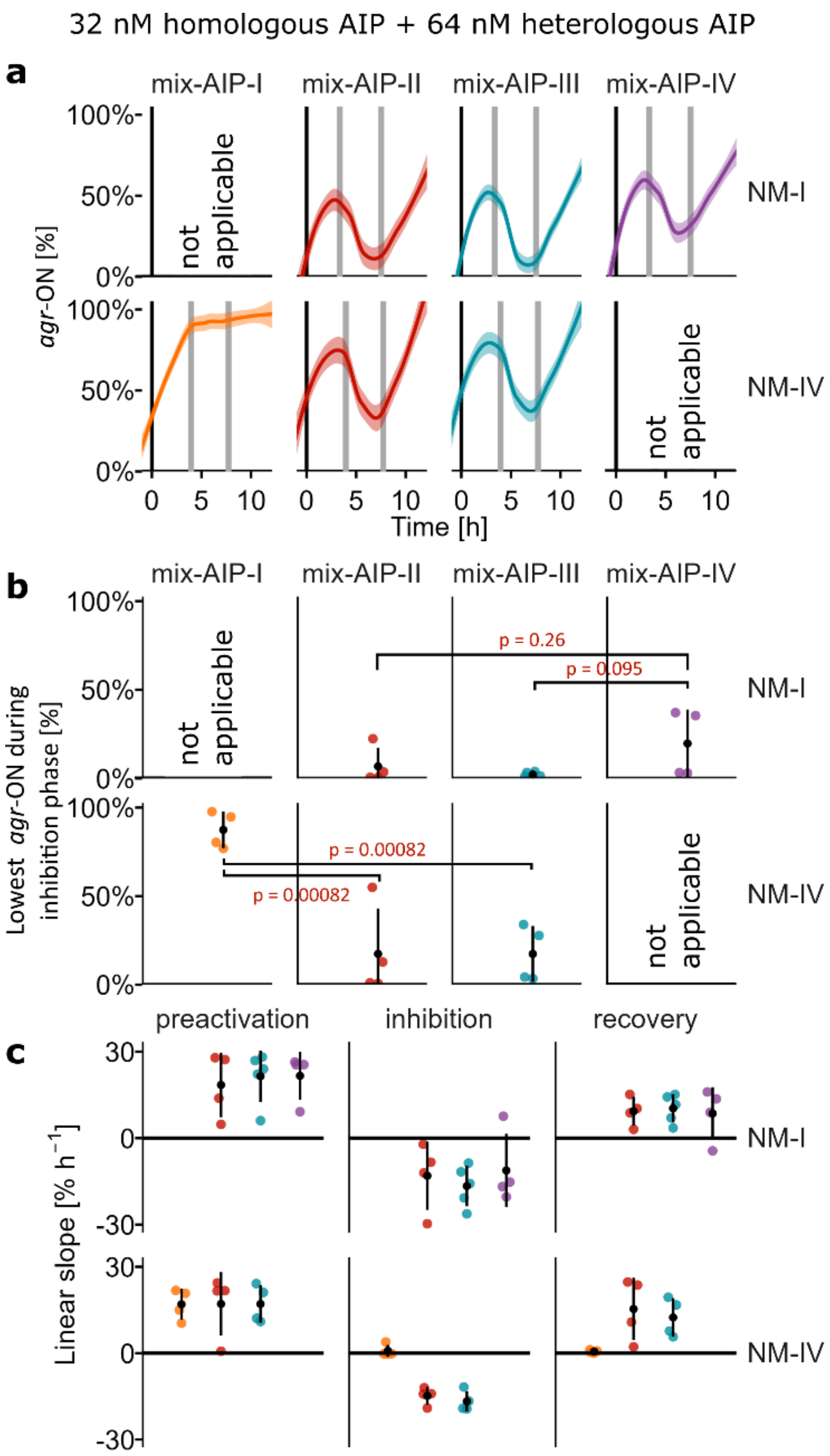

Supplementary Figure 9: 64 nM heterologous AIPs suppress pre-activated *agr*-systems. a. Percentage of *agr*-ON cells based on single-cell *agr*-activation (GFP fluorescence >100 arbitrary units) shown over time across exposure phases; vertical gray lines mark AIP switches. Lines show LOESS-smoothed trends, with shaded regions indicating pointwise 95% confidence intervals of the LOESS fit, colored by AIP type during the inhibition phase. On average, 1'897'476 cells per condition were analyzed (Supplementary Table 4). **b.** Lowest percentage of *agr*-ON cells during heterologous inhibition phase. Colored dots represent replicates; black dots and bars show mean ± SD. Statistical significance was assessed using Dunnett's test with AIP-IV and AIP-I as references for NM-I and NM-IV, respectively. **c.** The slope of per-replicate linear regression of the percentage of *agr*-ON is shown for the three experimental phases. Colored dots represent replicates; black dots and bars show mean ± SD. As NM-IV, mix-AIP-I reached full activation in preactivation phase and did not get inhibited by AIP-I, the shown slope of 0 in the consecutive phases represents no change from the preactivation phase endpoint. N = 3-5 independent mother-machine experiments inoculated from cultures obtained on separate days, details in Supplementary Table 4.

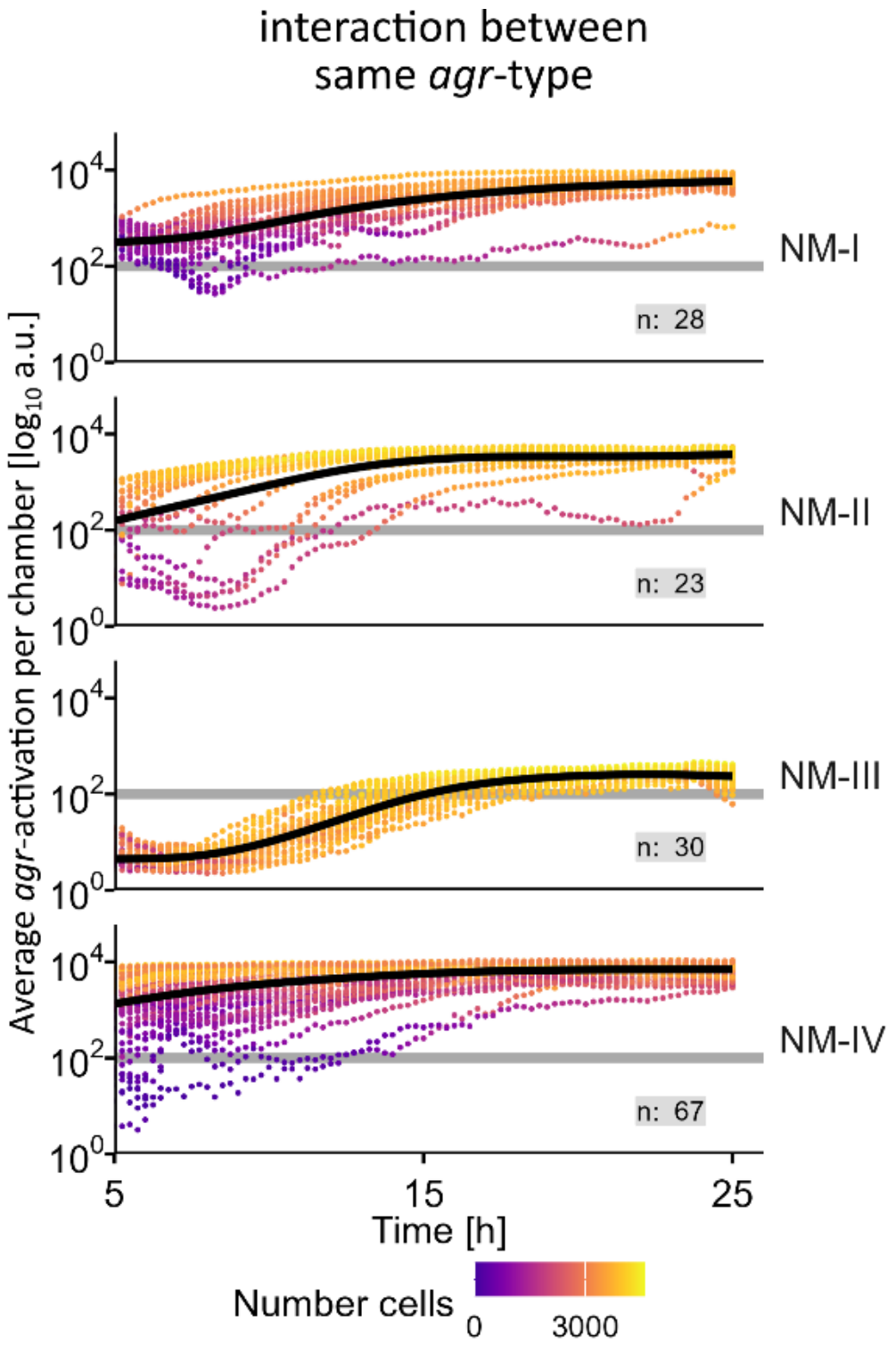

Supplementary Figure 10: Self-produced AIP led to autoinduction of the *agr*-system in the connected-chamber chips. Data is shown from connected-chamber experiments with the same congenic *agr* strain on both sides. All four strains were able to autoinduce the *agr*-system in these conditions (*agr*-activation measured as GFP fluorescence in arbitrary units, a.u.). Dot color represents cell number per chamber. Gray line indicates threshold used to classify chambers as *agr*-ON (100 a.u.). Number of chambers is annotated, stemming from 1-2 independent biological replicates.

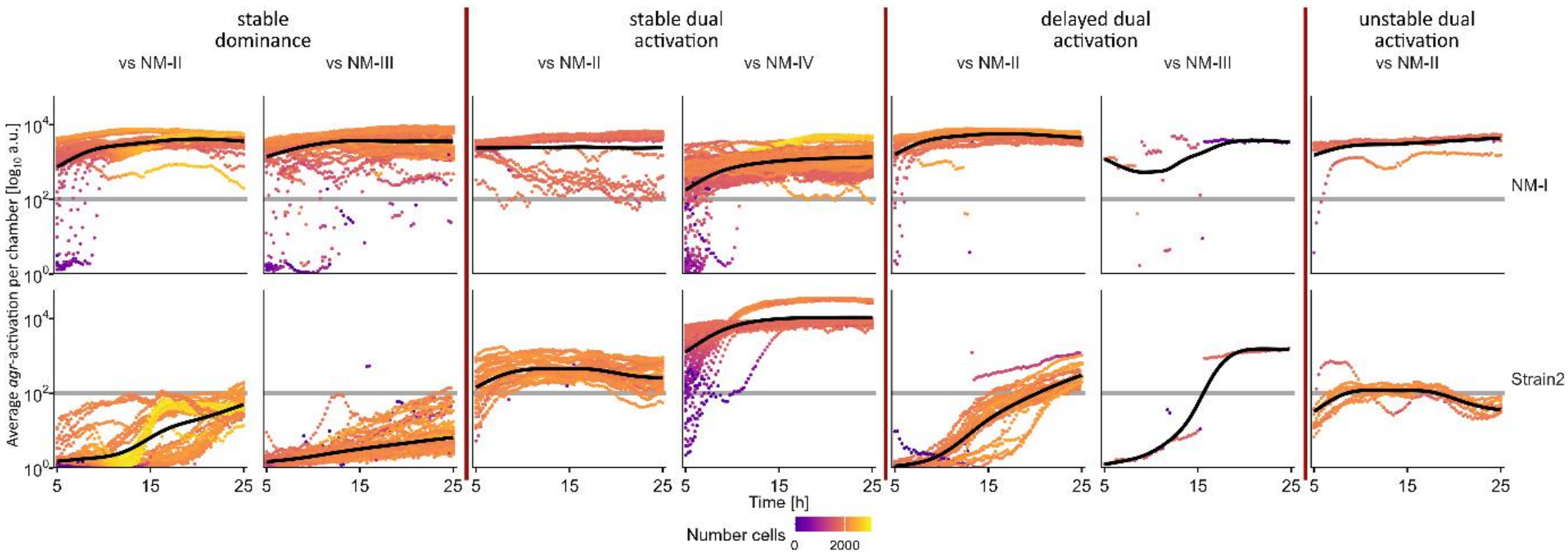

Supplementary Figure 11: Temporal *agr*-activation patterns of interacting *agr*-types in connected chambers. A. Mean *agr*-activation measured as GFP fluorescence in arbitrary units (a.u.) per chamber over time for each observed interaction category in the given congenic *agr* strain pairing annotated above the columns of plots. Interaction categories are divided with red vertical lines. The top row shows data of NM-I and the bottom row (labelled Strain2) shows data of the partner congenic strain annotated above each row in the vs statement. Dot color represents cell number per chamber. Gray line indicates threshold used to classify chambers as *agr*-ON (100 a.u.).

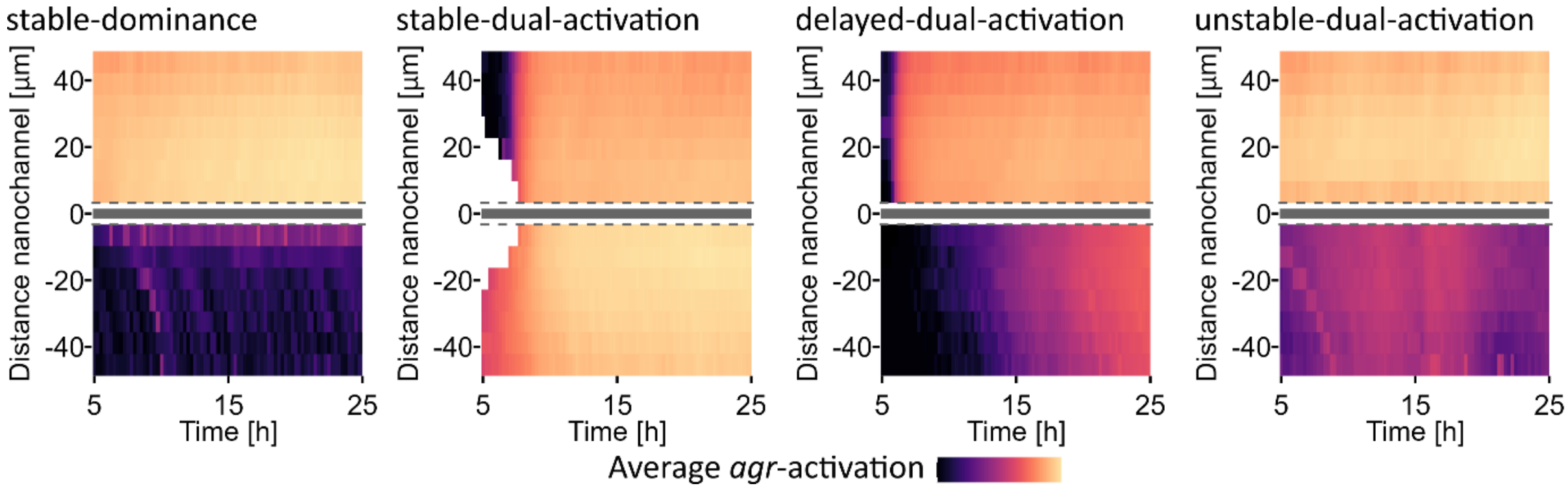

Supplementary Figure 12: Representative kymographs of the four *agr*-interaction classes. Color intensity represents average *agr*-activation measured as GFP intensity in arbitrary units per 6.5 × 6.5 µm area as a function of time and distance from the nanochannels. Gray line indicates position and width of the nanochannel. Dashed lines show the area excluded from analysis to ensure correct chamber-side assignment of segmented cells. White sections in stable-dual-activation early in time indicate not-yet filled chamber area. The NM-I strain is always displayed on positive distance from nanochannel side and the partner *agr*-type at negative distance from nanochannel. Same examples as shown in Fig. 5b.

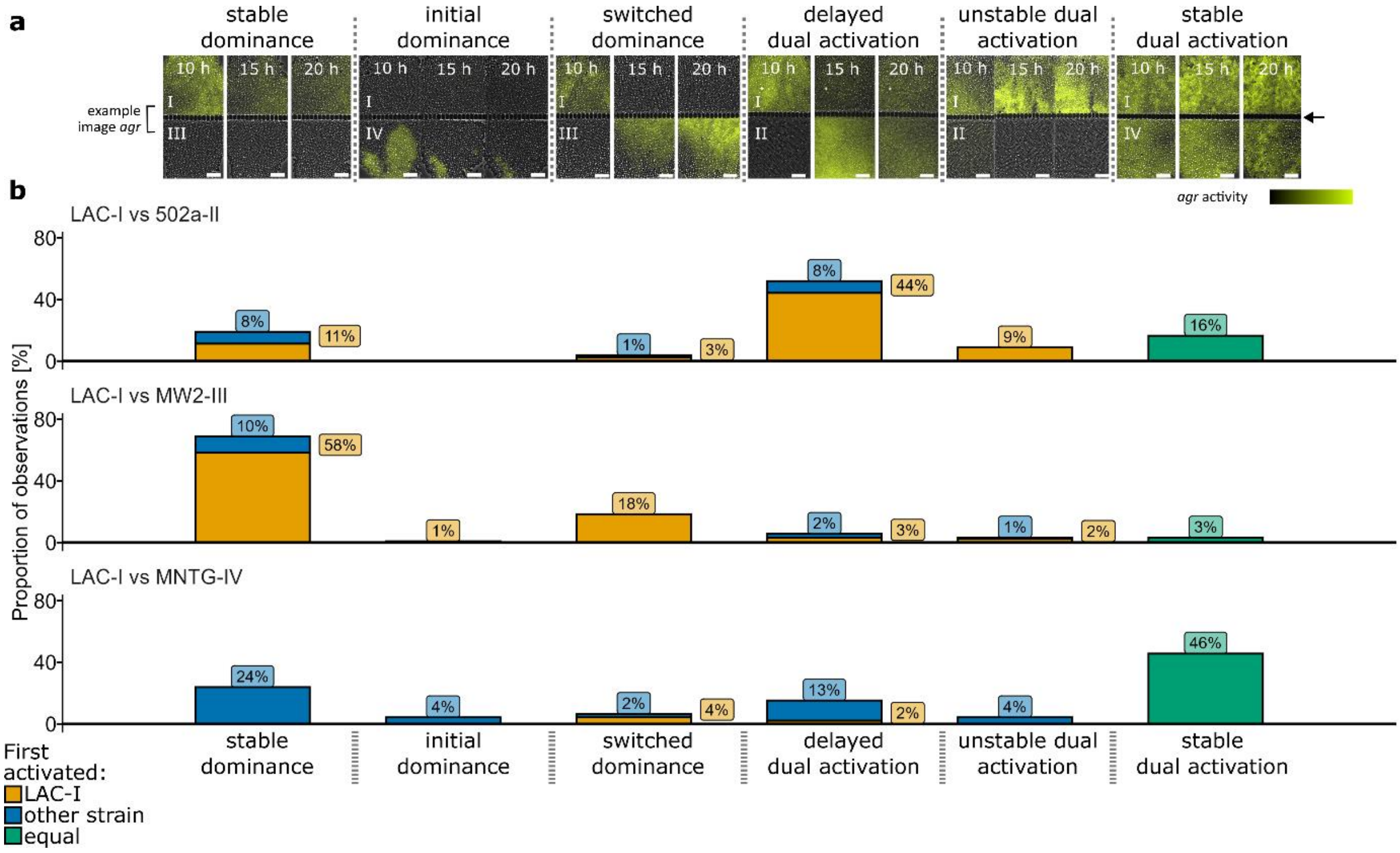

Supplementary Figure 13: Distinct *agr*-interaction classes of native-*agr* strains. a Representative image sequence showing P3-*yfp* fluorescence intensity at selected times post-inoculation (phase contrast overlaid with YFP; contrast adjusted for visualization). Arrow mark nanochannels; the two *agr*-types (representative example) are indicated on either side. Scale bar, 10 µm. **b.** Distribution of interaction classes for each pairing: stable dominance (one population persistently *agr*-ON, the other suppressed), initial dominance (short period of dominance followed by *agr*-OFF for both), switched dominance (switch of dominating type during experiment), delayed dual activation (one partner activated after a lag), unstable dual activation (fluctuating activation in one partner) and stable dual activation (both persistently *agr*-ON with minor temporal differences). The strain that consistently or initially dominated is indicated by color (orange for LAC-I, blue for the interacting strain annotated per row or green for stable-dual-activation). Proportions are annotated. Counts per class are listed in Supplementary Table 7.

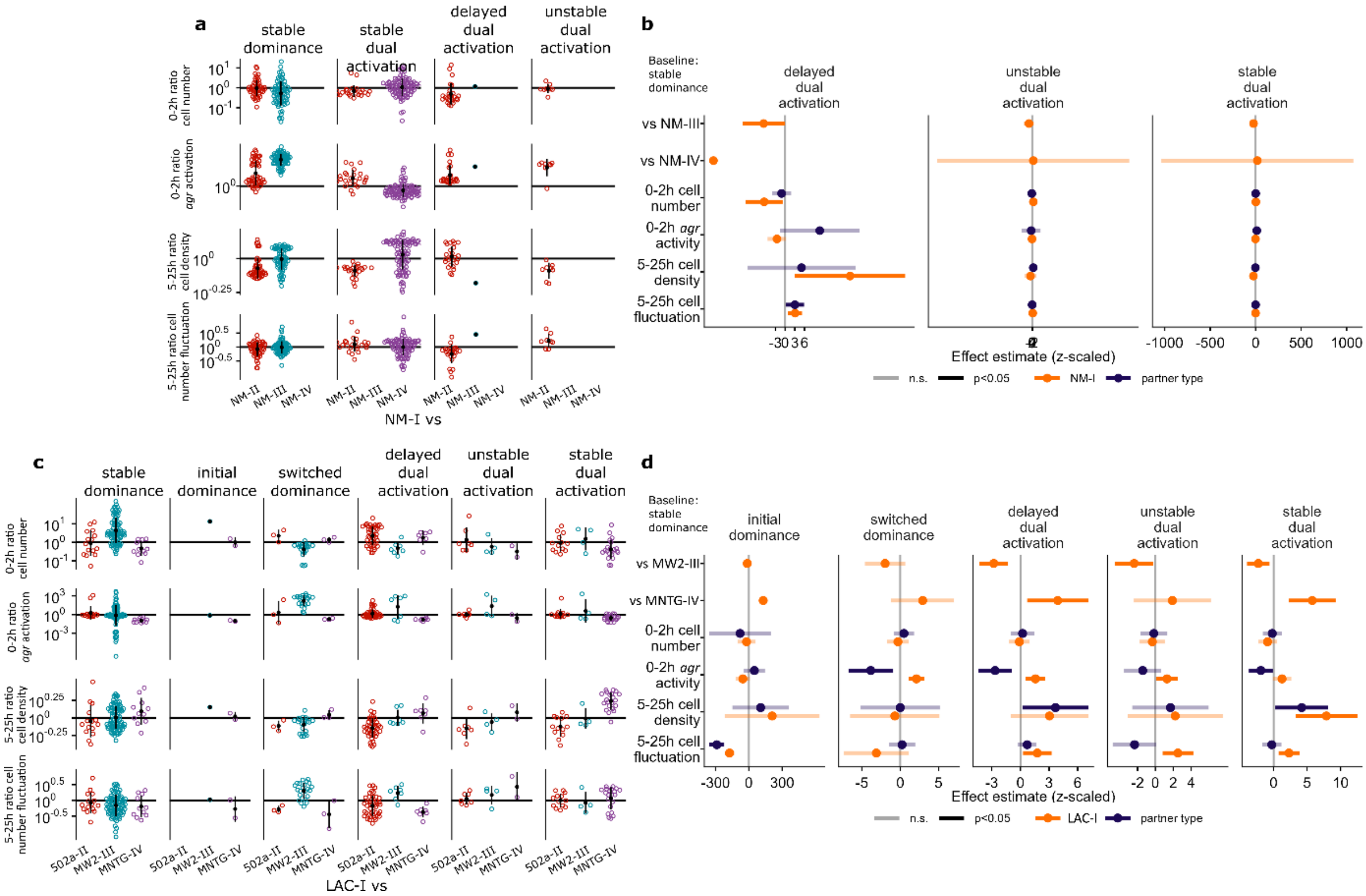

Supplementary Figure 14: Community characteristics to explain *agr*-interaction outcome. a and **c.** Four population characteristics were quantified for each connected chamber pair: (1) total cell number during early colonization (0-2 h), (2) early *agr*-activation (0-2 h), (3) average cell density during established growth (5-25 h), and (4) cell-number fluctuations during established growth (5-25 h). For each characteristic, the ratio of NM-I in a), or LAC-I for c), to the partner strain is shown. Values above one (10^0) indicate higher cell numbers or *agr*-activation of NM-I or LAC-I. Each dot represents one chamber pair, colored by partner *agr*-type. Black dots and bars indicate mean ± SD. Panels **a** and **c** show congenic and native strain data, respectively. **b** and **d.** Forest plot of multinomial regression using the four community characteristics shown in a and c to explain *agr*-interaction outcomes. Stable-dominance (the most frequent interaction class) was used as the reference class and pairings with NM-II (or 502a-II) as the reference level for the strain combination variable. All explanatory variables were z-scaled before analysis. Circles indicate effect estimates and bars denote confidence intervals. Panels **b** and **d** show congenic and native strain data, respectively. Number of observed classes are listed in Supplementary Tables 6 and 7.

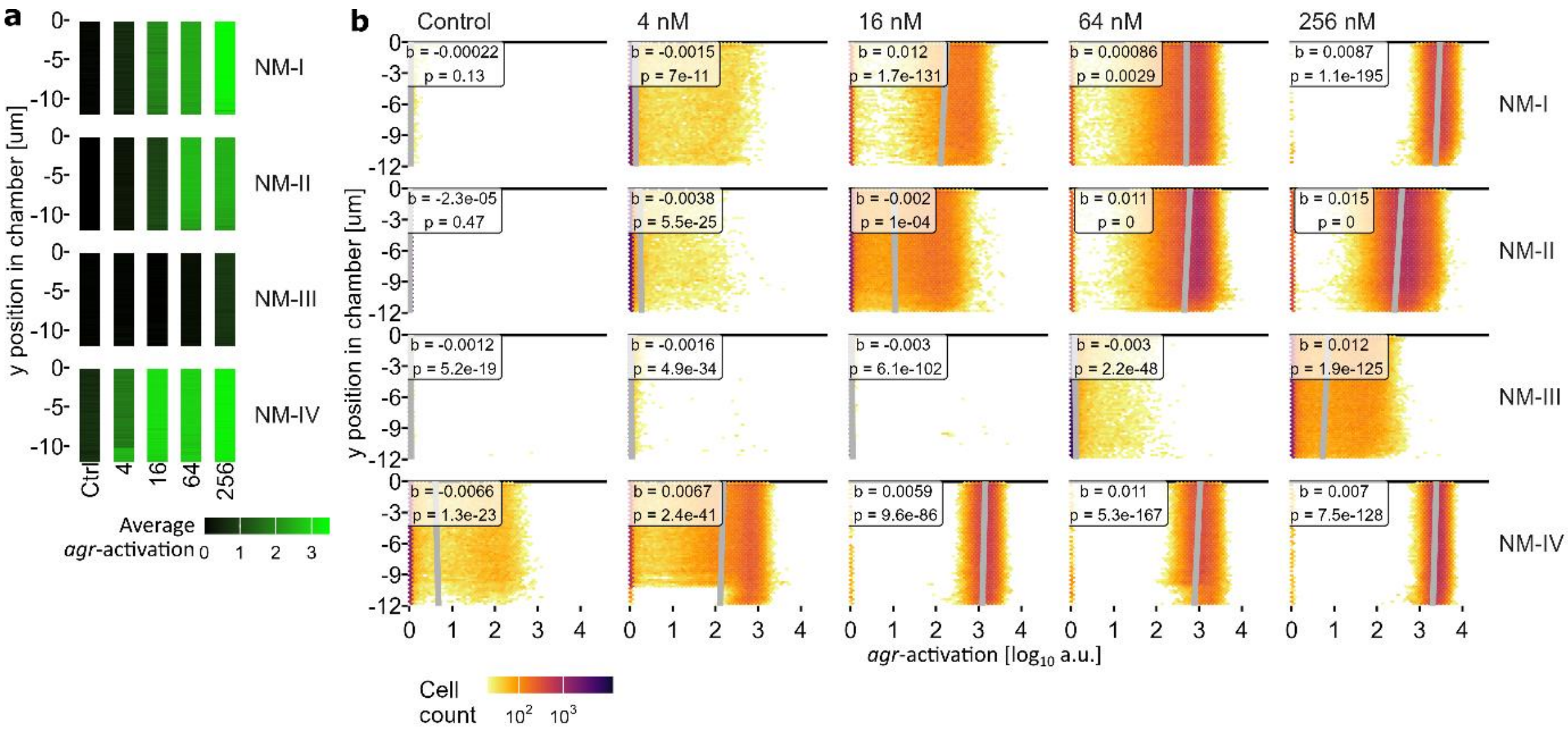

Supplementary Figure 15: No systematic effect of position of bacterial cell within mother-machine chamber on *agr*-activation. Data of the two last hours of synthetic, homologous AIP exposure phase (+AIP phase in Fig.1c) was used. **a.** The *agr*-activation (log_{10} GFP intensity in arbitrary units, a.u.) was averaged over all chambers per *agr*-type and AIP concentration and is shown as vectorial heatmap. Y-position 0 is at top of segmented chamber (approximately half depth of full chamber) and -12 value is at deepest point. **b.** X-axis shows log_{10} GFP intensity (a.u.) as function of chamber depth (same as in a.). Dot color reflects log_{10} cell counts. Gray lines indicate mixed effect regression lines of which the slope (b value, on log_{10} scale) and p-value is annotated. While p-value indicate significant differences, the slopes confirm biologically negligible changes.

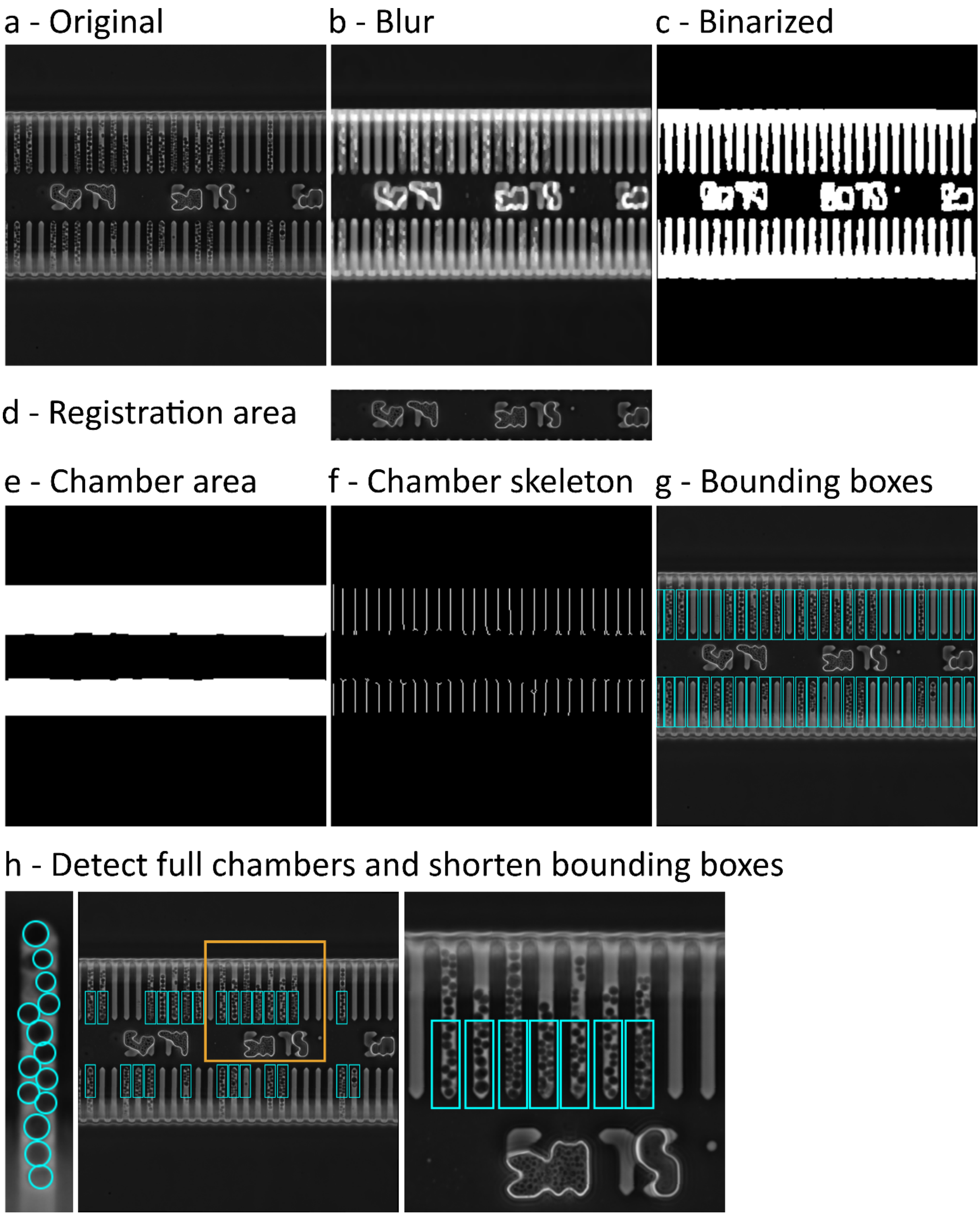

Supplementary Figure 16: Illustration of pre-processing of mother-machine images. **a.** The original image was **b.** sequentially filtered and then **c.** binarized. **d.** The area of the image used for registration was identified by detecting the part between the **e.** chamber area. The binarized image (c) was **f.** cleaned up and skeletonized to detect **g.** bounding boxes of each chamber. A **h.** Hugh transform based circle detection was performed for each chamber, chambers without any cells discarded and chamber bounding boxes shortened.

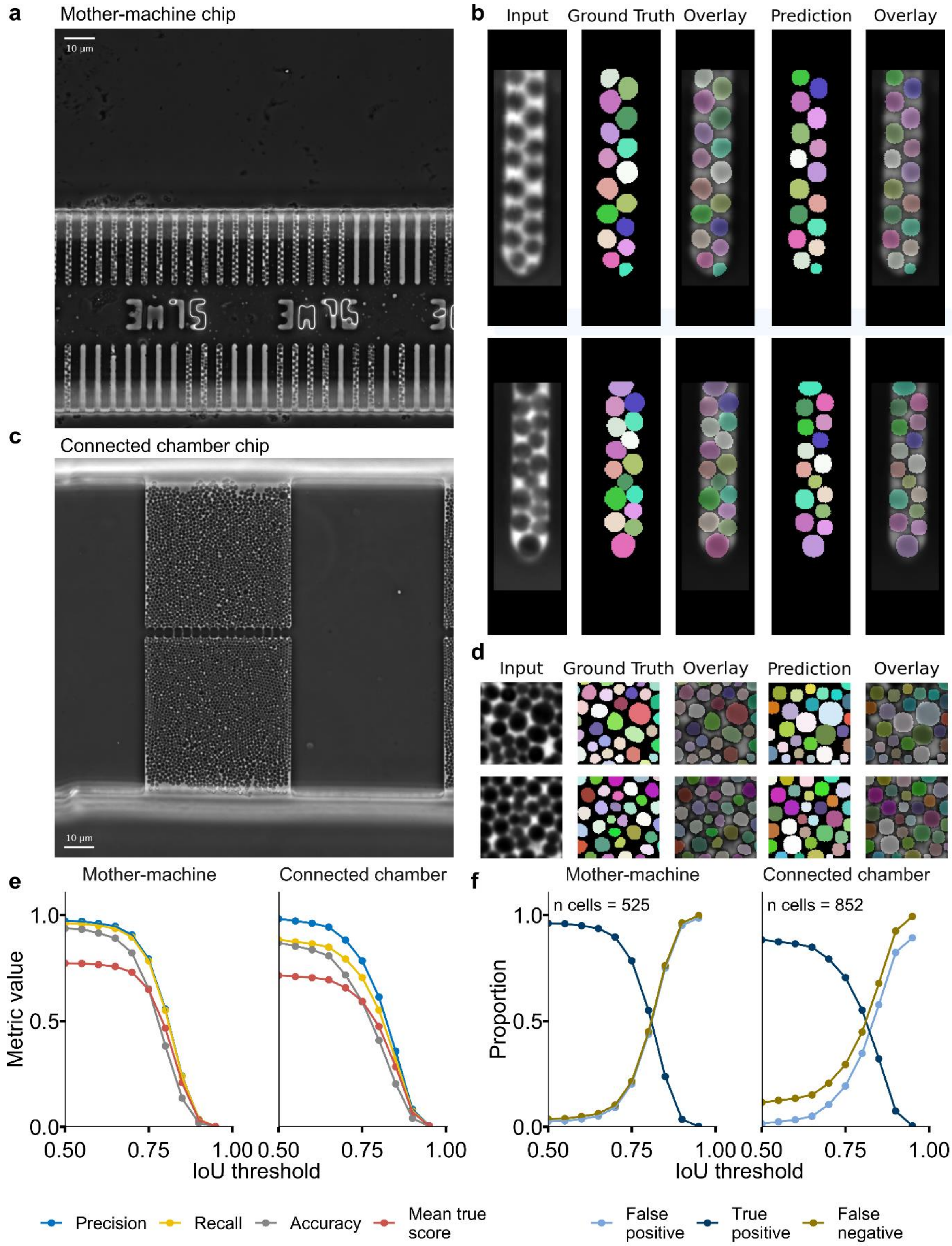

Supplementary Figure 17: StarDist segmentation quality assessment. **a.** Example image of a mother-machine microfluidic chip. Two rows of chambers can be imaged simultaneously. **b.** Example cell segmentation images of two individual mother-machine chambers using StarDist2D. **c.** Example image of a connected-chamber microfluidic chip. Media is flowing at the top and bottom and cells are embedded in the chambers. Nutrients and small molecules can diffuse through the nanochannels in the middle, but cells are retained. **d.** Example cell segmentation images of small (100 x 100 px) crops of connected-chamber chips using a separately trained StarDist2D model. **e.** Segmentation model performance quantification of the mother-machine and connected-chamber models. **f.** False positive, true positive and false negative rates of the segmentation models.

Supplementary Tables

Supplementary Table 1: Number of observed bacterial cells for each strain and AIP concentrations used in the experiments associated with Fig. 1, 2 and 3. Segmented cells are summed over all timepoints. Tracked cells have IDs that span over multiple timepoints and were derived from a subset of the replicates used for segmentation only.

		Segmented cells			Tracked cells	
**Strain**	**AIP [nM]**	**Number replicates**	**Average per replicate**	**Total**	**Number replicates**	**Total**
NM-I	Control	5	536’679	2’683’393	3	64’090
NM-I	4 nM	4	585’739	2’342’954		
NM-I	16 nM	3	610’876	1’832’627		
NM-I	64 nM	5	734’621	3’673’103	3	131’529
NM-I	256 nM	3	588’841	1’766’523		
NM-II	Control	6	699’407	4’196’443	3	146’857
NM-II	4 nM	3	1’043’577	3’130’732		
NM-II	16 nM	3	1’142’737	3’428’210		
NM-II	64 nM	7	651’768	4’562’378	3	128’800
NM-II	256 nM	5	929’346	4’646’729		
NM-III	Control	5	719’141	3’595’704	3	107’769
NM-III	4 nM	4	888’092	3’552’366		
NM-III	16 nM	4	857’833	3’431’333		
NM-III	64 nM	6	715’231	4’291’383	3	86’178
NM-III	256 nM	3	841’992	2’525’975		
NM-IV	Control	3	673’693	2’021’078	3	34’091
NM-IV	4 nM	3	822’394	2’467’181		
NM-IV	16 nM	3	683’763	2’051’288		
NM-IV	64 nM	3	577’028	1’731’083	3	106’725
NM-IV	256 nM	3	713’726	2’141’179		
	**Mean:**	**4**	**750’824**	**3’003’583**	**3**	**100’755**

Supplementary Table 2: Number of observed bacterial cells for each strain and AIP concentrations used in the experiments associated with the Supplementary Fig. 3.

		Segmented cells			Tracked cells	
**Strain**	**AIP [nM]**	**Number replicates**	**Average per replicate**	**Total**	**Number replicates**	**Total**
LAC-I	Control	7	337’070	2’359’488	3	14’080
LAC-I	4 nM	3	321’437	964’312		
LAC-I	16 nM	4	305’799	1’223’196		
LAC-I	64 nM	6	492’798	2’956’790	3	28’926
LAC-I	256 nM	3	240’879	722’637		
502a-II	Control	7	235’713	1’649’994	3	30’365
502a-II	4 nM	5	257’291	1’286’455		
502a-II	16 nM	3	251’326	753’977		
502a-II	64 nM	9	293’768	2’643’912	5	43’559
502a-II	256 nM	3	761’899	1’523’798		
MW2-III	Control	6	627’760	3’766’559	3	84’574
MW2-III	4 nM	3	525’594	1’576’783		
MW2-III	16 nM	3	650’440	1’951’319		
MW2-III	64 nM	6	438’376	2’630’253	3	102’729
MW2-III	256 nM	3	305’668	917’003		
MNTG-IV	Control	7	403’863	2’827’040	3	24’217
MNTG-IV	4 nM	3	408’851	1’226’552		
MNTG-IV	16 nM	3	162’993	488’980		
MNTG-IV	64 nM	7	250’498	1’753’489	3	9’945
MNTG-IV	256 nM	3	286’075	858’224		
	**Mean:**	**4.7**	**377’905**	**1’704’038**	**3.3**	

Supplementary Table 3: Statistical analysis results of RT-qPCR data shown in Figure 1g.

Comparison	Strain	Gene	Estimate	SE	df	t.ratio	Annotation	*p*-value
0nM - 256nM	NM-I	*agrA*	-0.91	0.21	14	-4.41	***	6.00E-04
0nM - 256nM	NM-II	*agrA*	-0.94	0.21	14	-4.55	***	4.50E-04
0nM - 256nM	NM-III	*agrA*	-0.31	0.21	14	-1.51	ns	1.50E-01
0nM - 256nM	NM-IV	*agrA*	-0.26	0.21	14	-1.25	ns	2.30E-01
0nM - 256nM	NM-I	RNAIII	-2.33	0.38	14	-6.18	***	2.40E-05
0nM - 256nM	NM-II	RNAIII	-2.94	0.38	14	-7.79	***	1.90E-06
0nM - 256nM	NM-III	RNAIII	-1.20	0.38	14	-3.17	**	6.80E-03
0nM - 256nM	NM-IV	RNAIII	-1.18	0.38	14	-3.13	**	7.50E-03
0nM - 256nM	NM-I	*psmβ2*	-2.24	0.44	14	-5.12	***	1.60E-04
0nM - 256nM	NM-II	*psmβ2*	-2.55	0.44	14	-5.81	***	4.50E-05
0nM - 256nM	NM-III	*psmβ2*	-0.22	0.44	14	-0.50	ns	6.20E-01
0nM - 256nM	NM-IV	*psmβ2*	-1.86	0.44	14	-4.25	***	8.10E-04
0nM - 256nM	NM-I	*gfp*	-1.59	0.20	14	-8.11	***	1.20E-06
0nM - 256nM	NM-II	*gfp*	-1.99	0.20	14	-10.11	***	8.20E-08
0nM - 256nM	NM-III	*gfp*	-0.55	0.20	14	-2.81	*	1.40E-02
0nM - 256nM	NM-IV	*gfp*	-1.10	0.20	14	-5.57	***	6.90E-05

Supplementary Table 4: Number of observed bacterial cells for mixed homologous and heterologous AIP exposure for the NM-I and NM-IV used in the experiments associated with Fig.4 and Supplementary Fig.10. AIP type and concentration are for the heterogeneous AIP; homologous AIP were provided at 32 nM.

Heterologous				Segmented cells	
**Strain**	**AIP type**	**AIP [nM]**	**Number replicates**	**mean per replicate**	**total**
NM-IV	mix-AIP-I	32 nM	4	286’829	1’147’315
NM-IV	mix-AIP-I	64 nM	4	402’042	1’608’169
NM-I	mix-AIP-II	32 nM	3	769’624	2’308’873
NM-IV	mix-AIP-II	32 nM	4	336’628	1’346’513
NM-I	mix-AIP-II	64 nM	4	561’977	2’247’909
NM-IV	mix-AIP-II	64 nM	4	218’375	873’499
NM-I	mix-AIP-III	32 nM	4	634’972	2’539’889
NM-IV	mix-AIP-III	32 nM	4	389’584	1’558’334
NM-I	mix-AIP-III	64 nM	5	563’956	2’819’779
NM-IV	mix-AIP-III	64 nM	4	286’791	1’147’162
NM-I	mix-AIP-IV	32 nM	5	583’727	2’918’635
NM-I	mix-AIP-IV	64 nM	4	563’409	2’253’635
		**Mean:**	**4**	**466’493**	**1’897’476**

Supplementary Table 5: Number of observed bacterial cells for mixed AIPs and concentrations used in the experiments associated with the Supplementary Fig.9. AIP type and concentration are for the heterogeneous AIP; homologous AIP were provided at 32 nM.

Heterologous			Segmented cells	
**AIP type**	**AIP [nM]**	**Number replicates**	**mean per replicate**	**total**
mix-AIP-II	32 nM	3	158’708	476’123
mix-AIP-II	64 nM	3	171’404	514’212
mix-AIP-III	32 nM	3	225’849	677’547
mix-AIP-III	64 nM	3	150’201	450’604
mix-AIP-IV	32 nM	3	304’962	914’887
mix-AIP-IV	64 nM	3	209’456	628’368
	**Mean:**	**3**	**203’430**	**610’290**

Supplementary Table 6: Number of observed chambers for each congenic *agr* strain combination as well as the number (N) and percentage of observation of each dynamic category.

Combination	Total observations	Category	N	Percentage
NM-I vs NM-II	107	stable dominance	50	46.30%
		stable dual activation	24	22.22%
		delayed dual activation	25	23.15%
		unstable dual activation	8	7.41%
NM-I vs NM-III	77	stable dominance	75	97.40%
		delayed dual activation	1	1.30%
		unclear	1	1.30%
NM-I vs NM-IV	92	stable dual activation	92	100.00%

Supplementary Table 7: Number of observed chambers for each native *agr* strain combination tested as well as the number (N) and percentage of observation of each dynamic category.

Combination	Total observations	Category	N	Percentage
LAC-I vs 502a-II	79	stable dominance	15	18.99
		switched dominance	3	3.80
		delayed dual activation	41	51.90
		unstable dual activation	7	8.86
		stable dual activation	13	16.46
LAC-I vs MW2-III	121	stable dominance	86	68.80
		initial dominance	1	0.80
		switched dominance	23	18.40
		delayed dual activation	7	5.60
		unstable dual activation	4	3.20
		stable dual activation	4	3.20
LAC-I vs MNTG-IV	46	stable dominance	11	23.91
		initial dominance	2	4.35
		switched dominance	3	6.52
		delayed dual activation	7	15.22
		unstable dual activation	2	4.35
		stable dual activation	21	45.65

Supplementary Table 8: Polymorphisms identified between the four congenic *agr* strains.

Annotation	NM-I	NM-II	NM-III	NM-IV	Class
***metQ*_1**	A	G	A	G	synonymous
**Hypothetical**	G	A	G	G	
**Hypothetical**	T	A	T	T	
**Hypothetical**	T	C	T	T	
**Hypothetical**	C	C	T	T	
dltD	A	A	G	G	non synonymous
prs	T	T	T	G	non synonymous

Supplementary Table 9: Oligonucleotides used for amplification of P3 sequence from the genetic material of the indicated strains. Strains with that same P3 sequence are listed as well.

Oligonucleotides

**EcoRI**-*P3*-F	5' – **GAATTC**GCGCG*ATGTAATTTGTATTTAATATT* – 3'
**XbaI**-*P3*-R	5' – **TCTAGA**GCGCG*CAACTATTTTCCATCACATC* – 3'

Genetic information

**gDNA source**	**P3 sequence**	**Strains with that P3 sequence**
MW2 (P3seq2)	5'–ATGTAATTTGTATTTAATATTTTAACATAAAAAAATTTACAGTTAAGAATAAAAAACGACT AGTTAAGAAAAATTGGAAAATAAATGCTTTTAGCATGTTTTAATATAACTAGATCACAGAGA TGTGATGGAAAATAGTTG–3'	NM-I; NM-II, MW2, Newman, 8325-4
RN4850 (P3seq3)	5'–ATGTAATTTGTATTTAATATTTTAACATAAAAAAATTTACAGTTAAGAATAAAAAACGACT AGTTAAGAAAAA**A**TGGAAAATAAATGCTT**A**TAGCATGTTTTAATATAACTAGATCACAGAGA TGTGATGGAAAATAGTTG–3'	NM-IV, MNTG
EG804 (P3seq4)	5'–ATGTAATTTGTATTTAATATTTTAACATAAAAAAATTTACAGTTAAGAATAAAAAACGACT AGTTAAGAAAAA**A**TGGAAAATAAATGCTTTTAGCATGTTTTAATATAACTAGATCACAGAGA TGTGATGGAAAATAGTTG–3'	NM-III, RN3984

Supplementary Table 10: List of used qPCR primers.

**Gene**	**Forward**	**Reverse**
hu	TGC AGA GCA AGC TGA TTT AA	TAC CAG TTT GAG GGT TAC G
agrA	AAC TGC ACA TAC ACG CTT ACA	GGC AAT GAG TCT GTG AGA TTT
RNAIII	GTG ATG GAA AAT AGT TGA TGA GTT GTT T	GAA TTT GTT CAC TGT GTC GAT AAT CC
psmβ2	TGA CTG GAC TAG CAG AAG CA	TAA ACC CAC ACC GTT AGC A
gfp	GAA CTT TTC ACT GGA GTT GTC CC	ATG CCG TTT CAT ATG ATC CGG

Supplementary Table 11: List of used samples from the RNA sequencing repository on European Nucleotide Archive (ENA) at EMBL-EBI under accession PRJEB107700. ID is the ENA accession number of the specific samples, each containing forward and reverse reads.

**Strain**	**Replicate**	**ID**	**Alias**
NM-I	R1	ERS28792487	R1_NM-I_t1
NM-I	R2	ERS28792502	R2_NM-I_t1
NM-I	R3	ERS28792517	R3_NM-I_t1
NM-II	R1	ERS28792488	R1_NM-II_t1
NM-II	R2	ERS28792503	R2_NM-II_t1
NM-II	R3	ERS28792518	R3_NM-II_t1
NM-III	R1	ERS28792489	R1_NM-III_t1
NM-III	R2	ERS28792504	R2_NM-III_t1
NM-III	R3	ERS28792519	R3_NM-III_t1
NM-IV	R1	ERS28792490	R1_NM-IV_t1
NM-IV	R2	ERS28792505	R2_NM-IV_t1
NM-IV	R3	ERS28792520	R3_NM-IV_t1
