# Supplementary material for: Single-cell approach dissecting agr quorum sensing dynamics in Staphylococcus aureus
Source: Nat Commun. 2026 May 20;17:8139. doi: 10.1038/s41467-026-73552-9 (PMC13458059; doi:10.1038/s41467-026-73552-9)
Supplement: Supplementary file 4 [file 41467_2026_73552_MOESM2_ESM.pdf]

Description of Additional Supplementary Files:

Supplementary Data 1: Movies relate to data shown in Fig. 1-3 and Supplementary Fig. 2, 4 and 7. For each congenic agr-strain and AIP concentration (both indicated in file name), one example movie of a single position is included. Time in HH:MM is displayed on top right, followed by indication of experimental phase (- AIP (pre), +AIP or -AIP (post)), congenic strain name and AIP concentration. Movies show phase-contrast images overlaid with fluorescent images quantifying agractivation via GFP. The same contrast scaling for both imaging channels has been applied to all frames of all movies per folder.

Supplementary Data 2: Movies relate to data shown in Supplementary Fig. 3 and 7. For each native agr-strain and AIP concentration (both indicated in file name), one example movie of a single position is included. Time in HH:MM is displayed on top right, followed by indication of experimental phase (- AIP (pre), +AIP or -AIP (post)), native agr strain name and AIP concentration. Movies show phase-contrast images overlaid with fluorescent images quantifying agractivation via YFP signal. The same contrast scaling for both imaging channels has been applied to all frames of all movies per folder.

Supplementary Data 3: Movies relate to data shown in Fig. 4 and Supplementary Fig. 9. For NM-I and NM-IV, each heterologous AIP type and concentration combination, one example movie of a single position is included. Time in HH:MM is displayed on top right, followed by indication of experimental phase, including type of heterologous AIP and its concentration. Strain name and condition are annotated at the bottom. Movies show phasecontrast images overlaid with fluorescent images quantifying *agr*-activation via GFP signal. The same contrast scaling for both imaging channels has been applied to all frames of all movies per folder.

Supplementary Data 4: Movies relate to data shown in Supplementary Fig. 8. For LAC-I and each heterologous AIP type and concentration combination, one example movie of a single position is included. Time in HH:MM is displayed on top right, followed by indication of experimental phase, including type of heterologous AIP and its concentration. Movies show phase-contrast images overlaid with fluorescent images quantifying *agr*-activation via YFP signal. The same contrast scaling for both imaging channels has been applied to all frames of all movies per folder.

Supplementary Data 5: Movies relate to data shown in Fig. 5 and Supplementary Fig. 10-12 and 14. For each observed *agr*-interaction category in the congenic *agr* strain pairings, one example movie of a single position is included. The movies are from the same positions as shown in the images in Fig. 5. Time in HH:MM is displayed on bottom right. Movies show phasecontrast images overlaid with fluorescent images quantifying *agr*-activation via GFP signal. The same contrast scaling for both imaging channels has been applied to all frames of all movies per folder.

Supplementary Data 6: Movies relate to data shown in Supplementary Fig. 13 and 14. For each observed *agr*-interaction category in the native *agr* strain pairings, one example movie of a single position is included. The movies are from the same positions as shown in the images in Supplementary Fig. 13. Time in HH:MM is displayed on bottom right. Movies show phasecontrast images overlaid with fluorescent images quantifying *agr*-activation via YFP signal. The same contrast scaling for both imaging channels has been applied to all frames of all movies per folder.

Supplementary Movie 1: Animated density histograms of the same data as shown in Fig. 1de for the congenic strains. Histograms depict *agr*-activation of bacteria observed during specific timespans (duration of 0.5 h) for each *agr*-type and AIP concentration.

Supplementary Movie 2: Animated density histograms of the same data as shown in Supplementary Fig. 3bc for the native *agr* strains. Histograms depict *agr*-activation of bacteria observed during specific timespans (duration of 0.5 h) for each *agr*-type and AIP concentration.
